# Design, synthesis, and evaluation of novel *N'*-substituted-1-(4-chlorobenzyl)-1*H*-indol-3-carbohydrazides as antitumor agents

**DOI:** 10.1080/14756366.2020.1816997

**Published:** 2020-09-28

**Authors:** Le Cong Huan, Duong Tien Anh, Pham-The Hai, Lai Duc Anh, Eun Jae Park, A Young Ji, Jong Soon Kang, Do Thi Mai Dung, Dao Thi Kim Oanh, Truong Thanh Tung, Dinh Thi Thanh Hai, Sang-Bae Han, Nguyen-Hai Nam

**Affiliations:** a Hanoi University of Pharmacy, Hanoi, Vietnam; b Thai Binh University of Medicine and Pharmacy, Thai Binh City, Vietnam; c College of Pharmacy, Chungbuk National University, Cheongju, Republic of Korea; d Bio-Evaluation Center, Korea Research Institute of Bioscience and Biotechnology, Cheongju, Republic of Korea; e Faculty of Pharmacy, PHENIKAA University, Hanoi, Vietnam; f PHENIKAA Institute for Advanced Study (PIAS), PHENIKAA University, Hanoi, Vietnam

**Keywords:** Acetohydrazides, isatin, cytotoxicity, caspase activation

## Abstract

In continuity of our search for novel anticancer agents acting as procaspase activators, we have designed and synthesised two series of (*E*)-*N*′*-*benzylidene-carbohydrazides (**4a–m**) and *(Z)-N'*-(2-oxoindolin-3-ylidene)carbohydrazides (**5a–g**) incorporating 1-(4-chlorobenzyl)-1*H*-indole core. Bioevaluation showed that the compounds, especially compounds in series **4a–m**, exhibited potent cytotoxicity against three human cancer cell lines (SW620, colon cancer; PC-3, prostate cancer; NCI-H23, lung cancer). Within series **4a–m**, compounds with 2-OH substituent (**4g–i**) exhibited very strong cytotoxicity in three human cancer cell lines assayed with IC_50_ values in the range of 0.56–0.83 µM. In particular, two compounds **4d** and **4f** bearing **4-Cl** and 4-NO_2_ substituents, respectively, were the most potent in term of cytotoxicity with IC_50_ values of 0.011–0.001 µM. In caspase activation assay, compounds **4b** and **4f** were found to activate caspase activity by 314.3 and 270.7% relative to PAC-1. This investigation has demonstrated the potential of these simple acetohydrazides, especially compounds **4b**, **4d**, and **4f**, as anticancer agents.

## 1. Introduction

Despite significant advances in anticancer therapy in the past decades, cancer remains one of the deadliest diseases with high mortality until today^1^. Current chemotherapeutic agents are mostly non-selective and are very prone to resistance by cancer cells, rendering the treatment less effective. Thus, development of more effective anticancer agents continues to be an urgent need. This also poses a great challenge due to the complexity of the disease^1^.

Apoptosis, a normal programmed cell death, is a tightly controlled and complex process in mammalian cells^2^. Dysregulation of this process, in which cells are able to escape apoptosis, is one among many causes of cancer initiation and progression^2^. Restoration of apoptosis, has therefore, become one of the most frequently used approaches in anticancer therapeutics nowadays^2^^,^^3^.

**Figure 1.**
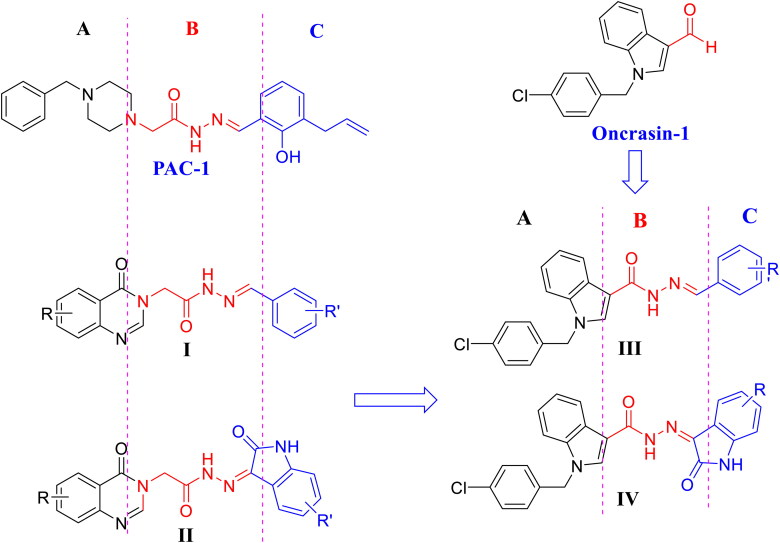
Structure of PAC-1, oncrasin-1, acetohydrazides **I**, **II** and rational design of novel (*E*)-*N'*-arylidene-1-(4-chlorobenzyl)-1*H*-indol-3-carbohydrazides **III** and (*Z*)-1-(4-chlorobenzyl)-*N*'-(2-oxoindolin-3-ylidene)-1*H*-indole-3-carbohydrazides **IV**.

The mechanism of apoptosis is extremely complex and sophisticated^4^. This process can be initiated by many different pathways, e.g. intrinsic pathway, extrinsic pathway, perforin/granzyme pathway, among others^4^. Evidence has demonstrated that both extrinsic and intrinsic pathways end at the point of the execution phase, which is considered as the final pathway of apoptosis^4^. Activation of caspases plays a pivotal role in the initiation of this final pathway. Caspases is a family of cysteine-aspartic proteases with 14 members (caspases 1–14), in which caspase-3, caspase-6, and caspase-7 play a role as effector or “executioner” caspases^5^. Among these, caspase-3 is considered as the most important one of the executioner caspases. In cancer cells, caspase-3 exists as a low-activity zymogen, procaspase-3^5^. Procaspase-3 has been shown to be overexpressed in many human cancers, such as lung cancer, melanoma, hepatoma, breast cancer, lymphoma, and neuroblastoma^6–10^. Experimental studies have shown that activation of procaspase-3 by appropriate small molecules resulted in restoration of apoptosis in cancer cells^11^. It is, therefore, targeting caspase has become an attractive approach for anticancer drug development in the last decades^12^. As a result, a number of small-molecule caspase activators have been reported^13^. Among these, PAC-1 (Figure 1), the first procaspase activating compound developed by Hergenrother’s group, demonstrated promising *in vivo* antitumor activity in human xenografted models and had been approved as an orphan drug for treatment of glioblastoma by the U.S. FDA in 2016^13^. Based on the structural pharmacophore of PAC-1, we had designed and synthesised several series of 4-oxoquinazoline derivatives (compounds **I**, **II**) and evaluated for their cytotoxicity as well as their caspase activating activity^14–17^. It was found that many compounds in series **I** and **II** were potential as caspase activators and antitumor agents^14^^,^^15^. All compounds series **I**, **II** and PAC-1 share a common *N*-acylhydrazone functionality, which has been shown to be important for the formation of a strong complex with zinc ion in the caspase active binding site^18^. Continuing our research in this direction, we have designed two novel series **III** and **IV** incorporating a 1-(4-chlorobenzyl moiety (Figure 1). The 1-(4-chlorobenzyl) moiety is a core structure of oncrasin-1, a very potential anticancer agent^19^. Oncrasin-1 has been shown to strongly reduce the growth of various cancer cells and its mechanism of cytotoxicity has been demonstrated to interfering with cellular signalling proteins, such as c-jun N-terminal kinase, Fas, Fas-associated death domain (FADD), NF-kB, among others, and increasing caspase-8 activation and, thereby inducing apoptosis^19–21^. Thus, structures **III** and **IV** can be considered either as oncrasin-1 derivatives or hybrids of oncrasin-1 and other caspase activators (PAC-1, **I**, **II**) (Figure 1). We expected that these derivatives or hybrids would result in potent anticancer activity. This paper reports the results from synthesis, biological evaluation and docking studies of the designed compounds.

## 2. Materials and methods

### 2.1. Chemistry

Thin layer chromatography which was performed using Whatman^®^ 250 µm Silica Gel GF Uniplates and visualised under UV light at 254 nm, was used to check the progress of reactions and preliminary evaluation of compounds’ homogeneity. Melting points were measured using a Gallenkamp Melting Point Apparatus (LabMerchant, London, UK) and are uncorrected. Purification of compounds was carried out using crystallisation methods and/or open silica gel column flash chromatography employing Merck silica gel 60 (240–400 mesh) as stationary phase. Nuclear magnetic resonance spectra (^1^H NMR) were recorded on a Bruker 500 MHz spectrometer with DMSO-d_6_ as solvent unless otherwise indicated. Tetramethylsilane was used as an internal standard. Chemical shifts are reported in parts per million (ppm), downfield from tetramethylsilane. Mass spectra with different ionisation modes including electron ionisation (EI), electrospray ionisation (ESI), were recorded using PE Biosystems API2000 (Perkin Elmer, Palo Alto, CA) and Mariner^®^ (Azco Biotech, Inc., Oceanside, CA) mass spectrometers, respectively. The elemental (C, H, N) analyses were performed on a Perkin Elmer model 2400 elemental analyser. All reagents and solvents were purchased from Aldrich or Fluka Chemical Corp. (Milwaukee, WI) or Merck unless noted otherwise. Solvents were used directly as purchased unless otherwise indicated.

**Scheme 1.**
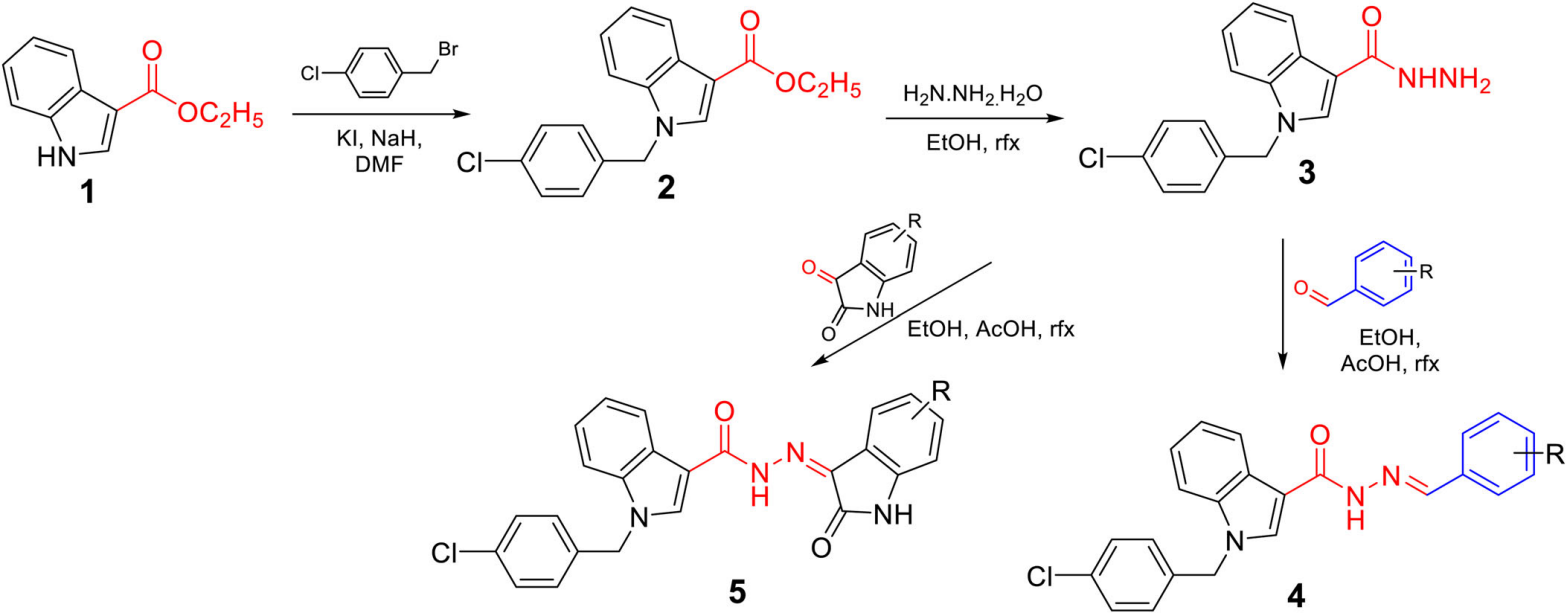
Synthesis of novel *N'*-substituted-1-(4-chlorobenzyl)-1*H*-indol-3-carbohydrazides (**4a–m**, **5a–g**).

The synthesis of *N*'-substituted-carbohydrazides incorporating 1-(4-chlorobenzyl)-1*H*-indole core (**4a–m** and **5a–g**) was carried out as illustrated in Scheme 1. Details are described below.

#### 2.1.1. General procedures for the synthesis of compounds 4a–m and 5a–g

A solution of ethyl 1*H*-indole-3-carboxylate (**1**, 2 mmol) in DMF (5 ml) was treated with NaH (4 mmol). The mixture was stirred at room temperature for 1 h, then KI (49.8 mg, 0.3 mmol) was added. After stirring for further 15 min, 1-(bromomethyl)-4-chlorobenzene (2 mmol in 1 ml of DMF) was dropped slowly into the reaction mixture. The mixture was again stirred at room temperature for 24 h until the reaction completed. After completion of the reaction, the resulting mixture was cooled, poured into ice-cold water (20 ml). The precipitates were obtained, washed with water and dried to give the corresponding intermediate ester **2**, which was used for the next step without further purification.

Compound **2** (1.5 mmol) was added to a solution of hydrazine monohydrate 80% in 1 ml ethanol. The reaction mixture was stirred at 80 °C until the reaction completed (3–4 h, checked by TLC). The resulting mixture was evaporated under reduced pressure to give the residue, which was cooled by addition of a water/ice mixture. The white solid was formed, filtered and dried to give compound **3** in quantitative yield.

The acetohydrazide **3** (1.0 mmol) was dissolved in ethanol (20 ml), two drops of glacial acetic acid, followed by benzaldehyde or isatin derivatives (1.0 mmol). The mixtures were refluxed until reaction completed. The precipitates formed were filtered and washed with ethanol (three times), the solid products were collected, dried under vacuum and recrystallised from ethanol or purified by column chromatography (MeOH:DCM) to obtain the target compounds **4a–m**, **5a–g**.

#### 2.1.2. (E)-N′-Benzylidene-1-(4-chlorobenzyl)-1H-indole-3- carbohydrazide (4a)

Yellow solid; yield: 67%. mp: 176.7–177.5 °C. *R_f_*=0.43 (DCM:MeOH = 14:1). IR (KBr, cm^–1^): 3188 (NH); 3147, 3101, 3049 (CH aren); 2930, 2891 (CH, CH_2_); 1626 (C=N); 1544, 1525 (C=C). ^1^H NMR (500 MHz, DMSO-d_6_, ppm): *δ* 11.43 (1H, s, CO-NH); 8.50–8.25 (3H, m, H-2, N=CH, H-4); 7.69–7.60 (2H, m, H-2″, H-6″); 7.58 (1H, d, *J* = 8.0 Hz, H-7); 7.47–7.41 (5H, m, H-3″, H-4″, H-5″, H-3′, H-5′); 7.34 (2H, d, *J* = 6.5 Hz, H-2′, H-6′); 7.25–7.19 (2H, m, H-5, H-6); 5.55 (2H, s, CH_2_). ^13^C NMR (125 MHz, DMSO-d_6_, ppm): *δ* 136.76; 135.15; 132.86; 130.02; 129.81; 129.30; 129.21; 127.24; 123.02; 122.04; 121.72; 11.16; 49.24. HR-MS (ESI) *m/z* 388.11826 [M + H]^+^. Anal. Calcd. for C_23_H_18_ClN_3_O (387.1138): C, 71.22; H, 4.68; N, 10.89. Found: C, 71.25; H, 4.60; N, 10.93.

#### 2.1.3. (E)-1-(4-Chlorobenzyl)-N′-(2-chlorobenzylidene)-1H-indole-3- carbohydrazide (4b)

Yellow solid; yield: 61%. mp: 179.7–181.5 °C. *R_f_*=0.52 (DCM:MeOH = 14:1). IR (KBr, cm^–1^): 3190 (NH); 3103, 3045 (CH aren); 2920, 2852 (CH, CH_2_); 1703 (C=O); 1620 (C=N); 1543, 1521 (C=C). ^1^H NMR (500 MHz, DMSO-d_6_, ppm): *δ* 11.70 (1H, s, CO-NH); 8.68 (1H, s, H-2); 8.37 (1H, s, N=CH); 8.25 (1H, m, H-4); 7.99–7.23 (11H, m, H-7, H-3″, H-4″, H-5″, H-6″, H-5′, H-6′, H-2′, H-3′, H-5, H-6); 5.56 (2H, s, CH_2_). ^13^C NMR (125 MHz, DMSO-d_6_, ppm): *δ* 136.77; 136.43; 133.29; 132.85; 132.41; 130.37; 129.73; 129.21; 128.06; 127.16; 123.11; 121.01; 121.82; 111.23; 49.29. HR-MS (ESI) *m/z* 422.07950 [M + H]^+^. Anal. Calcd. for C_23_H_17_Cl_2_N_3_O (421.0949): C, 65.41; H, 4.06; N, 9.95. Found: C, 65.44; H, 4.10; N, 9.90.

#### 2.1.4. (E)-1-(4-Chlorobenzyl)-N′-(3-chlorobenzylidene)-1H-indole-3- carbohydrazide (4c)

Yellow solid; yield: 65%. mp: 179.7–181.5 °C. *R_f_*=0.51 (DCM:MeOH = 14:1). IR (KBr, cm^–1^): 3182 (NH); 3138, 3026 (CH aren); 2922, 2852 (CH, CH_2_); 1778 (C=O); 1618 (C=N); 1544, 1521, 1489 (C=C). ^1^H NMR (500 MHz, DMSO-d_6_, ppm): *δ* 11.59 (1H, s, CO-NH); 8.49–8.25 (2H, m, H-2, N=CH); 8.23 (1H, d, *J* = 7.0 Hz, H-4); 7.76 (1H, s, H-2″); 7.65–7.61 (1H, m, H-6″); 7.58 (1H, d, *J* = 7.5 Hz, H-7); 7.50–7.44 (4H, m, H-5″, H-6″, H-3′, H-5′); 7.33 (2H, d, *J* = 6.5 Hz, H-2′, H-6′); 7.25–7.19 (2H, m, H-5, H-6); 5.55 (2H, s, CH_2_). ^13^C NMR (125 MHz, DMSO-d_6_, ppm): *δ* 137.44; 136.80; 134.13; 132.84; 131.18; 129.71; 129.65; 129.20; 126.60; 125.87; 123.09; 121.99; 121.79; 111.22; 49.26. HR-MS (ESI) *m/z* 422.07928 [M + H]^+^. Anal. Calcd. for C_23_H_17_Cl_2_N_3_O (421.0749): C, 65.41; H, 4.06; N, 9.95. Found: C, 65.49; H, 4.01; N, 9.97.

#### 2.1.5. (E)-1-(4-Chlorobenzyl)-N′-(4-chlorobenzylidene)-1H-indole-3- carbohydrazide (4d)

Yellow solid; yield: 70%. mp: 179.7–181.5 °C. *R_f_*=0.56 (DCM:MeOH = 14:1). IR (KBr, cm^–1^): 3194 (NH); 3101, 3053 (CH aren); 2920, 2852 (CH, CH_2_); 1708 (C=O); 1625 (C=N); 1546, 1523, 1489 (C=C). ^1^H NMR (500 MHz, DMSO-d_6_, ppm): *δ* 11.49 (1H, s, CO-NH); 8.34–8.24 (3H, m, H-2, N=CH, H-4); 7.58 (1H, d, *J* = 8.0 Hz, H-7); 7.51 (2H, d, *J* = 7.0 Hz, H-2″, H-6″); 7.45 (2H, d, *J* = 7.0 Hz, H-3″, H-5″); 7.44 (2H, d, *J* = 6.5 Hz, H-3′, H-5′); 7.33 (2H, d, *J* = 7.0 Hz, H-2′, H-6′); 7.25–7.19 (2H, m, H-5, H-6); 5.55 (2H, s, CH_2_). ^13^C NMR (125 MHz, DMSO-d_6_, ppm): *δ* 136.72; 134.42; 134.10; 132.86; 129.80; 129.37; 129.22; 128.85; 123.06; 122.05; 121.77; 111.21; 49.26. HR-MS (ESI) *m/z* 422.07980 [M + H]^+^. Anal. Calcd. for C_23_H_17_Cl_2_N_3_O (421.0749): C, 65.41; H, 4.06; N, 9.95. Found: C, 65.47; H, 4.16; N, 9.90.

#### 2.1.6. (E)-1-(4-Chlorobenzyl)-N′-(2,6-dichlorobenzylidene)-1H- indole-3-carbohydrazide (4e)

Yellow solid; yield: 58%. mp: 179.7–181.5 °C. *R_f_*=0.58 (DCM:MeOH = 14:1). IR (KBr, cm^–1^): 3184 (NH); 3142, 3020 (CH aren); 2922, 2852 (CH, CH_2_); 1793 (C=O); 1616 (C=N); 1581, 1537, 1517 (C=C). ^1^H NMR (500 MHz, DMSO-d_6_, ppm): *δ* 11.80 (1H, s, CO-NH), 8.48 (1H, s, H-2); 8.20 (1H, s, N=CH); 8.19 (1H, d, *J* = 8.0 Hz, H-4); 7.51 (1H, d, *J* = 8.0 Hz, H-7); 7.31 (2H, d, *J* = 7.5 Hz, H-3″, H-5″); 7.24–7.17 (5H, m, H-5, H-2′, H-3′, H-5′, H-6′); 6.90 (2H, t, *J* = 7.5 Hz, H-6, H-4″); 5.56 (2H, s, CH_2_). ^13^C NMR (125 MHz, DMSO-d_6_, ppm): *δ* 155.79; 147.50; 137.13; 136.65; 131.51; 129.34; 129.21; 127.51; 126.78; 122.89; 121.41; 119.43; 118.31; 116.15; 112.59; 33.87. HR-MS (ESI) *m/z* 456.04019 [M + H]^+^. Anal. Calcd. for C_23_H_16_Cl_3_N_3_O (455.0359): C, 60.48; H, 3.53; N, 9.20. Found: C, 60.40; H, 3.56; N, 9.21.

#### 2.1.7. (E)-1-(4-Chlorobenzyl)-N′-(4-nitrobenzylidene)-1H-indole-3- carbohydrazide (4f)

Yellow solid; yield: 55%. mp: 179.7–181.5 °C. *R_f_*=0.62 (DCM:MeOH = 14:1). IR (KBr, cm^–1^): 3444 (NH); 3174, 3059 (CH aren); 2920, 2850 (CH, CH_2_); 1708 (C=O); 1637 (C=N); 1571, 1510 (C=C). ^1^H NMR (500 MHz, DMSO-d_6_, ppm): *δ* 11.78 (1H, s, CO-NH); 8.39 (1H, s, N=CH); 8.31–8.24 (3H, m, H-4, H-3″, H-5″); 7.94 (2H, d, *J* = 6.5 Hz, H-2″, H-6″); 7.58 (1H, d, *J* = 8.5 Hz, H-7); 7.44 (2H, d, *J* = 8.0 Hz, H-3′, H-5′); 7.33 (2H, d, *J* = 8.0 Hz, H-2′, H-6′); 7.26–7.20 (2H, m, H-5, H-6); 5.57 (2H, s, CH_2_). ^13^C NMR (125 MHz, DMSO-d_6_, ppm): *δ* 147.95; 141.59; 136.70; 132.86; 129.77; 129.22; 128.09; 124.55; 123.18; 121.98; 121.91; 111.29; 49.32. HR-MS (ESI) *m/z* 433.10309 [M + H]^+^. Anal. Calcd. for C_23_H_17_ClN_4_O_3_ (432.0989): C, 63.82; H, 3.96; N, 12.94. Found: C, 63.83; H, 3.97; N, 12.92.

#### 2.1.8. (E)-1-(4-Chlorobenzyl)-N′-(2-hydroxybenzylidene)-1H-indole- 3-carbohydrazide (4g)

Yellow solid; yield: 68%. mp: 179.7–181.5 °C. *R_f_*=0.51 (DCM:MeOH = 14:1). IR (KBr, cm^–1^): 3282 (NH); 3049 (CH aren); 2920, 2852 (CH, CH_2_); 1620 (C=N); 1521, 1487 (C=C). ^1^H NMR (500 MHz, DMSO-d_6_, ppm): *δ* 11.50 (1H, s, CO-NH), 8.52 (1H, s, H-2), 8.22 (1H, s, N=CH), 8.20 (1H, d, *J* = 8.0 Hz, H-4), 7.61–7.43 (4H, m, H-3″, H-4″, H-5″, H-6″), 7.32–7.16 (5H, m, H-7, H-2′, H-3′, H-5′, H-6′), 6.95–6.92 (2H, m, H-5, H-6), 5.53 (2H, s, CH_2_). ^13^C NMR (125 MHz, DMSO-d_6_, ppm): *δ* 131.24; 129.50; 129.21; 129.14; 122.82; 121.84; 121.48; 121.34; 119.74; 116.81; 112.52. HR-MS (ESI) *m/z* 404.11316 [M + H]^+^. Anal. Calcd. for C_23_H_18_ClN_3_O_2_ (403.1088): C, 68.40; H, 4.49; N, 10.40. Found: C, 68.42; H, 4.47; N, 10.41.

#### 2.1.9. (E)-N′-(4-Allyl-2-hydroxybenzylidene)-1-(4-chlorobenzyl)-1H- indole-3-carbohydrazide (4h)

Yellow solid; yield: 58%. mp: 179.7–181.5 °C. *R_f_*=0.47 (DCM:MeOH = 14:1). IR (KBr, cm^–1^): 3323 (NH); 3199 (OH); 3124, 3025 (CH aren); 2920 (CH, CH_2_); 1678 (C=O); 1620 (C=N); 1566, 1519 (C=C). ^1^H NMR (500 MHz, DMSO-d_6_, ppm): *δ* 11.85 (1H, s, CO-NH); 8.59 (1H, s, H-2); 8.51 (1H, s, N=CH); 8.26 (1H, s, H-4); 7.57–7.55 (2H, m, H-7, H-6″); 7.45–7.39 (3H, m, H-6″, H-3′, H-5′); 7.29–7.18 (5H, m, H-3″, H-2′, H-6′, H-5, H-6); 5.51 (1H, s, CH_2_); 5.08–5.01 (3H, m, –CH_2_–CH=CH_2_); 3.41–3.35 (2H, m, –CH_2_–CH=CH_2_). ^13^C NMR (125 MHz, DMSO-d_6_, ppm): *δ* 166.55; 136.65; 134.28; 132.82; 129.65; 129.17; 123.06; 122.04; 121.85; 116.91; 116.29; 116.18; 115.93; 111.19. HR-MS (ESI) *m/z* 444.14413 [M + H]^+^. Anal. Calcd. for C_26_H_22_ClN_3_O_2_ (443.1423): C, 70.35; H, 5.00; N, 9.47. Found: C, 70.33; H, 5.01; N, 9.49.

#### 2.1.10. (E)-1-(4-Chlorobenzyl)-N′-(2-hydroxy-4- methoxybenzylidene)-1H-indole-3-carbohydrazide (4i)

Yellow solid; yield: 65%. mp: 179.7–181.5 °C. *R_f_*=0.49 (DCM:MeOH = 14:1). IR (KBr, cm^–1^): 3576 (NH); 3319 (OH); 3174, 3043 (CH aren); 2922 (CH, CH_2_); 1683 (C=O); 1624 (C=N); 1564, 1519 (C=C). ^1^H NMR (500 MHz, DMSO-d_6_, ppm): *δ* 11.58 (1H, s, CO-NH); 8.44 (1H, s, H-2); 8.19 (1H, s, N=CH); 8.19 (1H, d, *J* = 7.0 Hz, H-4); 7.49 (1H, d, *J* = 8.0 Hz, H-7); 7.41 (1H, d, *J* = 7.5 Hz, H-5″); 7.22–7.15 (6H, m, H-2′, H-6′, H-3′, H-5′, H-5, H-6); 6.52 (1H, d, *J* = 9.0 Hz, H-5″); 6.51 (1H, s, H-3″); 5.53 (2H, s, CH_2_); 3.78 (3H, s, OCH_3_). ^13^C NMR (125 MHz, DMSO-d_6_, ppm): *δ* 162.10; 136.59; 126.84; 122.77; 121.46; 121.27; 108.76; 106.76; 101.67; 55.76. HR-MS (ESI) *m/z* 434.12378 [M + H]^+^. Anal. Calcd. for C_24_H_20_ClN_3_O_3_ (433.1193): C, 66.44; H, 4.65; N, 9.68. Found: C, 66.42; H, 4.65; N, 9.69.

#### 2.1.11. (E)-1-(4-Chlorobenzyl)-N′-(3-hydroxy-4- methoxybenzylidene)-1H-indole-3-carbohydrazide (4j)

Yellow solid; yield: 59%. mp: 179.7–181.5 °C. *R_f_*=0.48 (DCM:MeOH = 14:1). IR (KBr, cm^–1^): 3523 (NH); 3219 (CH aren); 2924, 2852 (CH, CH_2_); 1708 (C=O); 1604 (C=N); 1575, 1510 (C=C). ^1^H NMR (500 MHz, DMSO-d_6_, ppm): *δ* 8.33 (1H, s, H-2); 8.19 (1H, d, *J* = 9.0 Hz, H-4); 7.75–7.53 (1H, m, H-7); 7.40–7.38 (3H, m, H-3′, H-5′, H-2″); 7.30–7.28 (3H, m, H-5″, H-2′, H-6′); 7.23–7.21 (3H, m, H-4″, H-5, H-6); 5.52 (2H, s, CH_2_); 3.78 (3H, s, OCH_3_). ^13^C NMR (125 MHz, DMSO-d_6_, ppm): *δ* 164.48; 136.65; 136.62; 136.01; 132.78; 129.62; 129.13; 126.79; 122.19; 121.30; 111.68; 106.78; 59.62. HR-MS (ESI) *m/z* 434.12369 [M + H]^+^. Anal. Calcd. for C_24_H_20_ClN_3_O_3_ (433.1193): C, 66.44; H, 4.65; N, 9.68. Found: C, 66.45; H, 4.63; N, 9.66.

#### 2.1.12. (E)-1-(4-Chlorobenzyl)-N′-(4-methoxybenzylidene)-1H- indole-3-carbohydrazide (4k)

Yellow solid; yield: 66%. mp: 179.7–181.5 °C. *R_f_*=0.42 (DCM:MeOH = 14:1). IR (KBr, cm^–1^): 3450 (NH); 3188, 3101, 3041 (CH aren); 2910, 2843 (CH, CH_2_); 1620 (C=N); 1552, 1510 (C=C). ^1^H NMR (500 MHz, DMSO-d_6_, ppm): *δ* 11.47 (1H, s, CO-NH); 8.50–8.15 (3H, m, H-2, N=CH, H-4); 7.73–7.57 (3H, m, H-7, H-2″, H-6″); 7.45–7.39 (4H, m, H-2′, H-6′, H-3′, H-5′); 7.24–7.18 (2H, m, H-5, H-6); 7.01 (2H, d, *J* = 8.0 Hz, H-3″, H-5″); 5.54 (2H, s, CH_2_); 3.82 (3H, s, OCH_3_). ^13^C NMR (125 MHz, DMSO-d_6_, ppm): *δ* 160.92; 136.76; 132.84; 129.80; 129.21; 128.78; 127.74; 122.96; 122.04; 121.64; 114.80; 55.76; 49.23. HR-MS (ESI) *m/z* 418.12897 [M + H]^+^. Anal. Calcd. for C_24_H_20_ClN_3_O_2_ (417.1244): C, 68.98; H, 4.82; N, 10.06. Found: C, 68.97; H, 4.88; N, 10.03.

#### 2.1.13. (E)-1-(4-Chlorobenzyl)-N′-(4-(dimethylamino)benzylidene)- 1H-indole-3-carbohydrazide (4l)

Yellow solid; yield: 72%. mp: 179.7–181.5 °C. *R_f_*=0.56 (DCM:MeOH = 14:1). IR (KBr, cm^–1^): 3210 (NH); 3091, 3062 (CH aren); 2906, 2802 (CH, CH_2_); 1708 (C=O); 1610 (C=N); 1556, 1523 (C=C). ^1^H NMR (500 MHz, DMSO-d_6_, ppm): *δ* 11.19, 11.01 (0.6H, 0.4H, 2s, CO-NH); 8.50–7.96 (3H, m, H-2, N=CH, H-4); 7.56–7.32 (7H, m, H-7, H-2″, H-6″, H-2′, H-6′, H-3′, H-5′); 7.23–7.17 (2H, m, H-5, H-6); 6.75 (2H, d, *J* = 7.0 Hz, H-3″, H-5″); 5.53 (2H, s, CH_2_); 2.98 (6H, s, N(CH_3_)_2_). ^13^C NMR (125 MHz, DMSO-d_6_, ppm): *δ* 151.67; 136.74; 132.85; 131.60; 129.73; 129.21; 128.51; 122.90; 122.53; 122.16; 121.57; 112.37; 49.20; 31.15. HR-MS (ESI) *m/z* 431.16071 [M + H]^+^. Anal. Calcd. for C_25_H_23_ClN_4_O (430.1560): C, 69.68; H, 5.38; N, 13.00. Found: C, 69.67; H, 5.36; N, 13.01.

#### 2.1.14. (E)-1-(4-Chlorobenzyl)-N′-(3,4,5-trimethoxybenzylidene)-1H- indole-3-carbohydrazide (4m)

Yellow solid; yield: 60%. mp: 179.7–181.5 °C. *R_f_*=0.50 (DCM:MeOH = 14:1). IR (KBr, cm^–1^): 3180 (NH); 3028 (CH aren); 2922, 2850 (CH, CH_2_); 1784 (C=O); 1612 (C=N); 1550, 1521 (C=C). ^1^H NMR (500 MHz, DMSO-d_6_, ppm): *δ* 11.56 (1H, s, CO-NH); 8.50–8.10 (3H, m, H-2, N=CH, H-4); 7.53 (1H, m, H-7); 7.40 (2H, d, *J* = 8.5 Hz, H-2″, H-6″); 7.26–7.18 (4H, m, H-2′, H-6′, H-5, H-6); 7.01 (2H, s, H-2″, H-6″); 5.56 (2H, s, CH_2_); 3.81 (9H, s, OCH_3_). ^13^C NMR (125 MHz, DMSO-d_6_, ppm): *δ* 153.65; 139.29; 136.92; 132.71; 130.76; 129.15; 123.02; 122.05; 121.70; 111.16; 104.47; 60.58; 56.29; 49.27. HR-MS (ESI) *m/z* 478.15005 [M + H]^+^. Anal. Calcd. for C_26_H_24_ClN_3_O_4_ (477.1455): C, 65.34; H, 5.06; CN, 8.79. Found: C, 65.38; H, 5.08; CN, 8.77.

#### 2.1.15. (Z)-1-(4-Chlorobenzyl)-N′-(2-oxoindolin-3-ylidene)-1H- indole-3-carbohydrazide (5a)

Yellow solid; yield: 67%. mp: 176.7–177.5 °C. *R_f_*=0.47 (DCM:MeOH = 14:1). IR (KBr, cm^–1^): 3238 (NH); 3115, 3057 (CH aren); 2926, 2864 (CH, CH_2_); 1710 (C=O); 1660 (C=N); 1610, 1543, 1490 (C=C). ^1^H NMR (500 MHz, DMSO-d_6_, ppm): *δ* 13.25, 11.21 (0.54H, 0.46H, 2s, CONH); 11.97, 11.94 (0.6H, 0.4H, 2s, NH-isatin); 8.59, 7.62 (0.43H, 0.57H, 2s, H-2); 8.25–8.11 (2H, m, H-4, H-4′); 7.63–7.61 (1H, d, *J* = 7.5 Hz, H-7); 7.36–7.28 (4H, m, H-3′, H-5′, H-6″, H-7″); 7.18–7.10 (3H, m, H-5, H-2′, H-6′); 7.10–6.94 (1H, m, H-6); 4.95, 4.91 (1.1H, 0.9H, 2s, CH_2_). ^13^C NMR (125 MHz, DMSO-d_6_, ppm): *δ* 168.18; 162.11; 134.67; 133.17; 132.01; 131.76; 130.22; 129.58; 126.88; 124.19; 123.41; 122.47; 122.28; 121.36; 120.70; 116.13; 113.45; 111.19; 110.53; 108.47, 42.93. HR-MS (ESI) *m/z* 429.10867 [M + H]^+^. Anal. Calcd. for C_24_H_17_ClN_4_O_2_ (428.1040): C, 67.21; H, 4.00; N, 13.06. Found: C, 67.20; H, 4.05; N, 13.07.

#### 2.1.16. (Z)-1-(4-Chlorobenzyl)-N′-(5-fluoro-2-oxoindolin-3- ylidene)-1H-indole-3-carbohydrazide (5b)

Yellow solid; yield: 61%. mp: 176.7–177.5 °C. *R_f_*=0.53 (DCM:MeOH = 14:1). IR (KBr, cm^–1^): 3272 (NH); 3113, 3057 (CH aren); 2976, 2927 (CH, CH_2_); 1697 (C=O); 1668 (C=N); 1645, 1581, 1523 (C=C). ^1^H NMR (500 MHz, DMSO-d_6_, ppm): *δ* 13.72 (1H, s, CONH); 12.48 (s, NH-isatin, 1H); 8.77 (1H, s, H-2); 8.66 (1H, d, *J* = 7.0 Hz, H-4); 8.00 (1H, s, H-4″); 7.98 (1H, d, *J* = 7.5 Hz, H-7); 7.98–7.82 (4H, m, H-6″, H-7″, H-3′, H-5′); 7.70–7.64 (3H, m, H-5, H-2′, H-6′); 7.49–7.47 (1H, m, H-6); 5.46 (2H, s, CH_2_). ^13^C NMR (125 MHz, DMSO-d_6_, ppm): *δ* 162.03; 160.71; 158.81; 139.10; 137.18; 135.54; 134.10; 130.22; 129.59; 126.72; 123.72; 122.52; 122.10; 121.39; 118.02; 113.82; 112.31; 108.55; 108.19; 42.85. HR-MS (ESI) *m/z* 447.09891 [M + H]^+^. Anal. Calcd. for C_24_H_16_ClFN_4_O_2_ (446.0946): C, 64.51; H, 3.61; N, 12.54. Found: C, 64.50; H, 3.66; N, 12.56.

#### 2.1.17. (Z)-N′-(5-chloro-2-oxoindolin-3-ylidene)-1-(4-chlorobenzyl)-1H-indole-3-carbohydrazide (5c)

Yellow solid; yield: 66%. mp: 176.7–177.5 °C. *R_f_*=0.51 (DCM:MeOH = 14:1). IR (KBr, cm^–1^): 3433 (NH); 3176, 3059 (CH aren); 2926, 2868 (CH, CH_2_); 1710 (C=O); 1658 (C=N); 1620, 1579, 1523 (C=C). ^1^H NMR (500 MHz, DMSO-d_6_, ppm): *δ* 13.23 (1H, s, CONH); 12.06 (s, NH-isatin, 1H); 8.38 (1H, s, H-2); 8.24 (1H, d, *J* = 7.0 Hz, H-4); 7.73 (1H, s, H-4″); 7.57 (1H, d, *J* = 7.0 Hz, H-7); 7.45–7.51 (5H, m, H-5, H-6″, H-7″, H-3′, H-5′); 7.27–7.24 (2H, m, H-2′, H-6′); 7.09–7.08 (1H, m, H-6); 5.05 (2H, s, CH_2_). ^13^C NMR (125 MHz, DMSO-d_6_, ppm): *δ* 161.36; 141.12; 135.04; 132.78; 130.63; 129.80; 129.17; 128.05; 123.30; 122.09; 121.01; 120.48; 113.03; 112.31; 42.44. HR-MS (ESI) *m/z* 463.06921 [M + H]^+^. Anal. Calcd. for C_24_H_16_Cl_2_N_4_O_2_ (462.0650): C, 62.22; H, 3.48; N, 12.09. Found: C, 62.28; H, 3.45; N, 12.07.

#### 2.1.18. (Z)-N′-(7-Chloro-2-oxoindolin-3-ylidene)-1-(4-chlorobenzyl)-1H-indole-3-carbohydrazide (5d)

Yellow solid; yield: 60%. mp: 176.7–177.5 °C. *R_f_*=0.52 (DCM:MeOH = 14:1). IR (KBr, cm^–1^): 3398 (NH); 3167, 3147, 3109 (CH aren); 2927, 2860 (CH, CH_2_); 1724 (C=O); 1658 (C=N); 1662, 1546, 1521 (C=C). ^1^H NMR (500 MHz, DMSO-d_6_, ppm): *δ* 13.26, 11.49 (0.56H, 0.34H, 2s, CONH) ; 12.07 (1H, s, NH-isatin); 8.38 (1H, s, H-2); 8.23 (1H, d, *J* = 7.0 Hz, H-4); 7.75 (1H, d, *J* = 7.5 Hz, H-4″); 7.55 (1H, d, *J* = 7.0 Hz, H-7); 7.42–7.18 (8H, m, H-2′, H-6′, H-3′, H-5′, H-5, H-6, H-5″, H-6″); 5.35 (2H, s, CH_2_). ^13^C NMR (125 MHz, DMSO-d_6_, ppm): *δ* 162.05; 127.96; 136.70; 134.12; 132.99; 132.12; 129.06; 128.63; 125.40; 124.42; 123.69; 122.12. 121.93; 120.93; 119.66; 115.66; 113.07; 44.35. HR-MS (ESI) *m/z* 463.06927 [M + H]^+^. Anal. Calcd. for C_24_H_16_Cl_2_N_4_O_2_ (462.0650): C, 62.22; H, 3.48; N, 12.09. Found: C, 62.25; H, 3.42; N, 12.07.

#### 2.1.19. (Z)-N′-(5-Bromo-2-oxoindolin-3-ylidene)-1-(4-chlorobenzyl)- 1H-indole-3-carbohydrazide (5e)

Yellow solid; yield: 57%. mp: 176.7–177.5 °C. *R_f_*=0.50 (DCM:MeOH = 14:1). IR (KBr, cm^–1^): 3296 (NH); 3161, 3057 (CH aren); 2929, 2873 (CH, CH_2_); 1707 (C=O); 1666 (C=N); 1612, 1587, 1525 (C=C). ^1^H NMR (500 MHz, DMSO-d_6_, ppm): *δ* 13.20 (1H, s, CONH); 12.06 (1H, s, NH-isatin); 8.36 (1H, s, H-2); 8.24 (1H, d, *J* = 7.0 Hz, H-4); 7.80 (1H, s, H-4″); 7.56 (1H, d, *J* = 8.5 Hz, H-5″); 7.54 (1H, d, *J* = 8.0 Hz, H-7); 7.43–7.39 (4H, m, H-2′, H-3′, H-5′, H-6′); 7.27–7.22 (2H, m, H-5, H-6); 7.01 (1H, d, *J* = 8.5 Hz, H-6″); 5.02 (2H, s, CH_2_). ^13^C NMR (125 MHz, DMSO-d_6_, ppm): *δ* 161.18; 141.45; 136.73; 134.98; 133.41; 132.48; 129.77; 129.16; 126.35; 123.29; 122.41; 122.09; 121.00; 115.69; 113.01; 112.71; 42.41. HR-MS (ESI) *m/z* 507.01645 [M + H]^+^. Anal. Calcd. for C_24_H_16_BrClN_4_O_2_ (506.1045): C, 56.77; H, 3.18; N, 11.03. Found: C, 56.71; H, 3.19; N, 11.04.

#### 2.1.20. (Z)-1-(4-Chlorobenzyl)-N′-(5-methyl-2-oxoindolin-3-ylidene)-1H-indole-3-carbohydrazide (5f)

Yellow solid; yield: 62%. mp: 176.7–177.5 °C. *R_f_*=0.55 (DCM:MeOH = 14:1). IR (KBr, cm^–1^): 3147 (NH); 3109, 3055 (CH aren); 2920, 2866 (CH, CH_2_); 1724 (C=O); 1683 (C=N); 1624, 1579, 1533 (C=C). ^1^H NMR (500 MHz, DMSO-d_6_, ppm): *δ* 13.32 (1H, s, CONH); 12.07 (s, NH-isatin, 1H); 8.30 (1H, s, H-2); 8.25 (1H, d, *J* = 7.0 Hz, H-4); 7.57 (1H, d, *J* = 8.0 Hz, H-7); 7.49 (1H, s, H-4″); 7.42–7.39 (4H, m, H-2′, H-5′, H-3′, H-5′); 7.28–7.23 (2H, m, H-5, H-6); 7.16 (1H, d, *J* = 7.5 Hz, H-7″); 6.92 (1H, d, *J* = 7.5 Hz, H-6″); 5.05 (2H, s, CH_2_); 2.33 (3H, s, CH_3_). ^13^C NMR (125 MHz, DMSO-d_6_, ppm): *δ* 161.52; 140.26; 126.79; 135.33; 132.97; 132.70; 131.64; 129.75; 129.13; 123.22; 122.00; 121.26; 120.97; 120.24; 113.00; 110.54; 42.33; 21.01. HR-MS (ESI) *m/z* 443.12402 [M + H]^+^. Anal. Calcd. for C_25_H_19_ClN_4_O_2_ (442.1197): C, 67.80; H, 4.32; N, 12.65. Found: C, 67.85; H, 4.35; N, 12.61.

#### 2.1.21. (Z)-1-(4-Chlorobenzyl)-N′-(5-methoxy-2-oxoindolin-3- ylidene)-1H-indole-3-carbohydrazide (5g)

Yellow solid; yield: 69%. mp: 176.7–177.5 °C. *R_f_*=0.49 (DCM:MeOH = 14:1). IR (KBr, cm^–1^): 3221 (NH); 3109, 3015 (CH aren); 2931 (CH, CH_2_); 1714 (C=O); 1656 (C=N); 1519, 1487 (C=C). ^1^H NMR (500 MHz, DMSO-d_6_, ppm): *δ* 13.40 (1H, s, CONH); 12.07 (s, NH-isatin, 1H); 8.30 (1H, s, H-2); 8.23 (1H, d, *J* = 6.5 Hz, H-4); 7.60 (1H, s, H-4″); 7.56 (1H, d, *J* = 6.0 Hz, H-7); 7.44–7.48 (4H, m, H-2′, H-6′, H-3′, H-5′); 7.28–7.23 (3H, m, H-5, H-6, H-7″); 6.98 (1H, d, *J* = 9.0 Hz, H-6″); 5.02 (2H, s, CH_2_); 3.80 (3H, s, OCH_3_). ^13^C NMR (125 MHz, DMSO-d_6_, ppm): *δ* 161.56; 156.46; 136.82; 135.37; 132.69; 129.78; 129.16; 129.16; 123.25; 122.03; 121.16; 120.93; 117.07; 113.03; 111.67; 106.36; 56.19; 42.35. HR-MS (ESI) *m/z* 459.11905 [M + H]^+^. Anal. Calcd. for C_25_H_19_ClN_4_O_3_ (458.1146): C, 65.43; H, 4.17; N, 12.21. Found: C, 65.45; H, 4.13; N, 12.23.

### 2.2. Cytotoxicity assay

The cytotoxicity of the synthesised compounds was evaluated against three human cancer cell lines, including SW620 (colon cancer), PC3 (prostate cancer), and NCI-H23 (lung cancer). The cell lines were purchased from a Cancer Cell Bank at the Korea Research Institute of Bioscience and Biotechnology (KRIBB). The media, sera, and other reagents that were used for cell culture in this assay were obtained from GIBCO Co. Ltd. (Grand Island, NY). The cells were cultured in Dulbecco’s modified Eagle medium (DMEM) until confluence. The cells were then trypsinised and suspended at 3 × 10^4^ cells/ml of cell culture medium. On day 0, each well of the 96-well plates was seeded with 180 µl of cell suspension. The plates were then incubated in a 5% CO_2_ incubator at 37 °C for 24 h. Compounds were initially dissolved in dimethyl sulphoxide (DMSO) and diluted to appropriate concentrations by culture medium. Then, 20 µl of each compounds’ samples, which were prepared as described above, were added to each well of the 96-well plates, which had been seeded with cell suspension and incubated for 24-h, at various concentrations. The plates were further incubated for 48 h. Cytotoxicity of the compounds was measured by the colorimetric method, as described previously^22^ with slight modifications^23–26^. The IC_50_ values were calculated using a probit method and were averages of three independent determinations (SD ≤ 10%)^27^.

### 2.3. Caspase-3 activation assay

Caspase activity was measured by using caspase 3 assay kit according to the manufacturer’s instructions (Abcam, Cambridge, MA). U937 5 × 10^5^ cells/well seeding (2.5 × 10^5^ cells/ml, 2 ml/well, six well) were plated in six-well culture plates and allowed to grow for 24 h. The cells were treated with compounds or PAC-1 (50 µM) for 24 h, and then harvested. The harvested cells were washed twice with ice-cold PBS and treated with lysis buffer included in the kit. Cell lysate (100 µg/50 µl) was mixed with 50 µl of 2× reaction buffer and 5 µl of DEVD-*p*-NA substrate as the instruction of caspase-3 assay kit (Abcam, Cambridge, MA, cat. no. ab39401). Fluorescence was measured after one-hour incubation.

### 2.4. Cell cycle analysis

U937 5 × 10^5^ cells/well seeding (2.5 × 10^5^ cells/ml, 2 ml/well, six well) were plated in six-well culture plates and allowed to grow for 24 h. The cells were treated with compounds (50 µM) for 24 h, and then harvested. The harvested cells were washed twice with ice-cold PBS, fixed in 75% ice-cold ethanol, and stained with propidium iodide (PI) in the presence of RNase at room temperature for 30 min. The stained cells were analysed for DNA content using a FACScalibur flow cytometer (BD Biosciences, San Jose, CA) and the data were processed using Cell Quest Pro software (BD Biosciences, San Jose, CA).

### 2.5. Apoptosis assay

The Annexin V-FITC/PI dual staining assay was used to determine the percentage of apoptotic cells. U937 5 × 10^5^ cells/well seeding (2.5 × 10^5^ cells/ml, 2 ml/well, six well) were plated in six-well culture plates and allowed to grow for 24 h. The cells were treated with compounds (50 µM) for 24 h, and then harvested. The harvested cells were washed twice with ice-cold PBS and incubated in the dark at room temperature in 100 ml of 1× binding buffer containing 1 µl Annexin V-FITC and 12.5 ml PI. After 15 min incubation, cells were analysed for percentage undergoing apoptosis using a FACScalibur flow cytometer (BD Biosciences, San Jose, CA). The data were processed using Cell Quest Pro software (BD Biosciences, San Jose, CA).

### 2.6. Docking studies

Molecular docking was carried out using MOE 2009.10^28^ to study the interactions of synthesised compounds in catalytic domain site of caspase-6 (PDB entry 4FXO)^29^. The structure of reference compound PAC-1 was obtained from Pubchem databases and those of synthesised compounds were built using the Builder module of MOE software. The docking procedures were mainly based on those reported previously^14^^,^^25^, that include removing co-crystal ligand, adding hydrogen atoms, parameter setting, minimising structure, and fixing charge. For estimating the negative binding free energy profile of the complex (kcal/mol), the London scoring function was first used with force field (MMFF94x) refinement, and each binding pose was then minimised and rescored with the GBVI/WSA S scoring function implemented in MOE software^25^. It is emphasised that for docking caspases, water molecules must be conserved as they are an integral part of allosteric mechanisms^29^. For interaction visualisation, all the figures were built using the snapshots taken in BIOVIA Discovery Studio v.3.5 program.

## 3. Results and discussion

### 3.1. Chemistry

The target *N*′-substituted-1-(4-chlorobenzyl)-1*H*-indol-3-carbohydrazides (**4a–m**, **5a–g**) were synthesised as illustrated in Scheme 1. Starting from ethyl indol-3-carboxylate (**1**), a nucleophilic reaction with 4-chlorobenzyl bromide was affected using NaH as a base and a catalytic amount of KI. The reaction was best occurred in DMF. An acyl nucleophilic reaction in the second step between ethyl 1-(4-chlorobenzyl)-1*H*-indol-3-carboxylate (**2**) with hydrazine was smooth under refluxing conditions in EtOH to give a hydrazide (**3**) in a quantitative yield. Finally, the hydrazide (**3**) was condensed with respective benzaldehydes or isatins to afford the final series **4a–m**, **5a–g** in good overall yields.

The structures of the synthesised compounds were determined straightforwardly based on analysis of spectroscopic data, including IR, MS, ^1^H and ^13^C NMR. In all ^1^H NMR spectra of compounds in series **4a–m** and **5a–g**, there always appeared a singlet peak around 5.55 ppm, which was distinguished for two methylene protons from 1-(4-chlorobenzyl)indole part. In the ^13^C NMR spectra of compounds in series **4a–m**, a downfield peak around 162 ppm was attributable for a carbonyl carbon of the acetohydrazide functionality. In the ^13^C NMR spectra of compounds in series **5a–g**, one additional more downfield carbon around 168 ppm was characteristic for the carbonyl carbon of the 2-oxoindoline system.

### 3.2. Bioactivity

The synthesised compounds (**4a–m**, **5a–g**) were first evaluated for their cytotoxicity using an SRB method as described previously^20^ with slight modifications as previously reported^23–26^. Three human cancer cell lines, including SW620 (a colon cancer), PC3 (a prostate cancer), and NCI-H23 (a lung cancer), were used in the screening. The results, which were averages of the triplicate measurements, are summarised in Table 1. 5-Fluorouracil, a currently used anticancer drug, was employed as a positive control. Also, PAC-1 and oncrasin-1 were included in the evaluation for comparison purpose. As can be seen from Table 1, all compounds in series **4a–m** and **5a–g** were active against three human cancer cell lines tested. In series **4a–m**, compound **4a** (IC_50_, 1.67–2.09 µM) displayed slightly more potent cytotoxicity in comparison to PAC-1 (IC_50_, 4.23–5.11 µM) and oncrasin-1 (IC_50_, 3.11–3.32 µM). This compound was approximately 4- to 8-fold more potent than 5-FU (IC_50_, 8.84–13.61 µM) in term of cytotoxicity. Substitution of Cl at the meta position on the phenyl ring resulted in compound **4c** with decreased cytotoxicity (IC_50_, 4.70–5.25 µM). However, ortho substitution of Cl substantially enhanced cytotoxicity (**4b**, IC_50_, 0.12–0.16 µM). Especially, compound **4d** with Cl substituted at para position exhibited extremely potent cytotoxicity with IC_50_ of 0.001–0.002 µM in all three human cancer cell lines. Compound **4d** was virtually the most potent one in the series. Surprisingly, addition of an ortho chloro substituent led to the loss of antiproliferative activity (**4e**, IC_50_>10 µM). Compound **4f** with 4-NO_2_ substituent also displayed very potent cytotoxicity (IC_50_, 0.005–0.011 µM). Thus, it seemed that substitution of electron withdrawing groups, e.g. Cl or NO_2_ at para-position greatly enhanced cytotoxicity. In contrast, substitution of electron releasing groups, e.g. –OCH_3_ (compound **4k**) or –N(CH_3_)_2_ (compound **4l**) at para-position did not improve, even led to the loss of cytotoxicity. Compound **4m** with multiple methoxy substituents was also inactive. Finally, it was very interesting to note that in series **4a–m**, three compounds (**4g–i**) with 2-OH substituent all exhibited potent cytotoxicity in three human cancer cell lines assayed with IC_50_ values in the range of 0.56–0.83 µM. The cytotoxic potency of compounds **4g–i** was approximately 6- to 10-fold higher than that of PAC-1. From the results obtained, it was clear that for compounds in series **4a–m**, 2-Cl or 2-OH substituents were favourable for cytotoxicity. Compounds with 4-Cl and 4-NO_2_ substituents, and likely other electron withdrawing groups, were the most potent ones.

**Table 1.** Cytotoxicity of the selected compounds against some human cancer cell lines. 
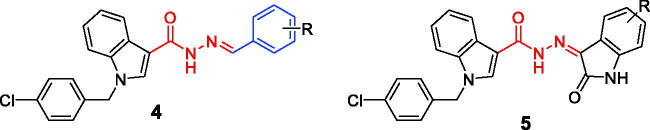

Cpd code	R	MW	Cytotoxicity (IC_50_^a^, µM)/cell lines^b^		
			SW620	PC3	NCI-H23
**4a**	H	387.87	2.09 ± 0.138	2.97 ± 0.017	1.67 ± 0.056
**4b**	2-Cl	422.31	0.16 ± 0.002	0.16 ± 0.006	0.12 ± 0.002
**4c**	3-Cl	422.31	5.25 ± 0.059	4.70 ± 0.145	4.81 ± 0.098
**4d**	4-Cl	422.31	0.002 ± 0.000	0.002 ± 0.000	0.001 ± 0.000
**4e**	2,6-Cl_2_	456.75	>10	>10	>10
**4f**	4-NO_2_	432.86	0.011 ± 0.001	0.010 ± 0.000	0.005 ± 0.001
**4g**	2-OH	403.87	0.62 ± 0.003	0.58 ± 0.014	0.69 ± 0.017
**4h**	2-OH-3-allyl	443.93	0.58 ± 0.000	0.61 ± 0.003	0.56 ± 0.000
**4i**	2-OH-4-OCH_3_	433.89	0.80 ± 0.042	0.83 ± 0.020	0.62 ± 0.029
**4j**	3-OH-4-OCH_3_	433.89	5.00 ± 0.065	7.31 ± 0.423	6.60 ± 0.407
**4k**	4-OCH_3_	417.89	2.53 ± 0.055	3.29 ± 0.023	2.45 ± 0.028
**4l**	4-N(CH_3_)_2_	430.94	>10	>10	>10
**4m**	3,4,5-(OCH_3_)_3_	477.94	>10	>10	>10
**5a**	H	428.88	1.96 ± 0.055	1.54 ± 0.020	1.71 ± 0.044
**5b**	5-F	446.87	2.28 ± 0.084	1.58 ± 0.012	1.71 ± 0.040
**5c**	5-Cl	463.32	4.33 ± 0.223	4.46 ± 0.039	3.83 ± 0.247
**5d**	7-Cl	463.32	4.49 ± 0.203	3.74 ± 0.142	2.68 ± 0.225
**5e**	5-Br	507.77	1.70 ± 0.051	1.61 ± 0.022	1.17 ± 0.076
**5f**	5-CH_3_	442.90	7.56 ± 0.200	4.71 ± 0.006	5.13 ± 0.382
**5g**	5-OCH_3_	458.90	5.27 ± 0.192	3.79 ± 0.180	3.86 ± 0.076
**5-FU**^c^		130.08	8.84 ± 1.92	13.61 ± 0.46	13.45 ± 3.92
**PAC-1**^d^		392.49	4.57 ± 0.17	4.23 ± 0.45	5.11 ± 0.07
**Oncrasin-1**^e^		269.06	3.17 ± 0.24	3.11 ± 0.28	3.32 ± 0.31

^a^The concentration (µM) of compounds that produces a 50% reduction in cell growth, the numbers represent the averaged results from triplicate experiments with deviation of less than 10%.

^b^Cell lines: SW620, colon cancer; PC3, prostate cancer; NCI-H23, lung cancer.

^c^5-FU: 5-fluorouracil, a positive control.

^d^PAC-1: a positive control.

^e^Oncrasin-1: a positive control.

Seven compounds in series **5a–g** bearing 2-oxoindoline ring were less potent than compounds in series **4a–m**. Only three compounds, including **5a**, **5b**, and **5e**, were cytotoxically equipotent in comparison to compound **4a**. Other compounds showed relatively higher IC_50_ values (up to 7.56 µM).

**Figure 2.**
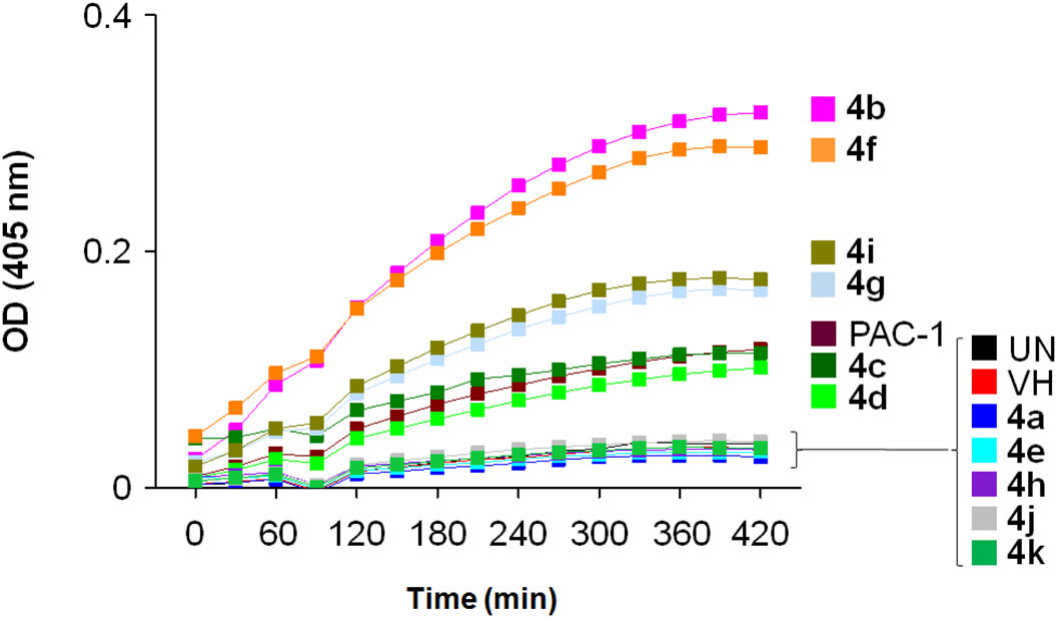
Caspase-3 activation activity of compounds **4a–k**. U937 5 × 10^5^ cells/well seeding (2.5 × 10^5^ cells/ml, 2 ml/well, six well) were treated with PAC-1 or compounds (50 µM) for 24 h. Cell lysate was used to detect caspase-3 activation by caspase-3 assay kit. UN: untreated; VH: vehicle (DMSO 0.05%).

**Figure 3.**
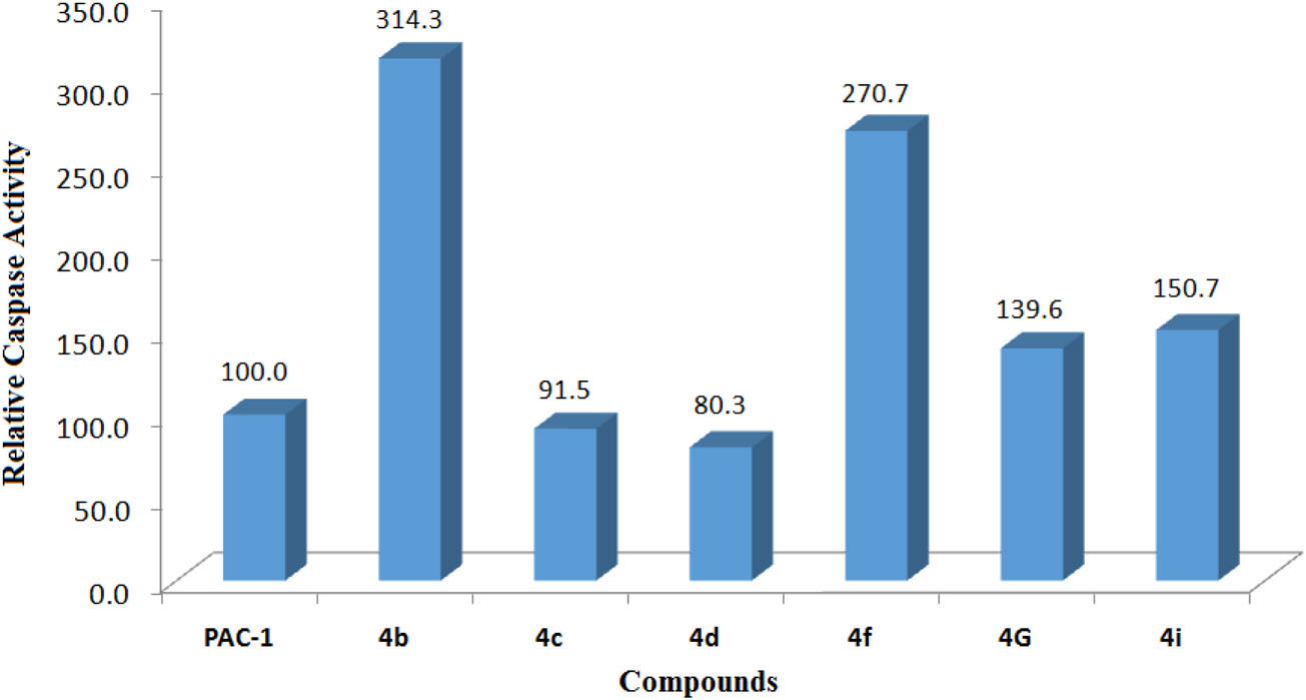
Relative caspase activation activity of some compounds in comparison to PAC-1. Compounds were tested at 50 µM.

To get a preliminary insight into possible mechanisms of cytotoxicity of the synthesised compounds, compounds **4a–k** were selected for further evaluation in caspase activation assay. U937 5 × 10^5^ cells/well seeding (2.5 × 10^5^ cells/ml, 2 ml/well, six well) were allowed to grow for 24 h, then treated with compounds or PAC-1 (50 µM) for 24 h, and harvested. The cells were lysed and caspase activity was measured using a caspase 3 assay kit according to the manufacturer’s instructions (Abcam, Cambridge, MA). The results shown in Figures 2 and 3 demonstrate that six compounds, including **4b–d**, **4f**, **4i**, and **4g**, clearly activated caspase activity. Overall, the caspase activation activity was found to be relatively correlated well with cytotoxicity in SRB assay. For example, compounds **4g** and **4i**, which were about 6- to 10-fold more potent than PAC-1 in term of cytotoxicity, showed relative activation of caspase activity by 139.6 and 150.7%, in comparison to that of PAC-1. Compound **4b**, which exhibited approximately 40-fold more potent cytotoxicity than PAC-1, was found to activate caspase activity by 314.3% higher in relation to PAC-1. Also, compound **4f** activated caspase activity by 270.7% relative to PAC-1. In SRB assay, this compound was found to be approximately 415- up to 1022-fold more cytotoxic than PAC-1. However, some exceptions in correlation between caspase activation activity and cytotoxicity were also noted. Compound **4h**, which was similarly cytotoxic as compounds **4g** and **4i**, did not activate caspase activity. Compound **4d**, which was virtually the most potent compound in terms of cytotoxicity, was found to activate the caspase activity by only 80.3% in relation to PAC-1. Since the assay performed in this study mainly measured caspase 3 activity, it is likely that these compounds might activate other caspase isoform. It is also possible that the compounds might act on other targets and additional investigation should be furthered to fully elucidate the cytotoxic mechanism of these acetohydrazides.

**Figure 4.**
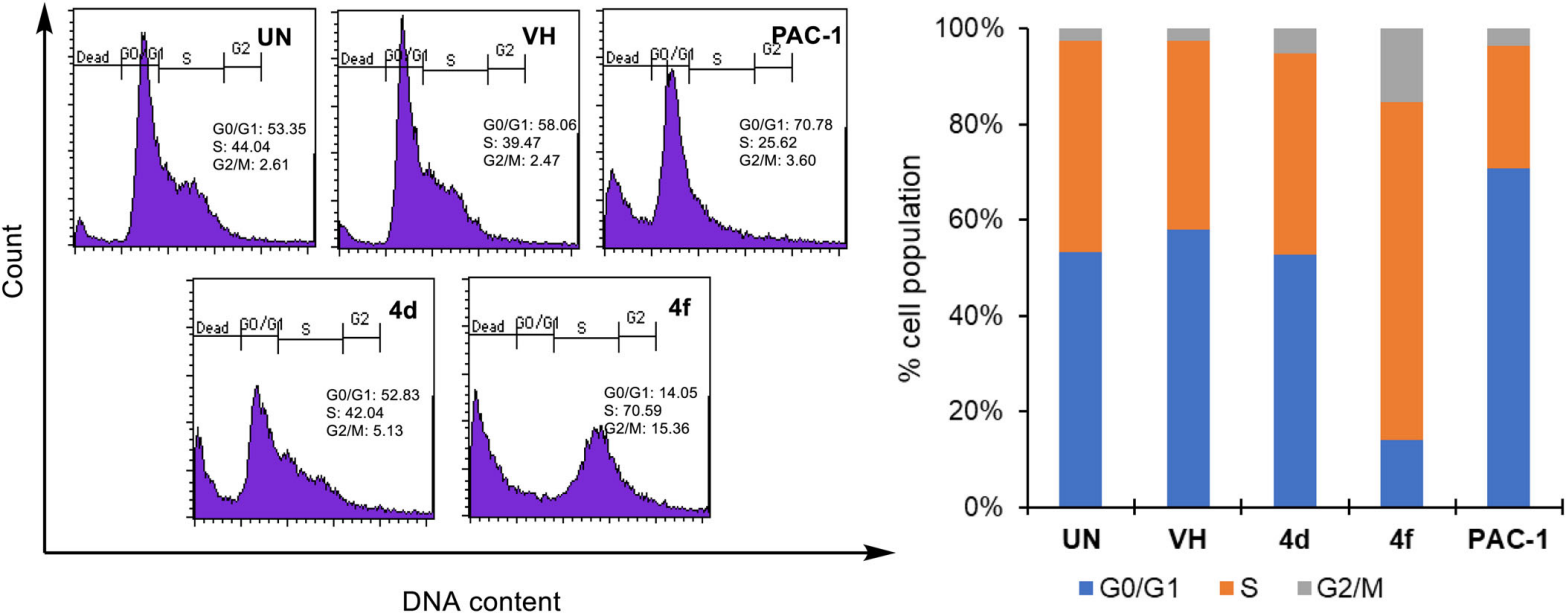
Cell cycle analysis of some compounds. U937 5 × 10^5^ cells/well seeding (2.5 × 10^5^ cells/ml, 2 ml/well, six well) were treated with compounds (50 µM) for 24 h. The harvested cells were stained with propidium iodide (PI) in the presence of RNase and then were analysed for DNA content. UN: untreated; VH: vehicle (DMSO. 0.05%). Data were represented as histograms (left) and bar graphs (right).

**Figure 5.**
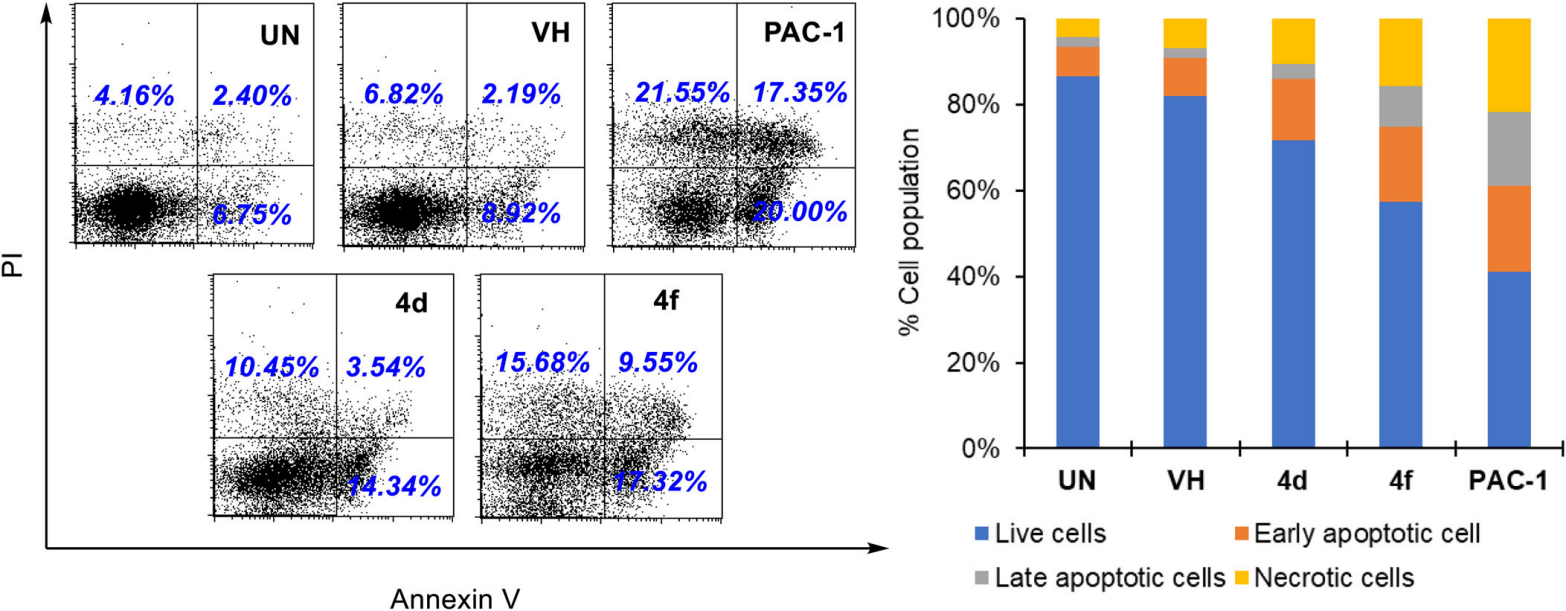
Apoptosis (Annexin V/PI) analysis of some compounds. U937 5 × 10^5^ cells/well seeding (2.5 × 10^5^ cells/ml, 2 ml/well, six well) were treated with compounds (50 µM) for 24 h. The harvested cells were incubated with Annexin V-FITC and PI. UN: untreated; VH: vehicle (DMSO. 0.05%). Area 1 = PI positive population, area 2: Annexin V-positive population. Data were represented as histograms (left) and bar graphs (right).

**Figure 6.**
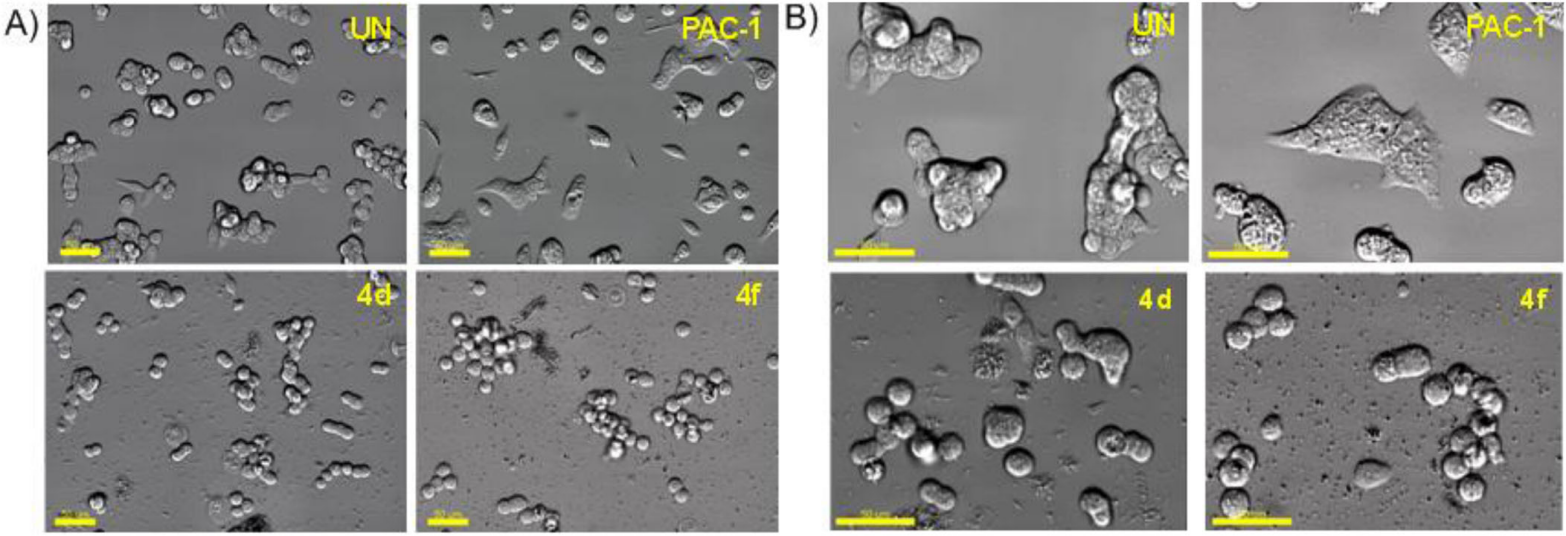
Morphology changes of cells treated with representative compounds **4d** and **4f**. U937 5 × 10^5^ cells/well seeding (2.5 × 10^5^ cells/ml, 2 ml/well, six well) were incubated for 24 h, then compounds **4a**, **4f** or PAC-1 (50 μM) were added and incubated further for 24 h. The cells were then photographed using an Imaging Device: Zeiss, Celldiscoverer7 with ×40 lens, scale bar: 20 µm (A) and 50 µm (B). UN: untreated.

Next, we decided to select two compounds **4d** and **4f** to analyse for their effects on cell cycle and apoptosis. U937 cells were treated with compounds or PAC-1 for 24 h, then flow cytometry was used to analyse the DNA content after cell lysis. It could be seen that both compounds, especially compound **4f**, caused substantial population of cells accumulated at S and G2/M phases (Figure 4). At this concentration, two compounds also caused a large population of cell death (19.29% for **4d** and 35.36 for **4f**). In Annexin V-FITC/PI dual staining assay, compounds **4d** and **4f** were found to substantially cause both early and late cellular apoptosis (Figure 5). These results demonstrate both compounds **4d** and **4f** were strong apoptotic inducers, similar to PAC-1. In line with their effects on the cell cycle and apoptosis, compounds **4d** and **4f** caused clear cellular morphological changes, as shown in Figure 6.

### 3.3. Molecular docking studies

As can be observed in Figure 3, 4b, **4f**, **4g**, and **4i** were identified as potential procaspase-activating compounds, with activity up to 150–300 times higher than PAC-1. They also showed good cytotoxicity against three cancer cell lines (SW620, PC3, and NCI-H23), especially **4f** with IC_50_ of 0.005–0.011 µM which is significantly higher than that of PAC-1. These compounds are therefore considered as potential hit compounds and needed to be explored for their structure–activity relationships that are useful for further hit-to-lead optimisation. To do so, these four derivatives were docked into the active site of procaspase enzymes taking into account the studies reported elsewhere.

**Figure 7.**
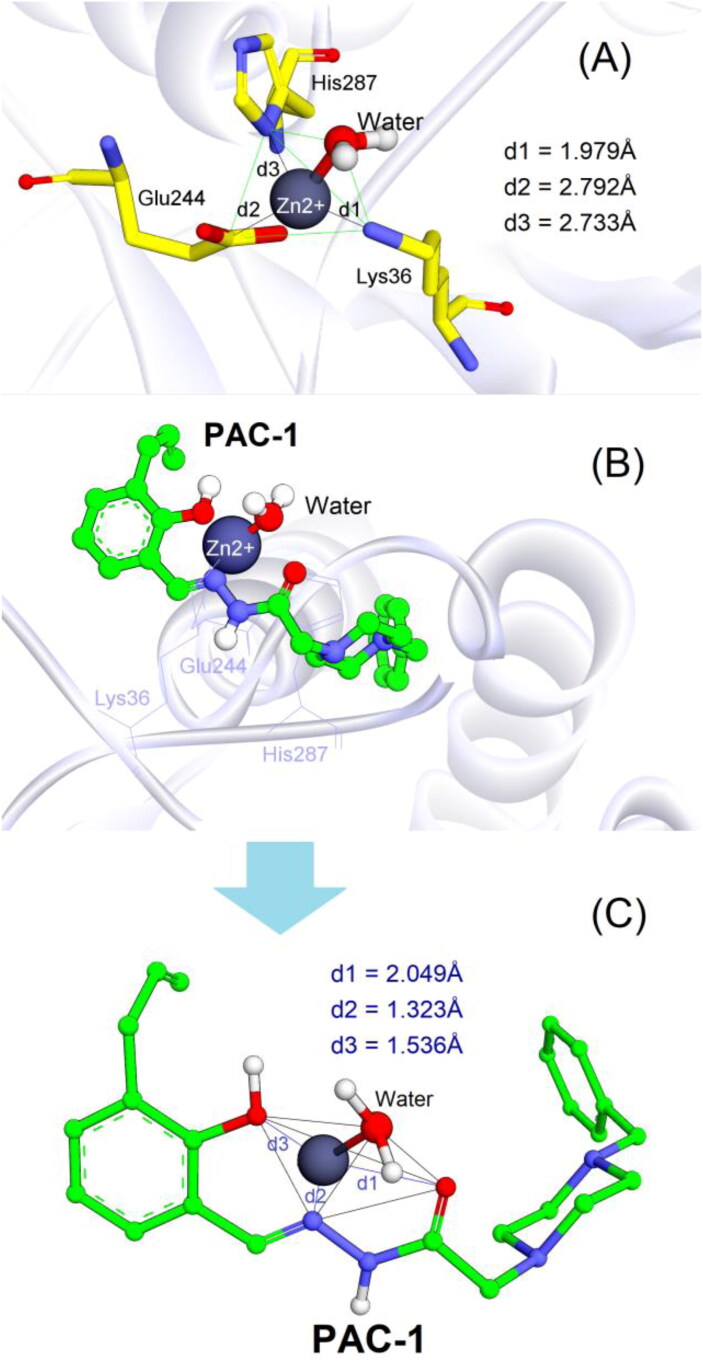
(A) Tetrahedral geometry of Lys36, Glu244, His287 and water molecule with zinc ion; (B) 3D structure of docked complex between PAC-1 and zinc ion in the allosteric binding site of caspase-6 (PDB ID: 4FXO); (C) tetrahedral geometry of the corresponding complex.

It is well known that caspases are cysteine-related proteases, which are originally synthesised as inactive dimers or zymogen procaspases and subsequently activated during apoptotic processes^30^. The procaspase activating derivatives, e.g. B-PAC-1, WF-208, and S-PAC-1^13^^,^^31^^,^^32^, convert inactive executioner procaspases (caspase-3, -6, and -7) into their active cleaved forms by chelation of labile zinc ion which is a key physiological inhibitor of caspases. In the previous studies, a large number of protein data of caspases have been reviewed and the crystallographical structure of caspase-6 (a homologous structure of caspase-3) in complex with zinc ion reported by Delgado and Hardy (PDB ID: 4FXO) was identified as suitable protein model to investigate the ability of small molecules to chelate the intracellular zinc to activate caspases^14^^,^^16^^,^^29^. In the allosteric site of this crystal caspase structure^29^, zinc ion exerted its inhibitory role by establishing a tetrahedral geometry with Lys36, Glu244, His287, and water (Figure 7(A)).

The PAC-1 compound was first docked into the allosteric site of zinc-bound caspase-6. As shown in Figure 7(B,C), the results clearly indicated that the metal chelating properties of ortho-hydroxy N-acylhydrazone is necessary for zinc binding and procaspase activation. By penetrating into the allosteric pocket of caspase-6, PAC-1 was able to form stable complex with zinc ion and one water molecule, showing similar coordination geometry formed between procaspase and zinc^29^. The binding free energy (S) estimated by GBVI/WSA force field-based scoring function was –5.03 kcal/mol, suggesting appropriate stability of PAC-1 in the complex.

**Figure 8.**
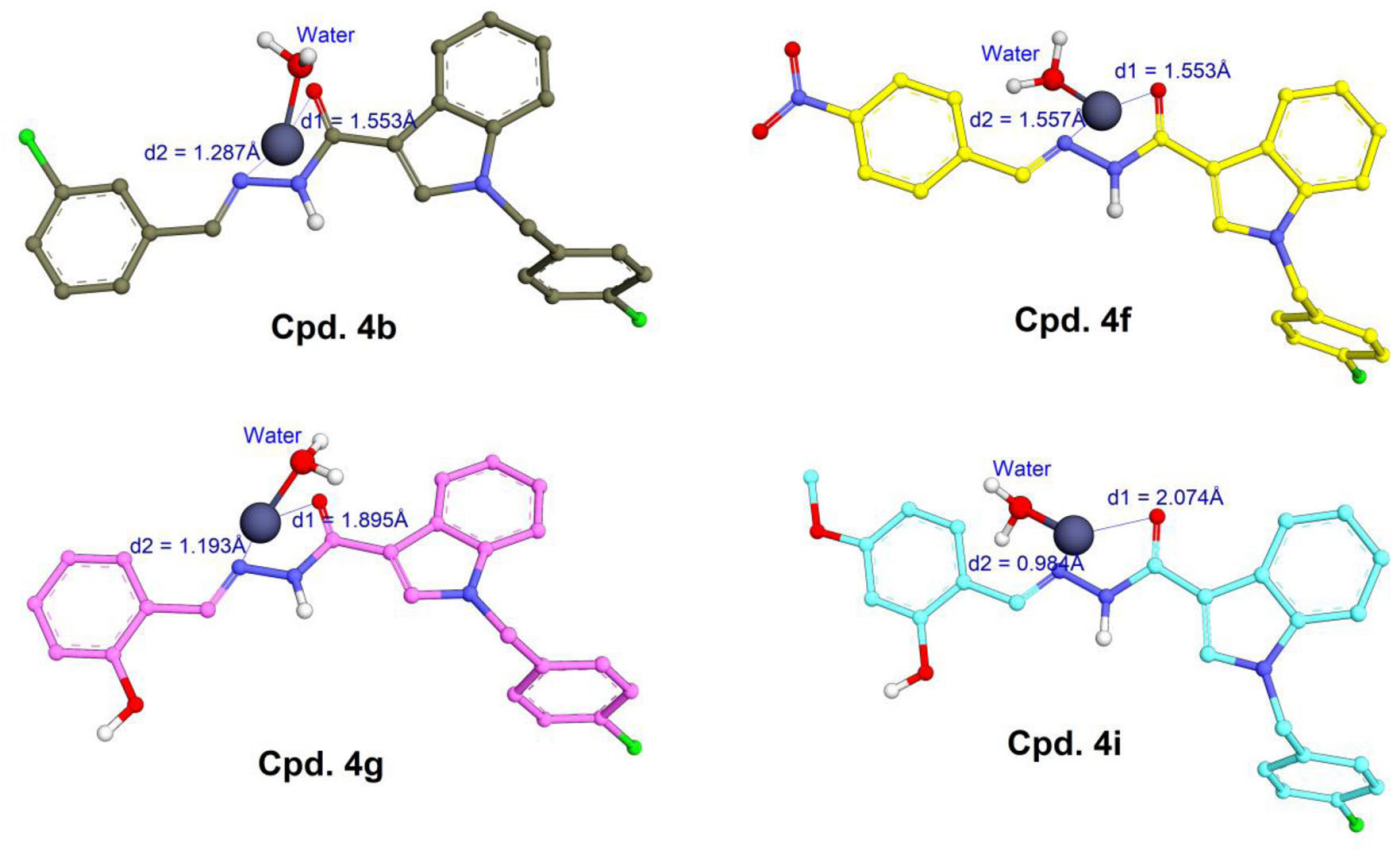
Metal complex and geometries between **4b**, **4f**, **4g**, and **4i** with zinc (grey spheres) extracted from locked caspase-6 (PDB ID: 4FXO).

Four derivatives **4b**, **4f**, **4g**, and **4i** were subsequently docked with the zinc ion extracted from the allosteric binding site of procaspase enzyme. Compared to PAC-1, these compounds exhibited a slight difference in the zinc chelating mode (Figure 8). They were able to form bidentate chelations between =N– and =O groups of the N-acyl hydrazine moiety towards zinc ion. The similarly close distances (1–2 Å) between these atoms and zinc plays an important role in zinc-binding motif of the ligands. Despite the fact that compounds **4g** and **4i** owned *ortho*-hydroxy N-acyl hydrazine structures similar to PAC-1 the 2-hydroxyl groups could not participate in chelation with zinc like PAC-1. Interestingly, indole, N-acyl hydrazine, and phenyl moieties did not show the flexibility that PAC-1 did (see Figure 7(C)); instead, they formed a conjugated system and are almost aligned with zinc ion. Among four ligands, **4b** and **4f** exhibited quite low binding energies (–7.15 and –6.90 kcal/mol), meanwhile **4g** and **4i** showed *S* values of –5.15 and –5.36 kcal/mol, suggesting a higher affinity towards zinc compared to PAC-1.

## 4. Conclusions

In conclusion, we have designed and synthesised two series of (*E*)-*N′-*benzylidene-carbohydrazides (**4a–m**) and *(Z)-N*′-(2-oxoindolin-3-ylidene)carbohydrazides (**5a–g**) incorporating 1-(4-chlorobenzyl)-1*H*-indole core. The results from bioevaluation demonstrated that the compounds, especially compounds in series **4a–m**, exhibited potent cytotoxicity against three human cancer cell lines (SW620, colon cancer; PC-3, prostate cancer; NCI-H23, lung cancer). Analysis of structure–activity relationships revealed that among series **4a–m**, compounds with 2-OH substituent (**4g–i**) exhibited very strong cytotoxicity in three human cancer cell lines assayed with IC_50_ values in the range of 0.56–0.83 µM. Compound **4a** with a 2-Cl substituent was even more potent with IC_50_ values of 0.12–0.16 µM. In particular, two compounds **4d** and **4f**, bearing 4-Cl and 4-NO_2_ substituents, respectively, were the most potent in terms of cytotoxicity with IC_50_ values of 0.011–0.001 µM. Compounds **4b** and **4f** strongly activated caspase activity by 314.3 and 270.7% relative to PAC-1. Annexin V-FITC/PI dual staining assay revealed two compounds **4d** and **4f** as strong apoptotic inducers, similar to PAC-1. In flow cytometry analysis, compound **4f** was found to arrest U937 at S and G2/M phases. Subsequent docking simulations for **4b**, **4f**, **4g**, and **4i** also revealed that these compounds could form stable chelations with zinc ion that is a key physiological inhibitor of caspases. However, the geometries between the *N*-acylhydrazone moiety and zinc were significantly different from that of PAC-1. The conjugation effect between indole, *N*-acylhydrazone, and phenyl moieties played the central role for the penetration of the allosteric site of procaspase and zinc binding mode of the ligands. Thus, this investigation has demonstrated the potential of these simple acetohydrazides, especially compounds **4b**, **4d**, and **4f**, as anticancer agents.

## Supplementary Material

Supplemental Material
