# Supplementary material for: Design, synthesis, and evaluation of novel N'-substituted-1-(4-chlorobenzyl)-1H-indol-3-carbohydrazides as antitumor agents
Source: J Enzyme Inhib Med Chem. 2020 Sep 28;35(1):1854–65. doi: 10.1080/14756366.2020.1816997 (PMC7534272; doi:10.1080/14756366.2020.1816997)
Supplement: Supplemental Material [file IENZ_A_1816997_SM6267.pdf]

SUPPLEMENTAL MATERIALS

for

Design, Synthesis and Evaluation of Novel *N'*-substituted-1-(4-chlorobenzyl)-1*H*-indol-3-carbohydrazides as Antitumor Agents

Le Cong Huan,[a,d] Duong Tien Anh,[a] Pham-The Hai,[a] Lai Duc Anh,[a] Eun Jae Park,[b] A Young Ji,[b] Jong Soon Kang,[c] Do Thi Mai Dung,[a] Dao Thi Kim Oanh,[a] Truong Thanh Tung,[e,f] Dinh Thi Thanh Hai,[*,a] Sang-Bae Han,[*,b] Nguyen-Hai Nam[*,a]

[a]*Hanoi University of Pharmacy, 13-15 Le Thanh Tong, Hanoi, Vietnam*
[b]*College of Pharmacy, Chungbuk National University, 194-31, Osongsaengmyung-1, Heungdeok, Cheongju, Chungbuk, 28160, Republic of Korea*
[c]*Bio-Evaluation Center, Korea Research Institute of Bioscience and Biotechnology, Cheongju, Chungbuk, Republic of Korea*
[d]*Thai Binh University of Medicine and Pharmacy, Thai Binh City, Vietnam*
[e]*Faculty of Pharmacy, PHENIKAA University, Hanoi, 12116, Vietnam*
[f]*PHENIKAA Institute for Advanced Study (PIAS), PHENIKAA University, Hanoi, 12116, Vietnam*
Keywords: Acylhydrazones, acetohydrazides, cytotoxicity, caspase activation.

***Corresponding authors:** Tel.: +84-4-39330531; Fax: +84-4-39332332; Emails: shan@cbnu.ac.kr (S.B. Han); namnh@hup.edu.vn (N.H. Nam).

ALL ^{1}H & ^{13}C NMR SPECTRA OF THE COMPOUNDS

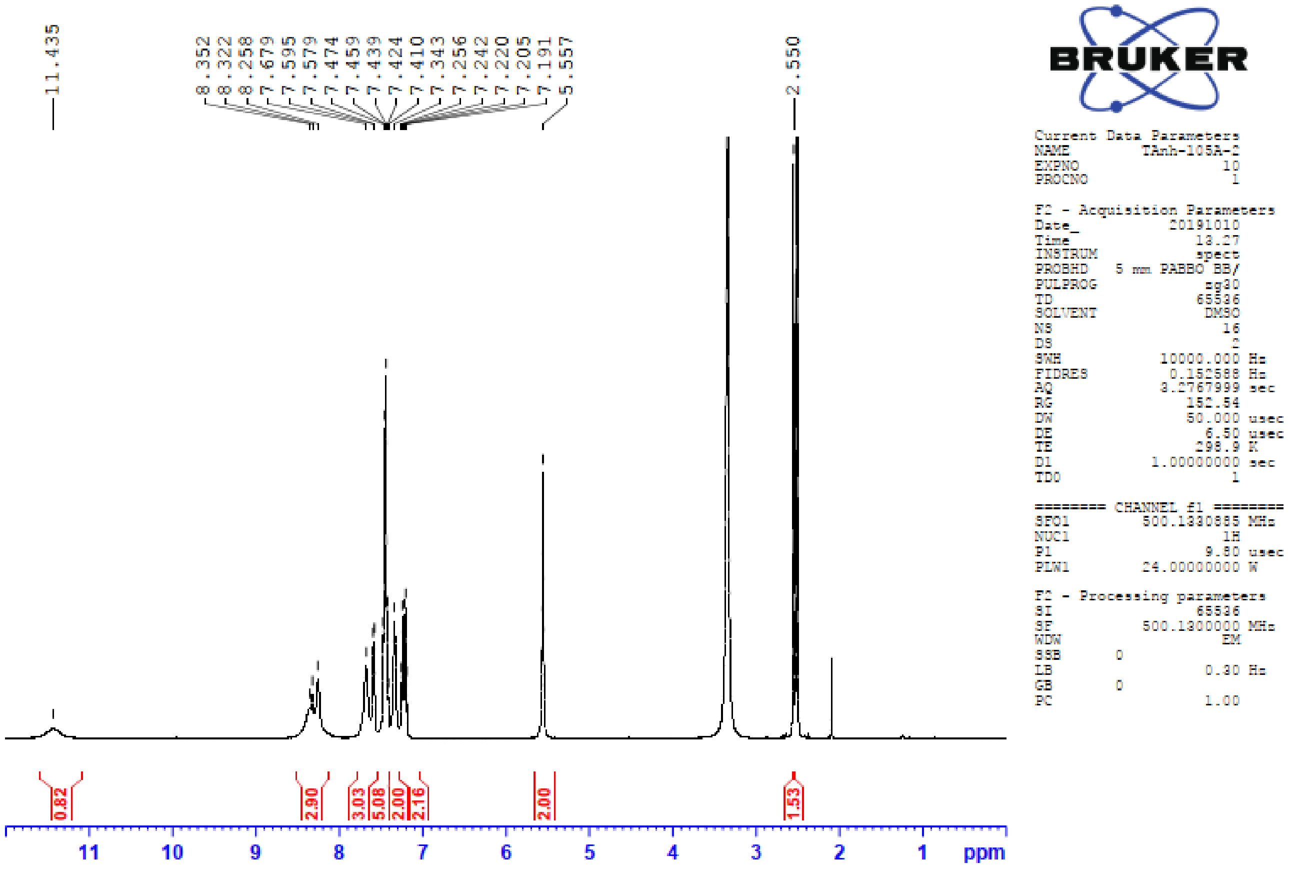

^{1}H-NMR of compound 4a

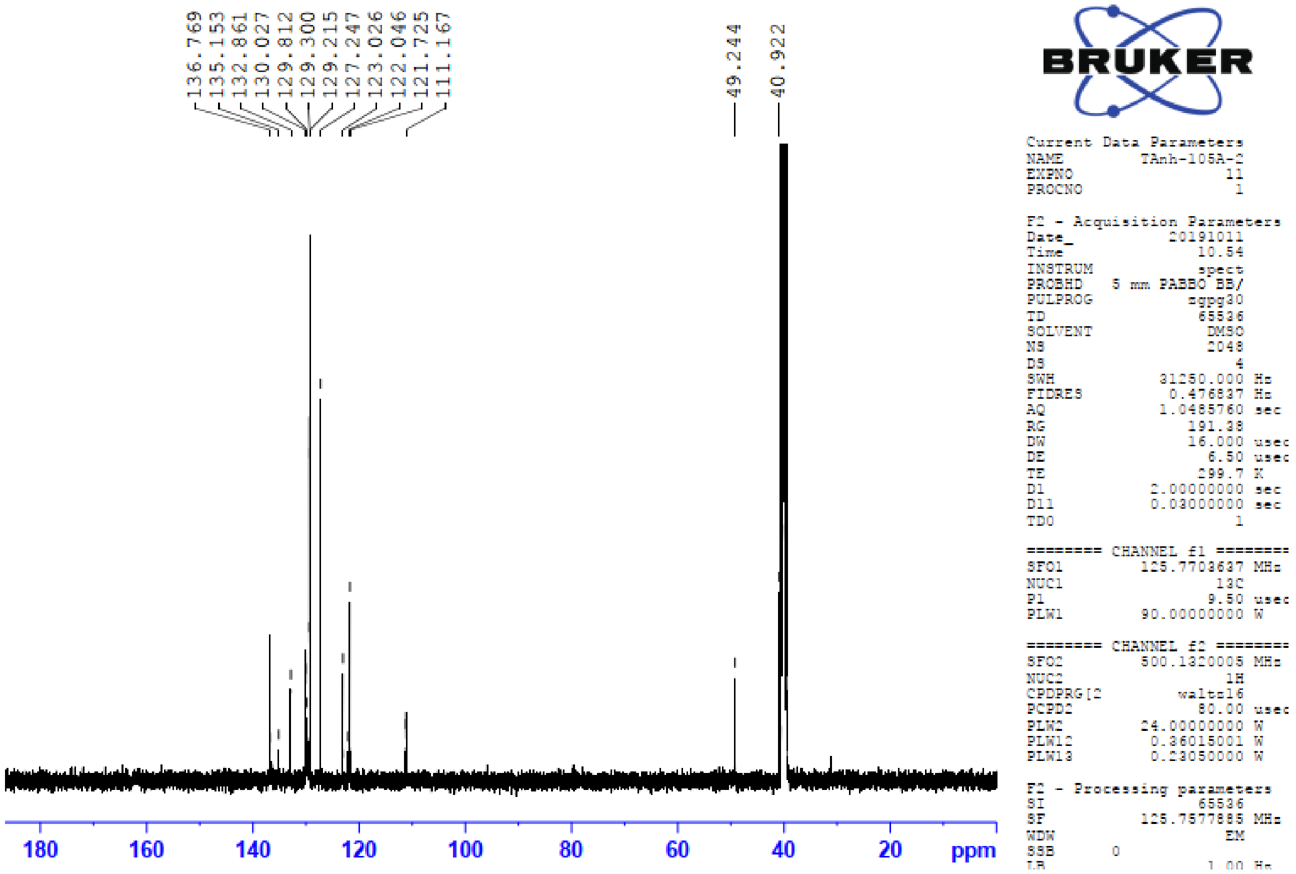

^{13}C-NMR of compound 4a

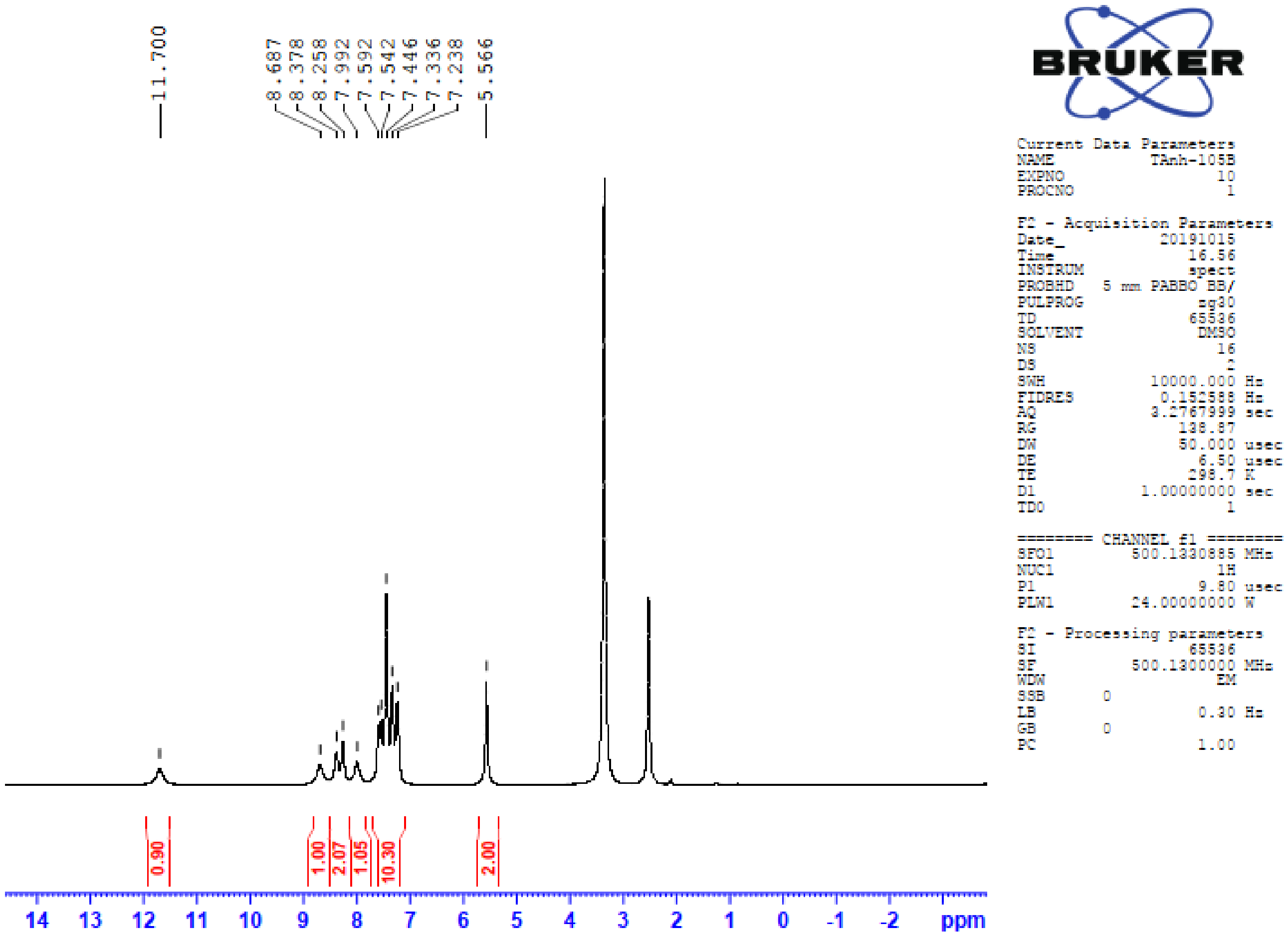

^{1}H-NMR of compound 4b

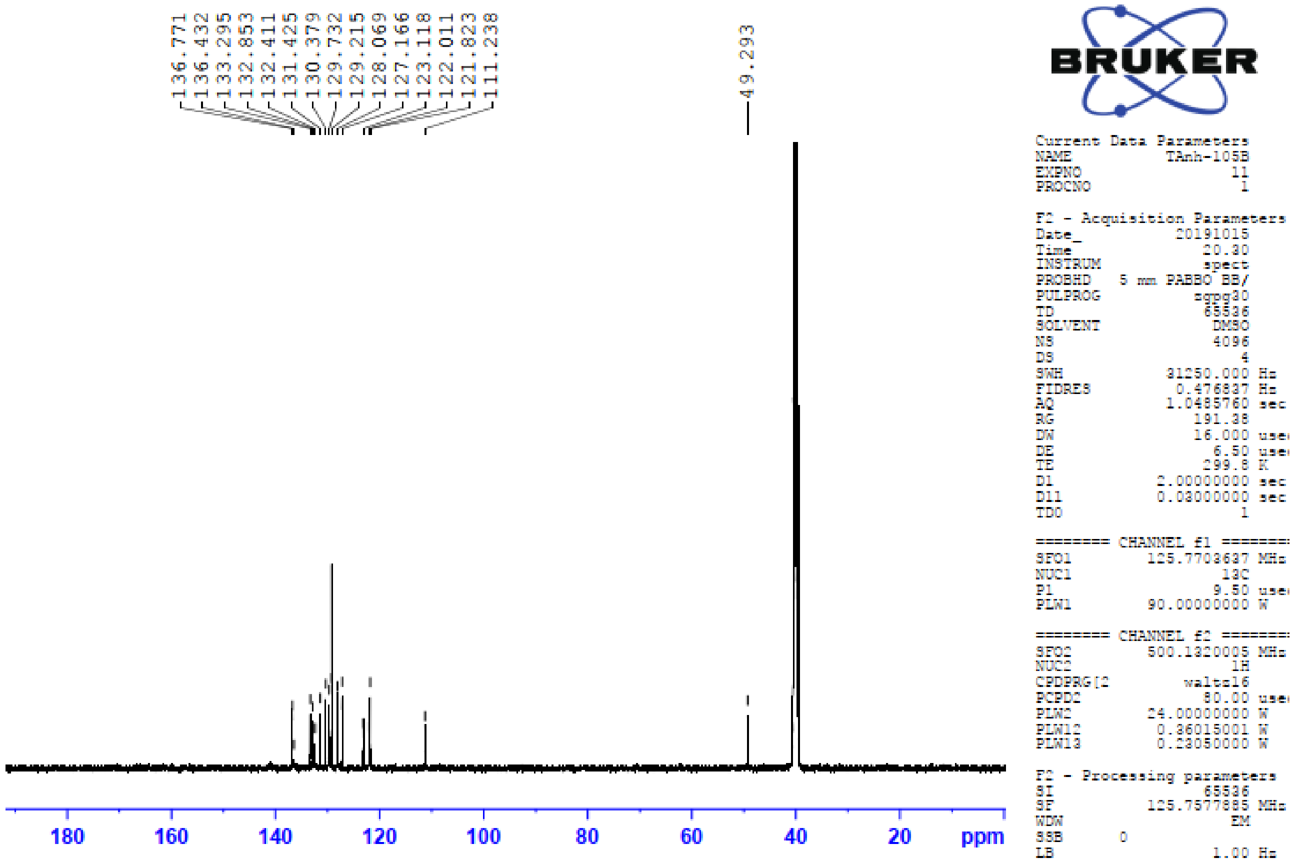

^{13}C-NMR of compound 4b

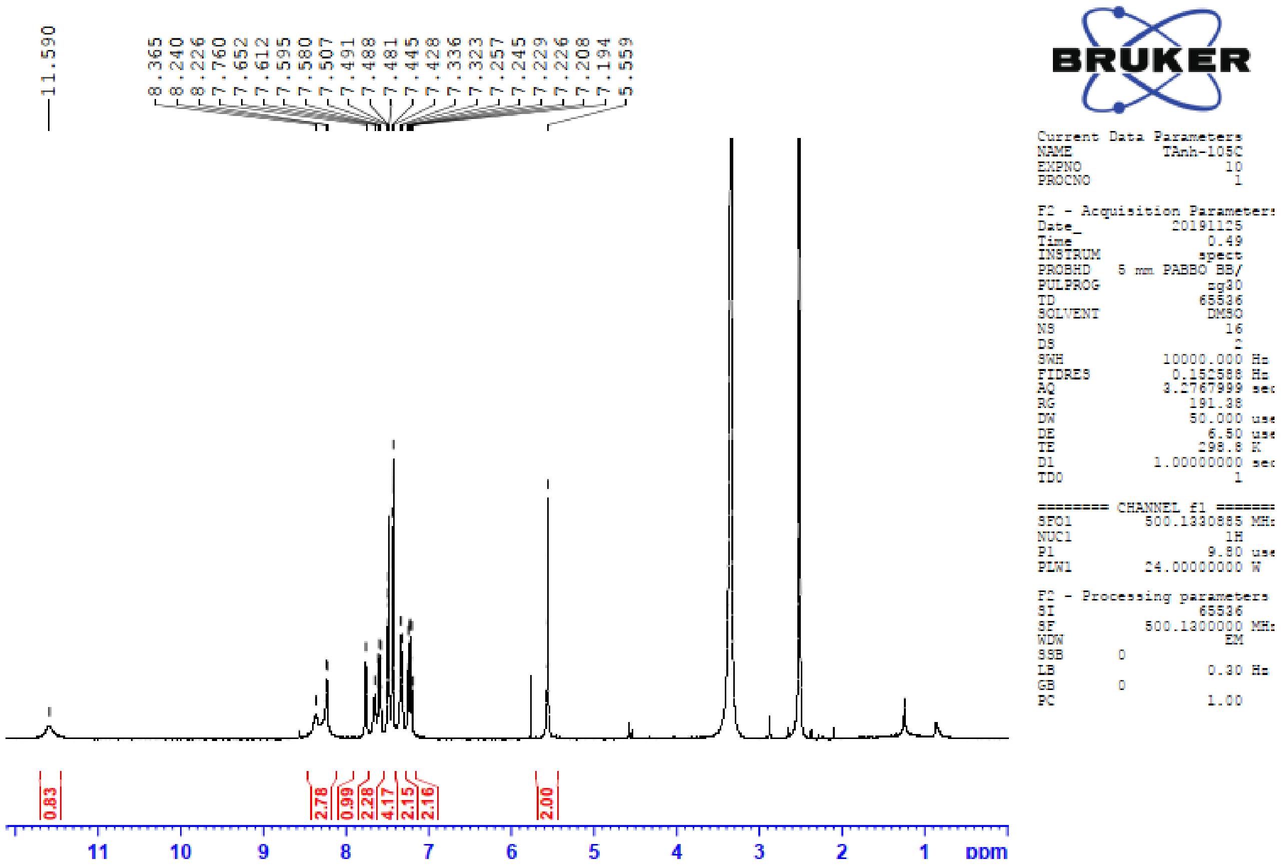

^{1}H-NMR of compound 4c

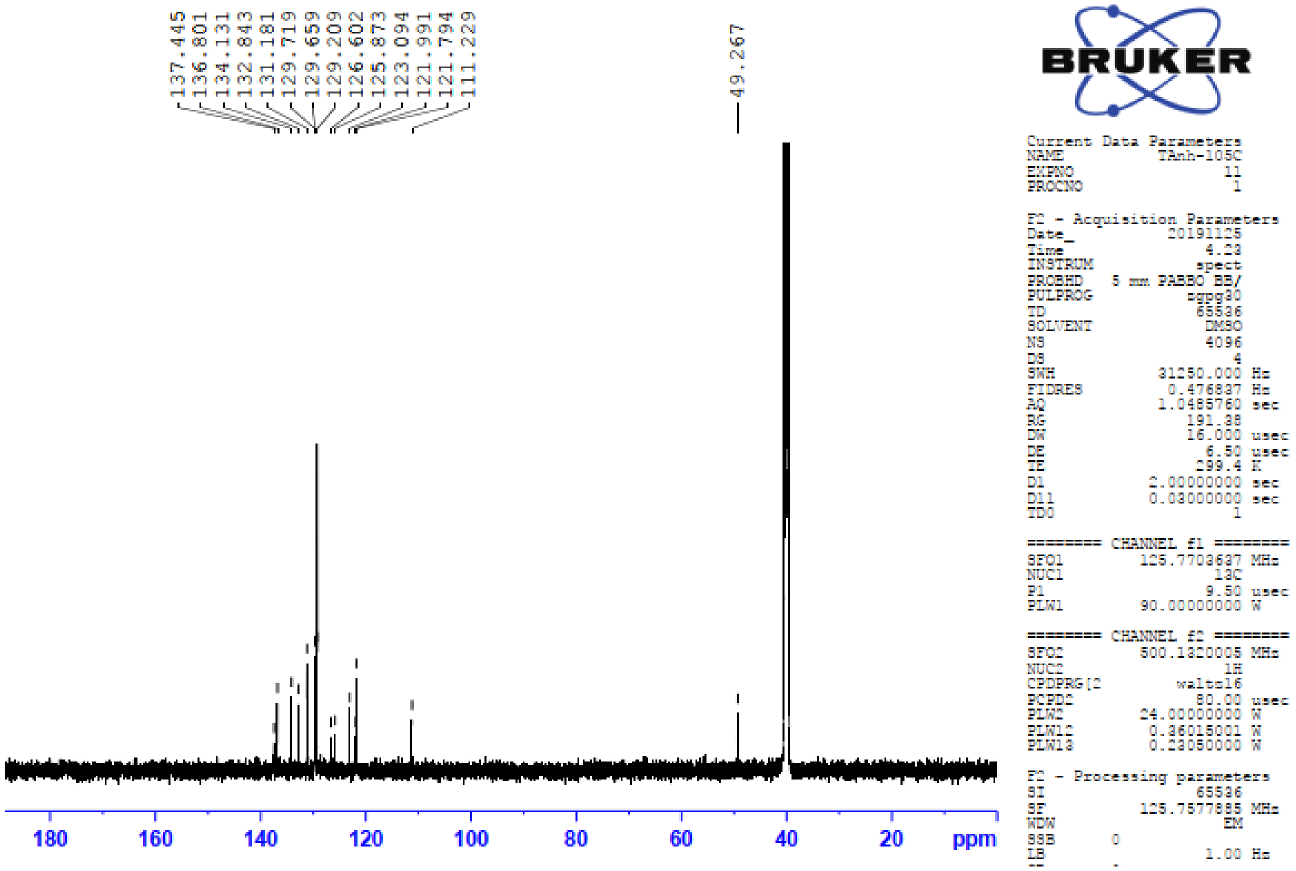

^{13}C-NMR of compound 4c

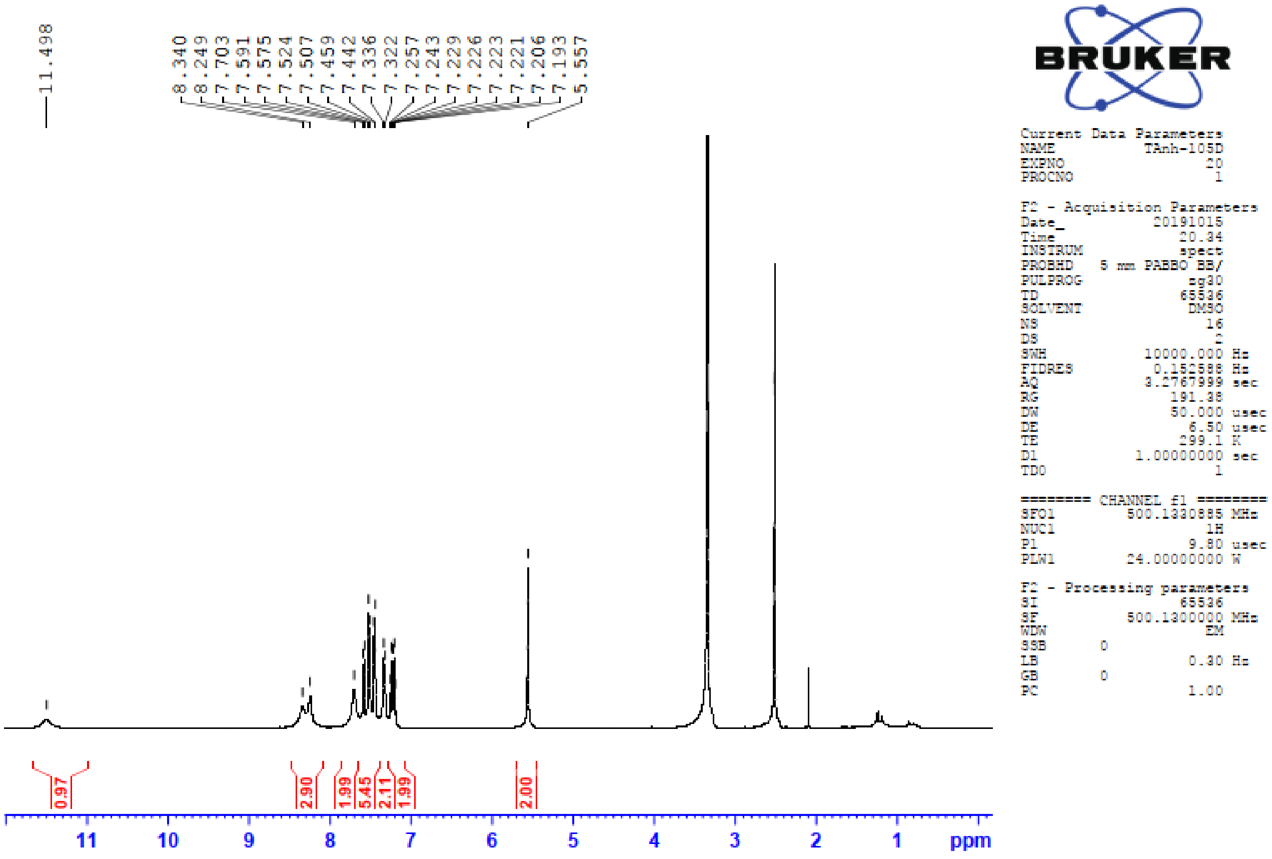

^{1}H-NMR of compound 4d

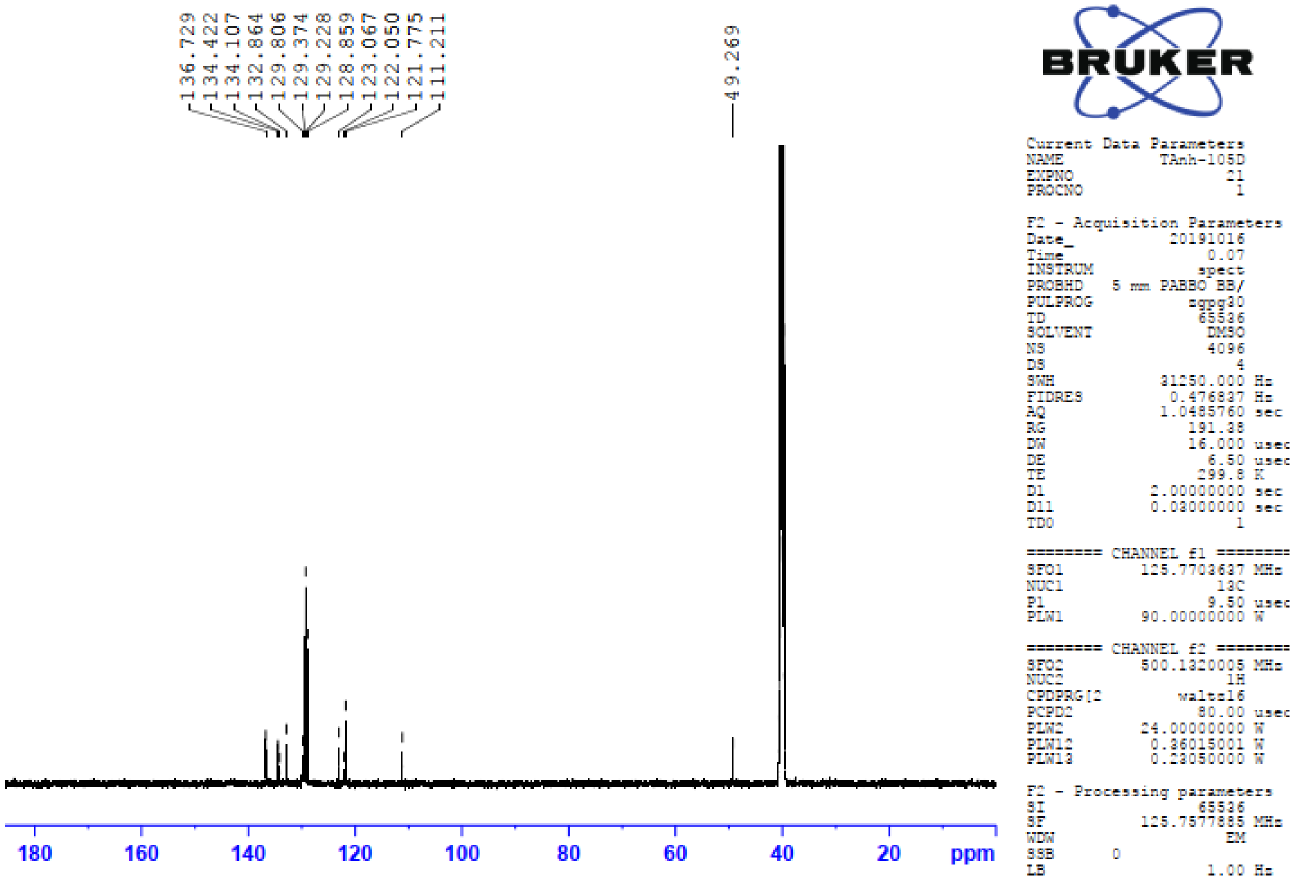

^{13}C-NMR of compound 4d

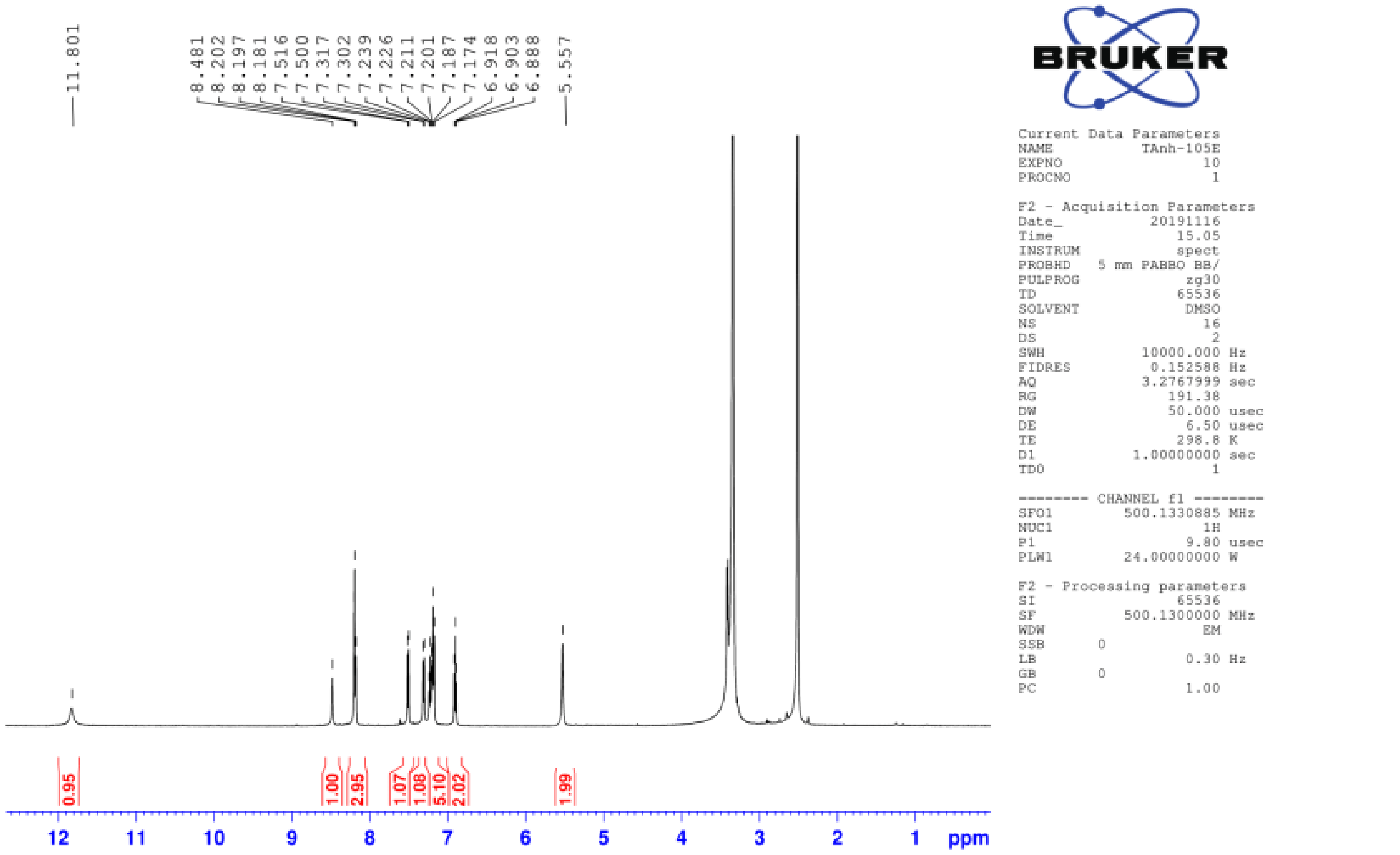

^{1}H-NMR of compound 4e

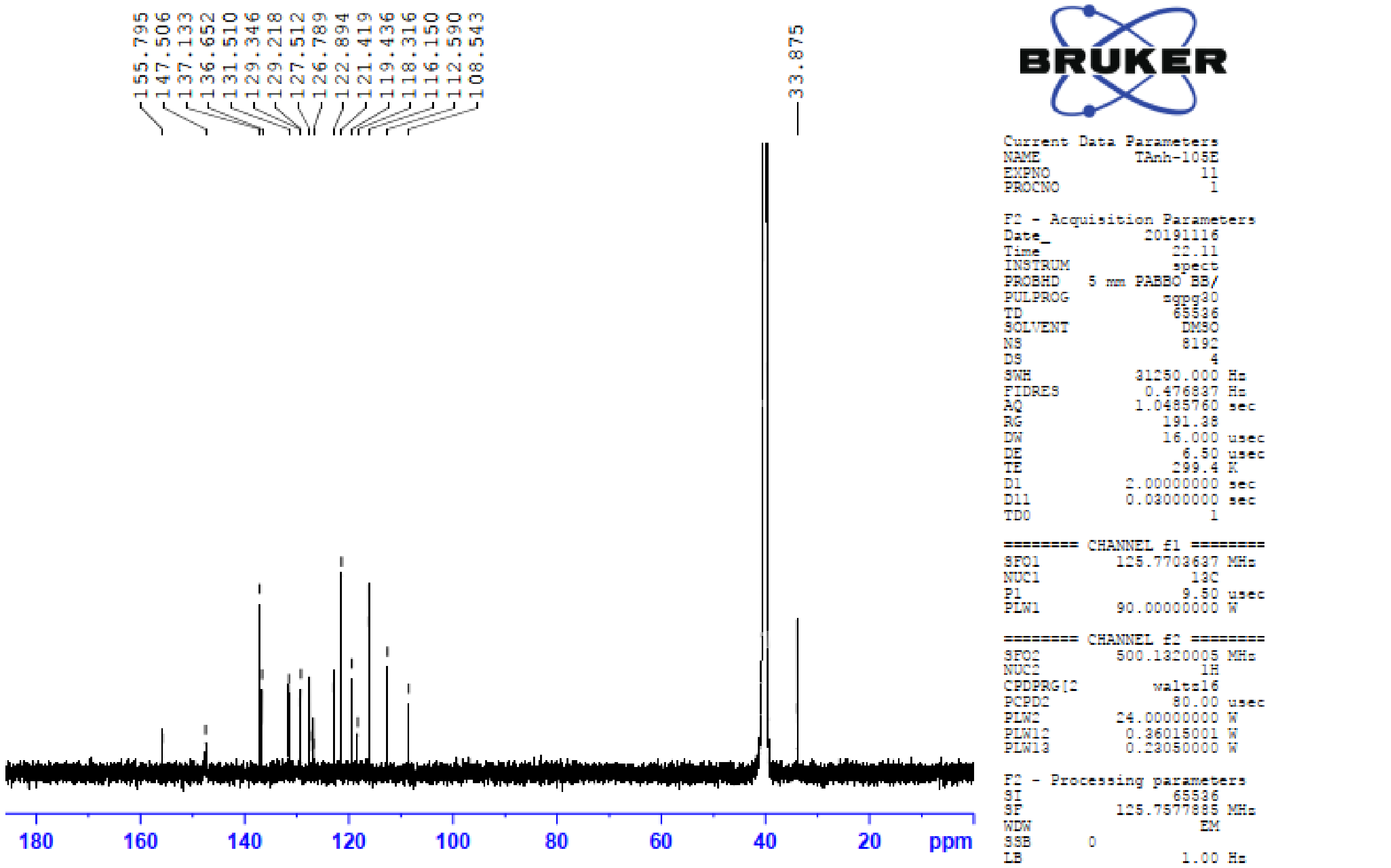

^{13}C-NMR of compound 4e

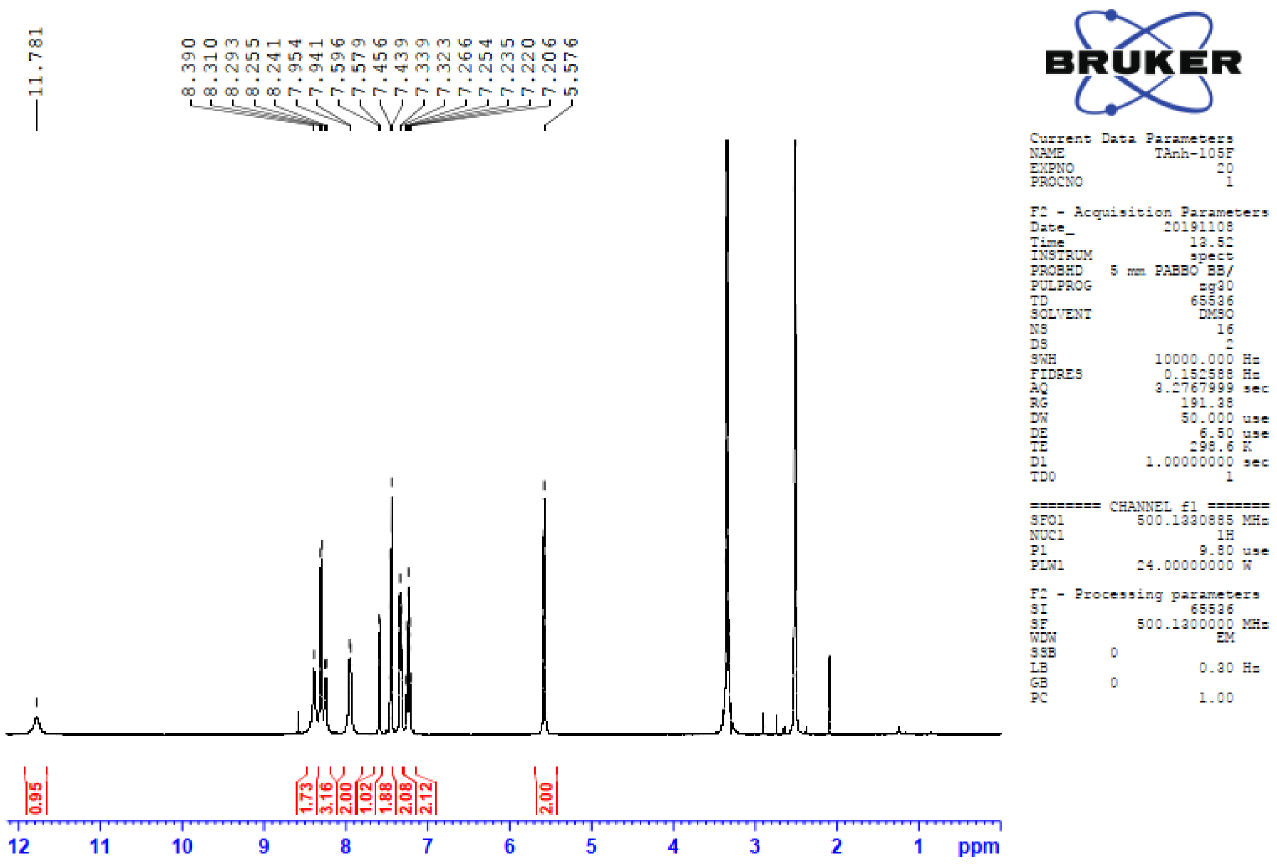

^{1}H-NMR of compound 4f

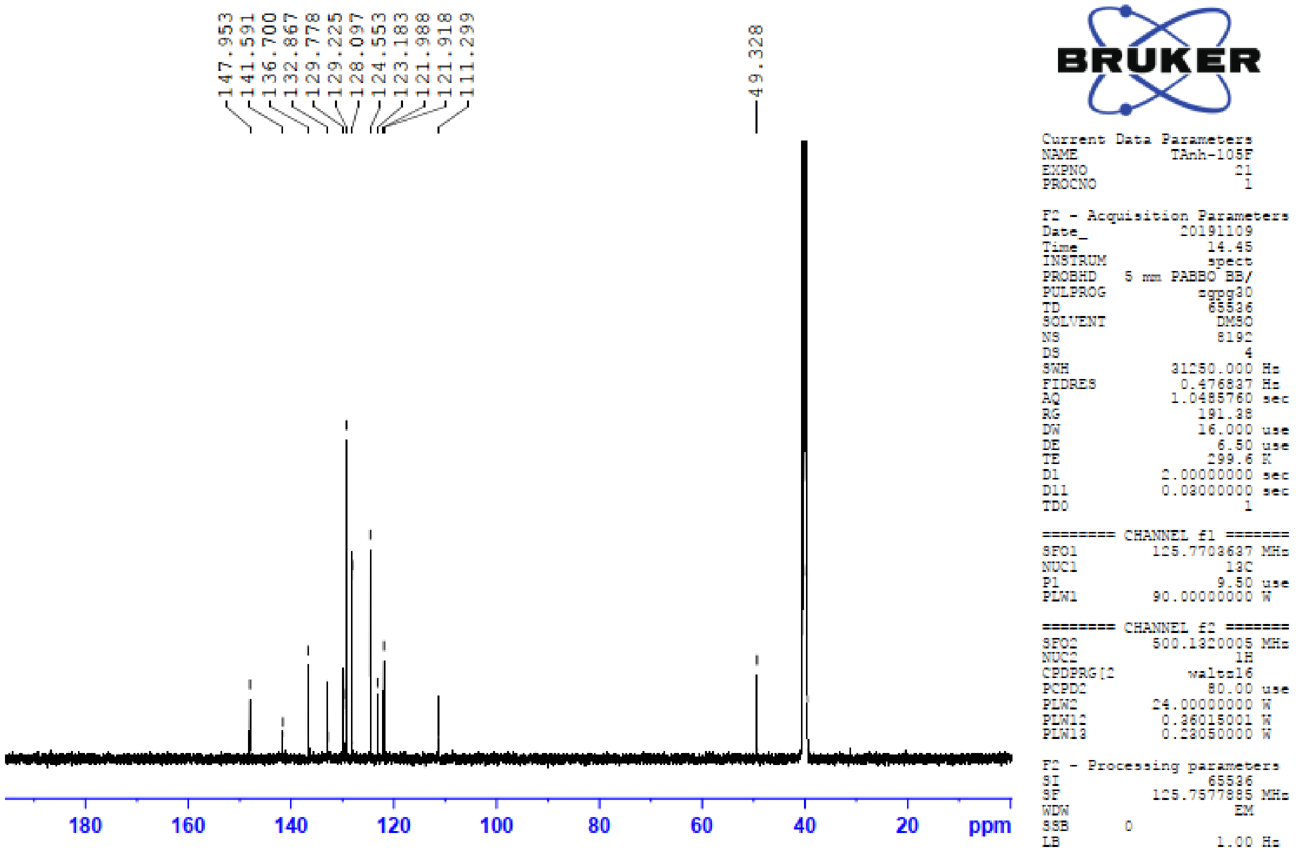

^{13}C-NMR of compound 4f

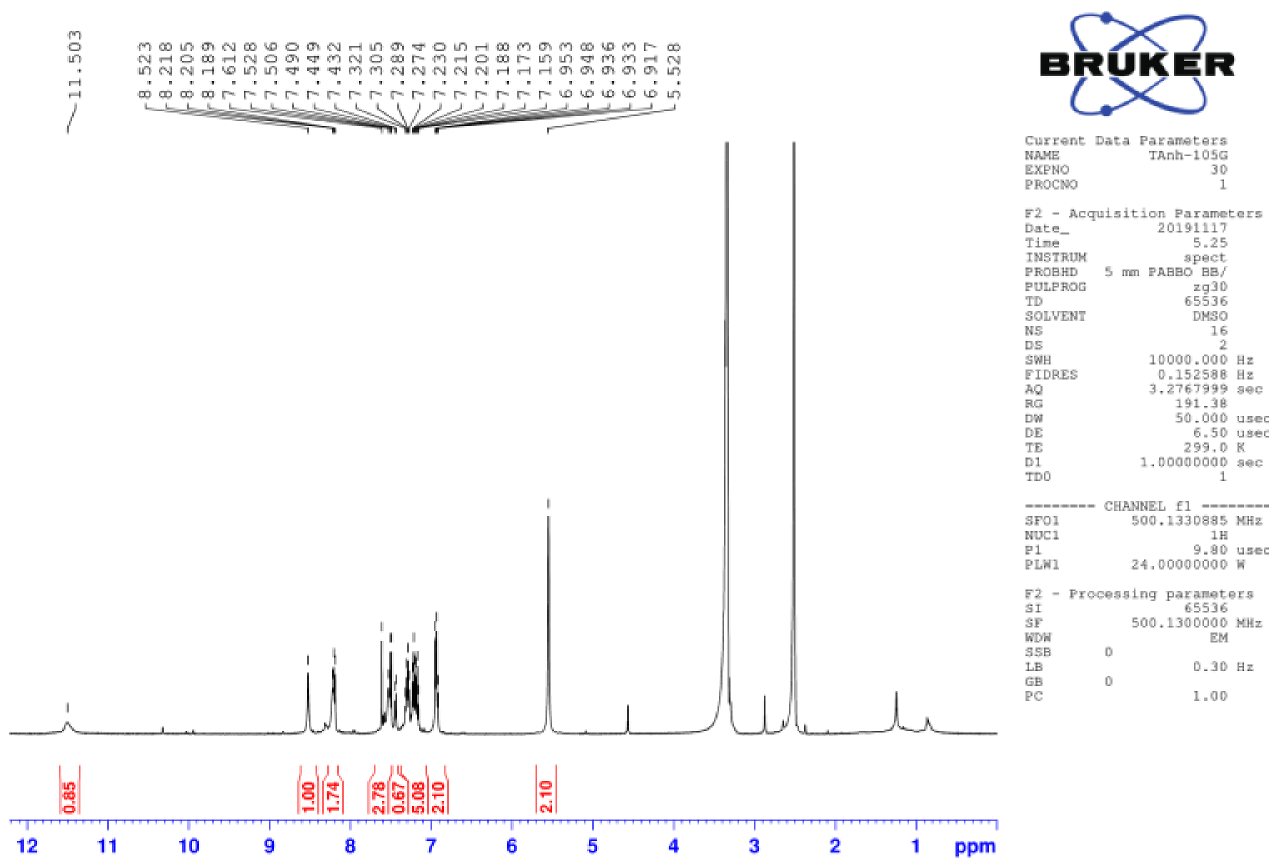

^{1}H-NMR of compound 4g

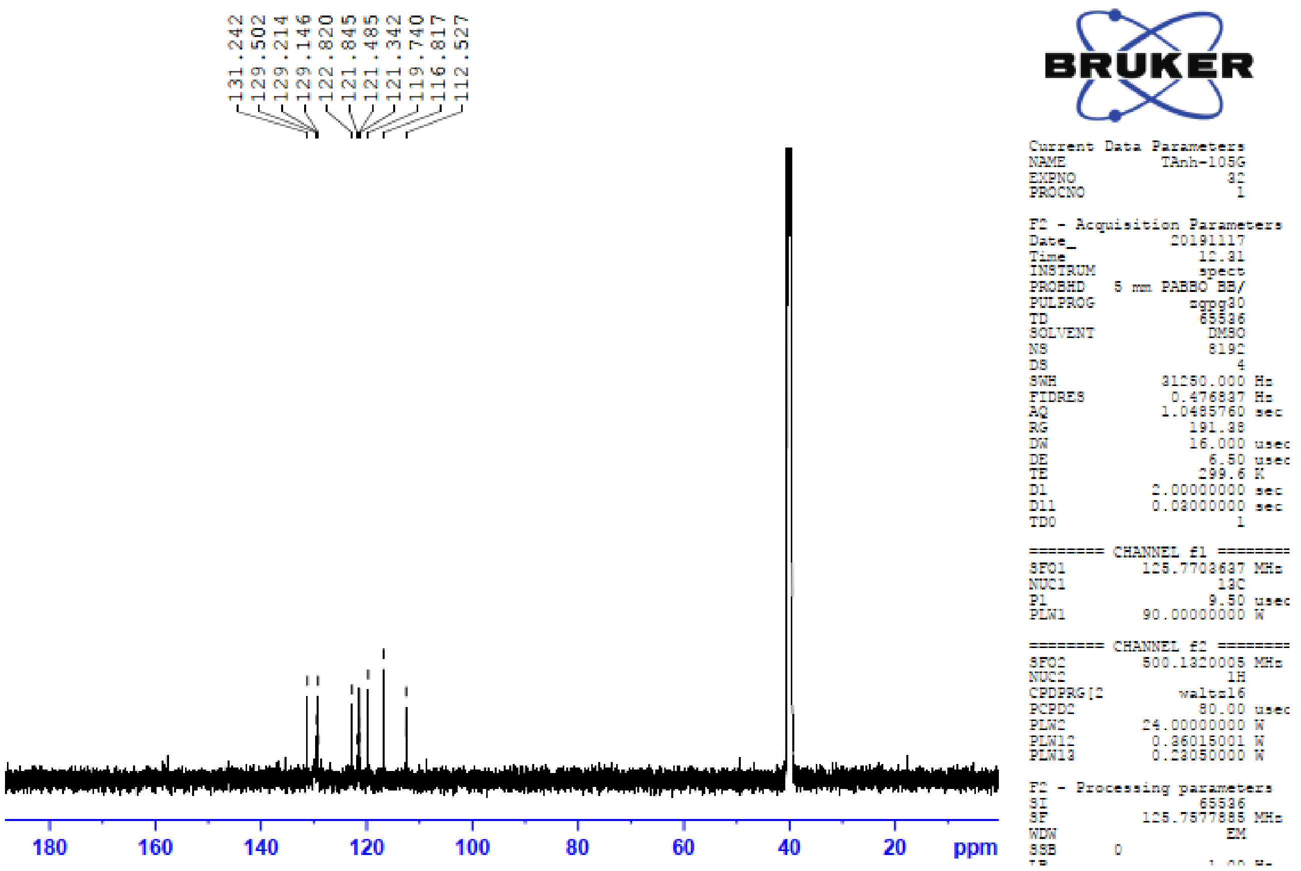

^{13}C-NMR of compound 4g

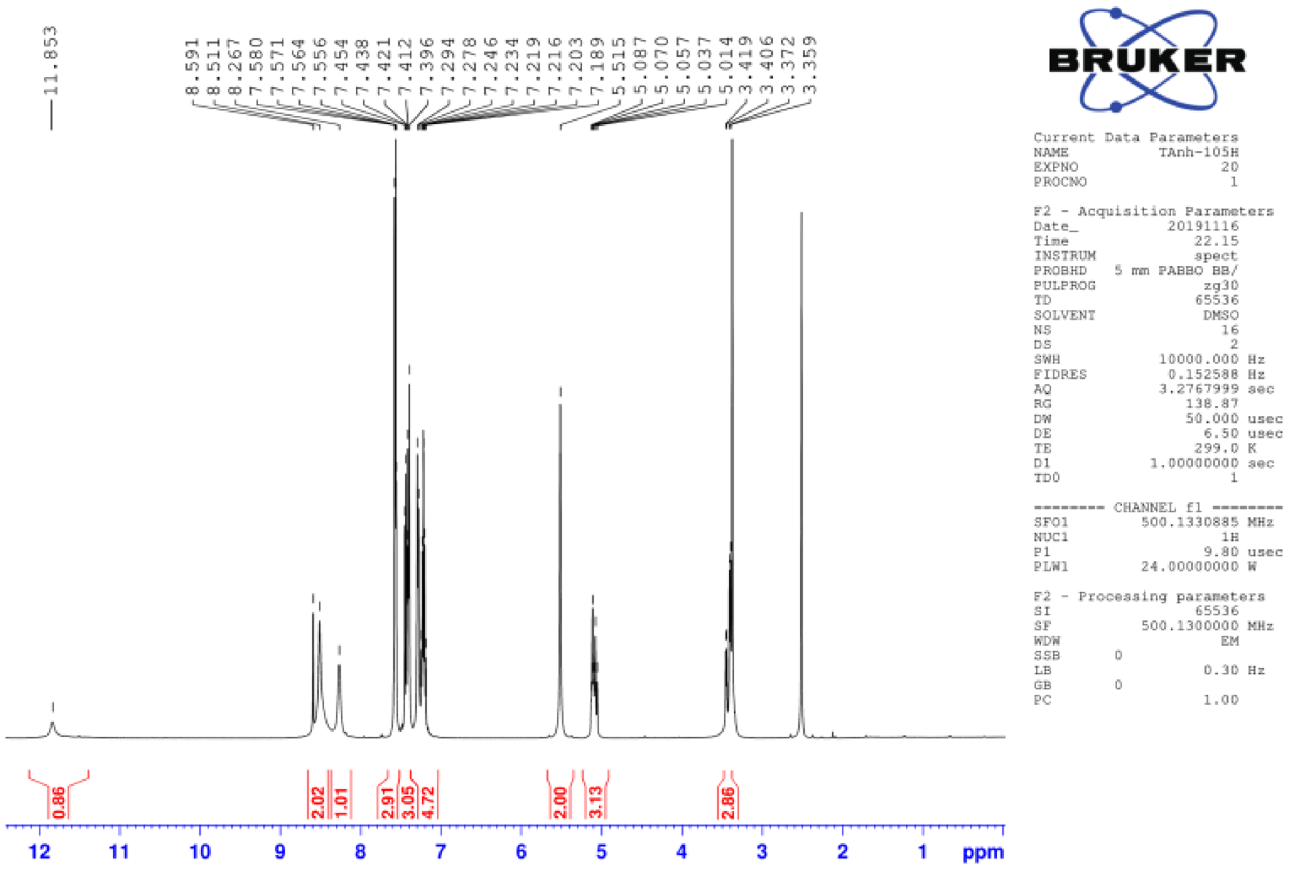

^{1}H-NMR of compound 4h

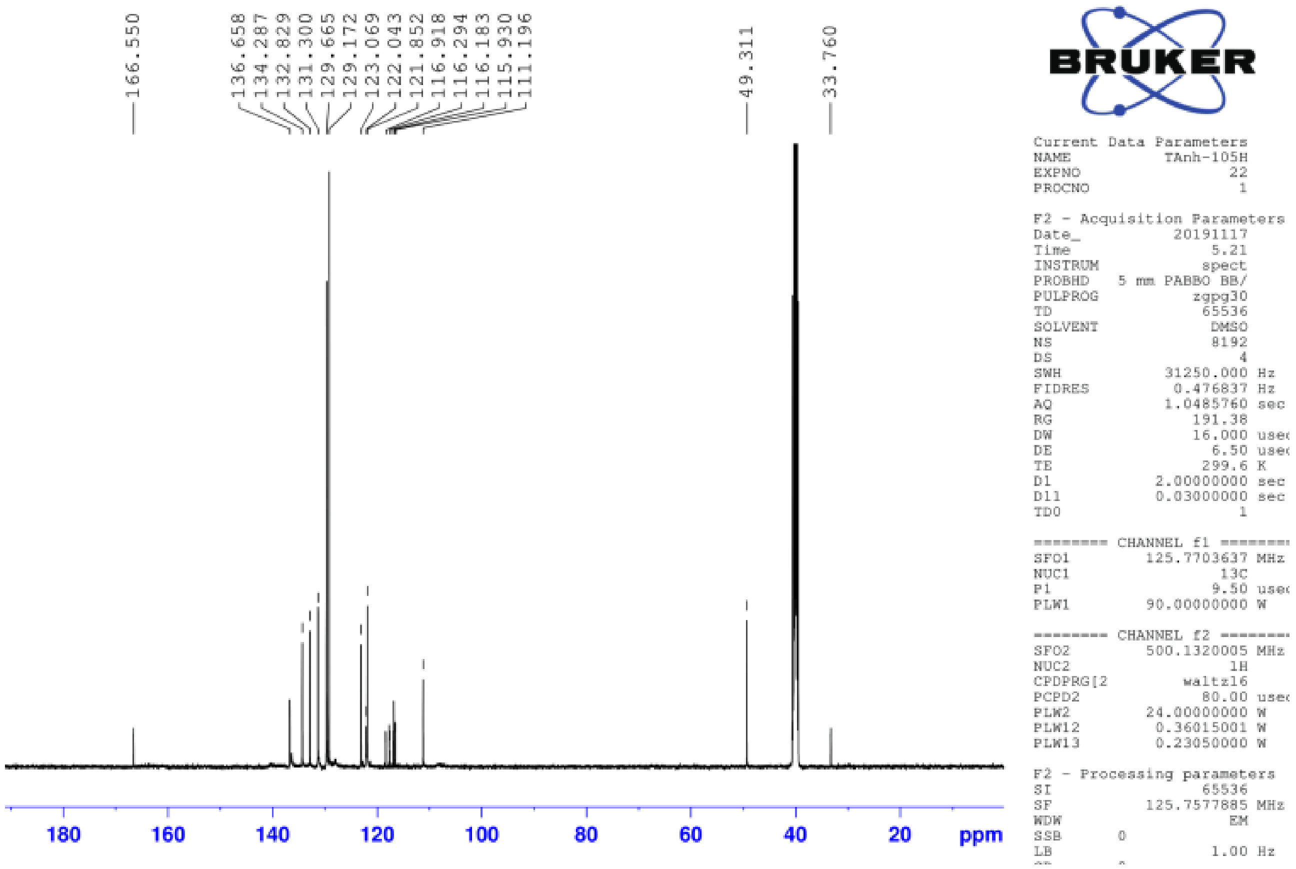

^{13}C-NMR of compound 4h

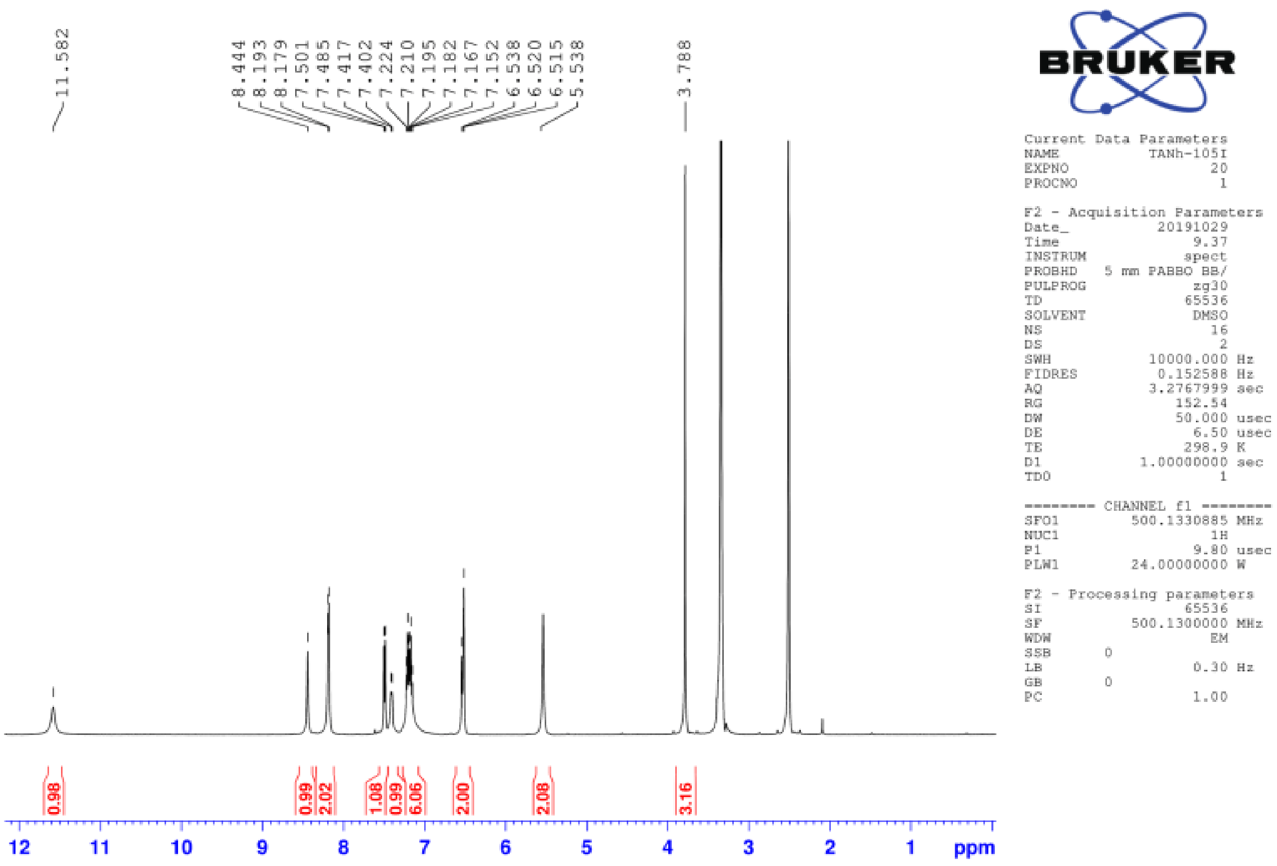

^{1}H-NMR of compound 4i

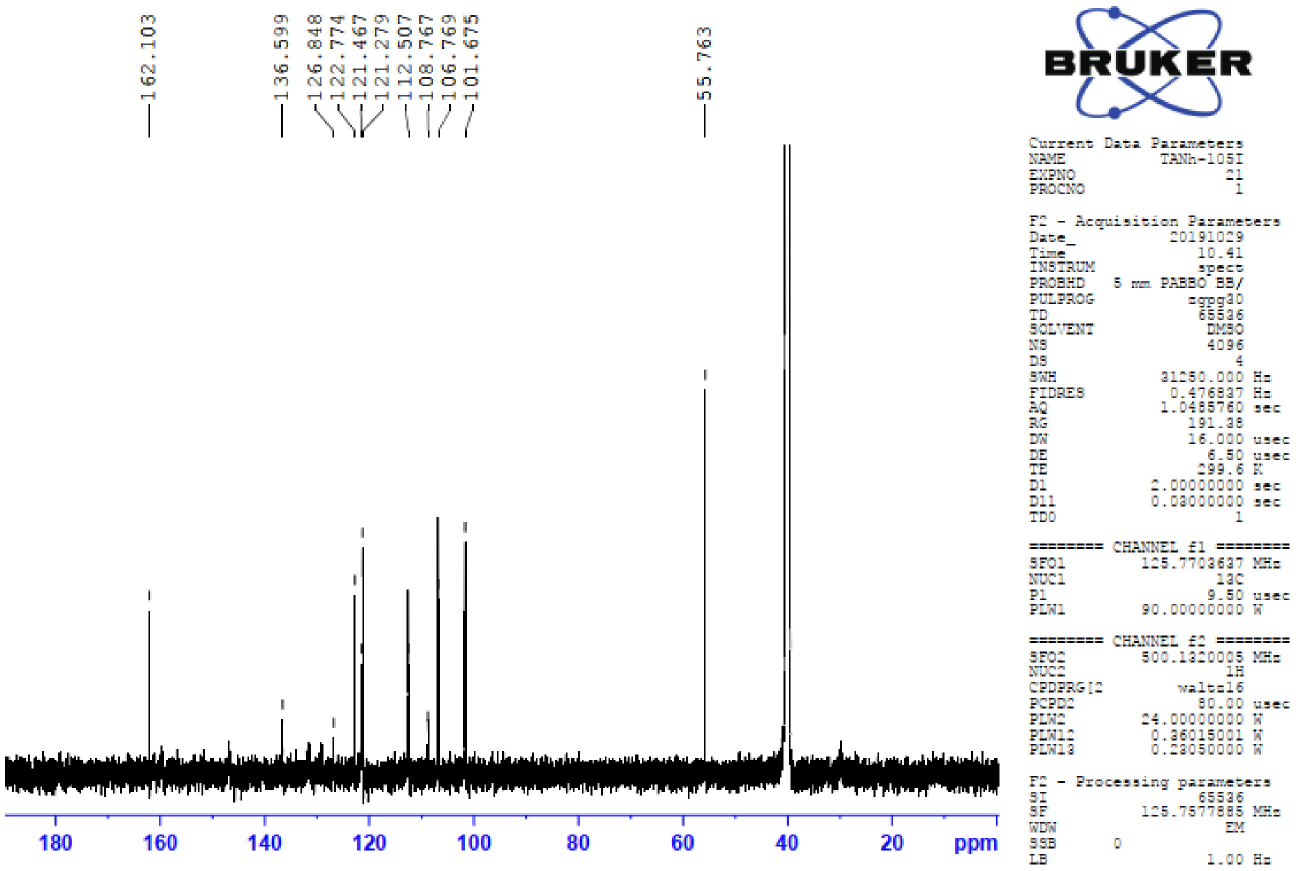

^{13}C-NMR of compound 4i

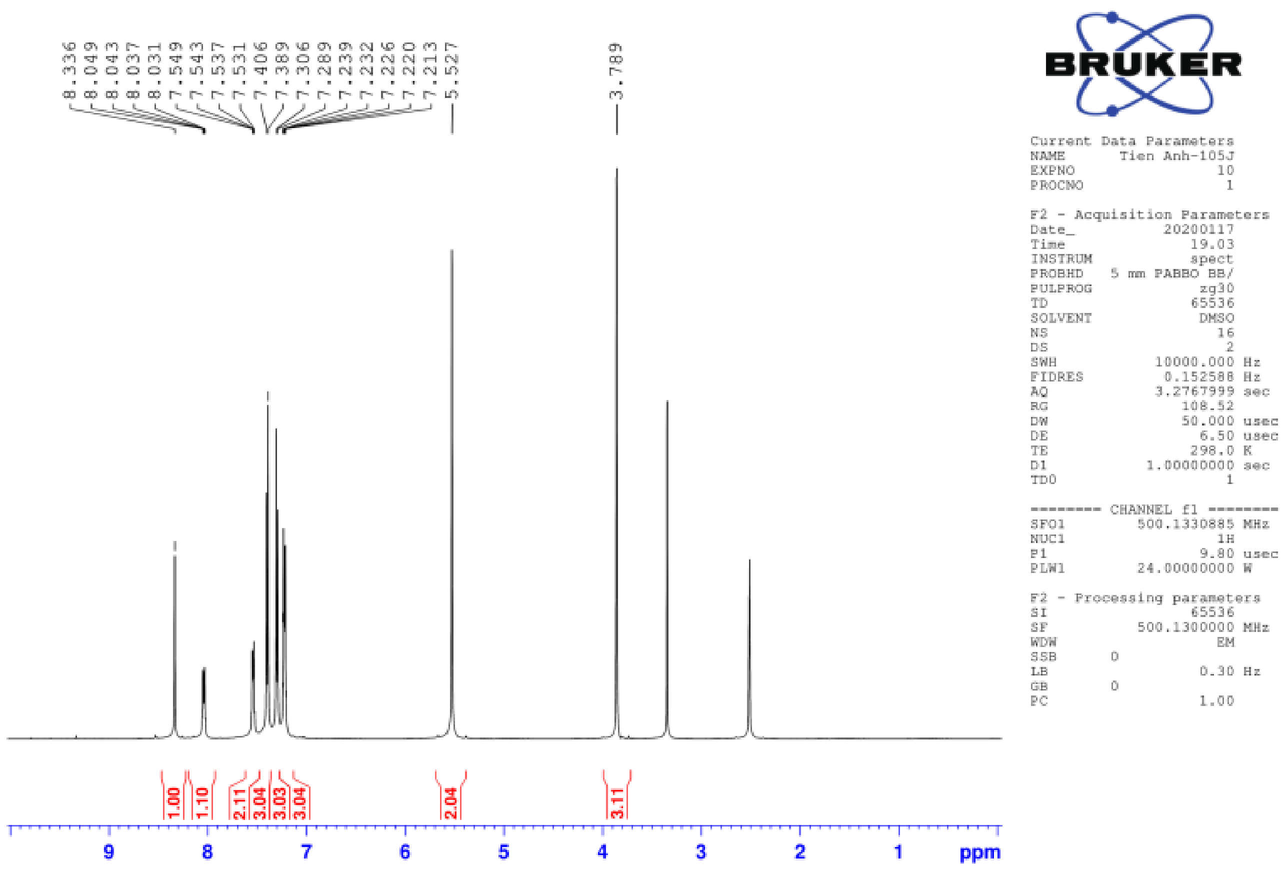

^{1}H-NMR of compound 4j

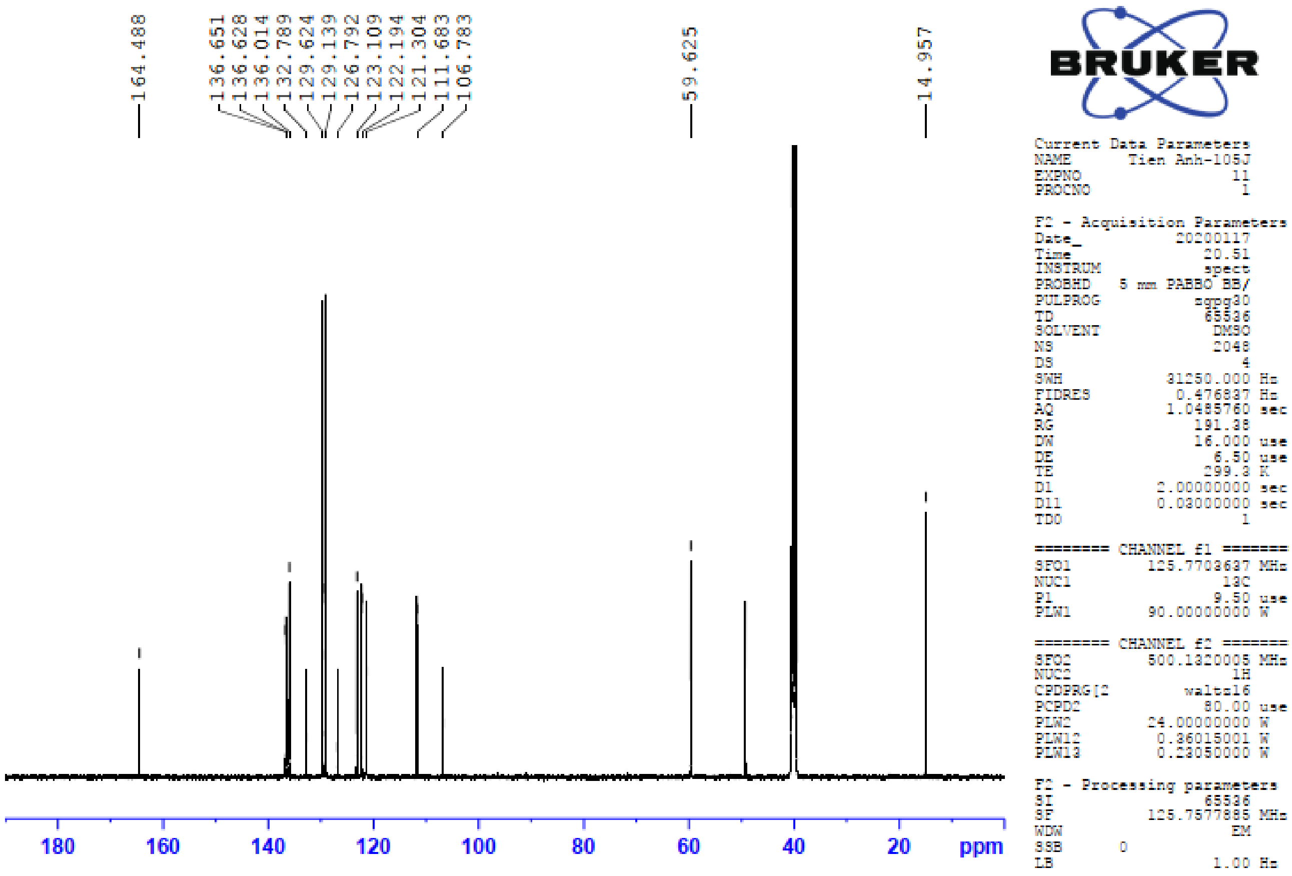

^{13}C-NMR of compound 4j

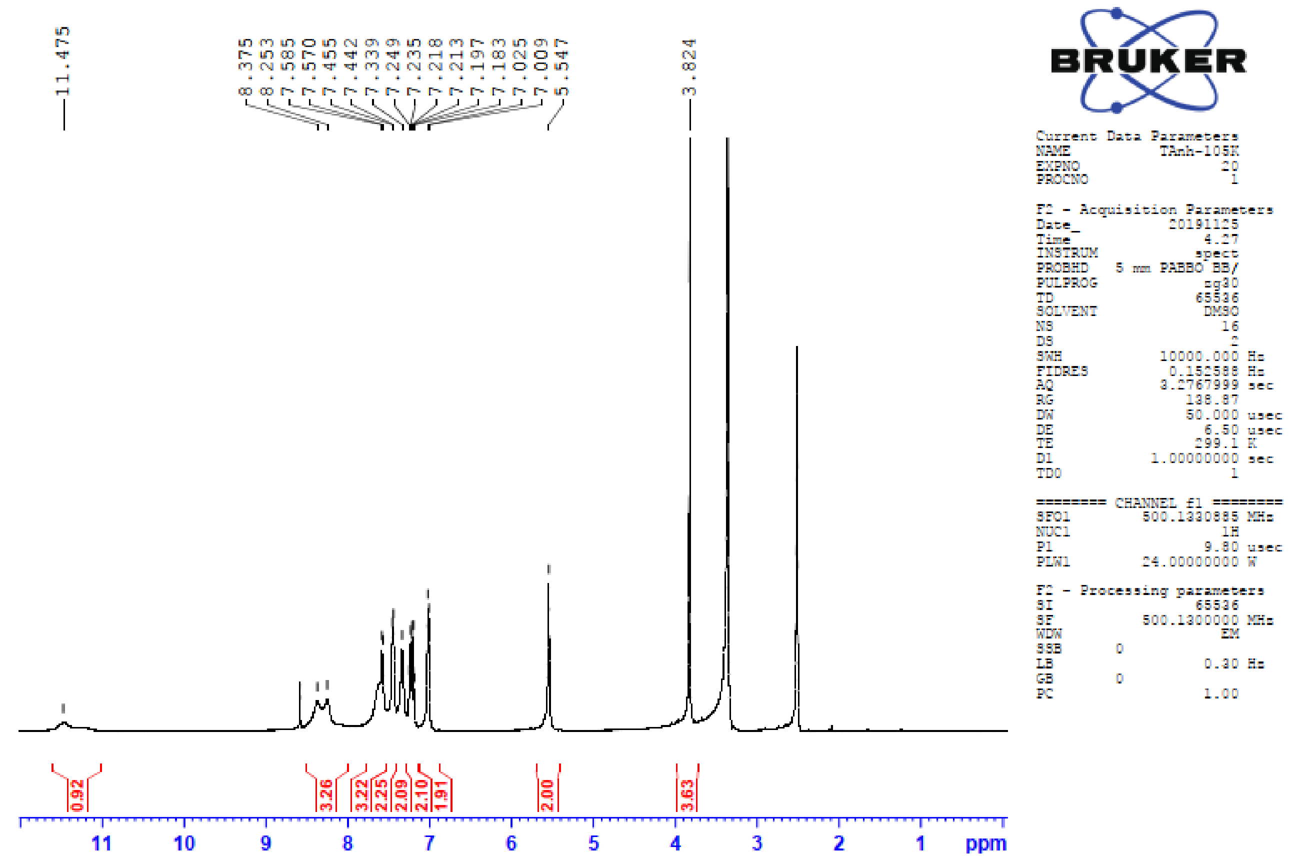

^{1}H-NMR of compound 4k

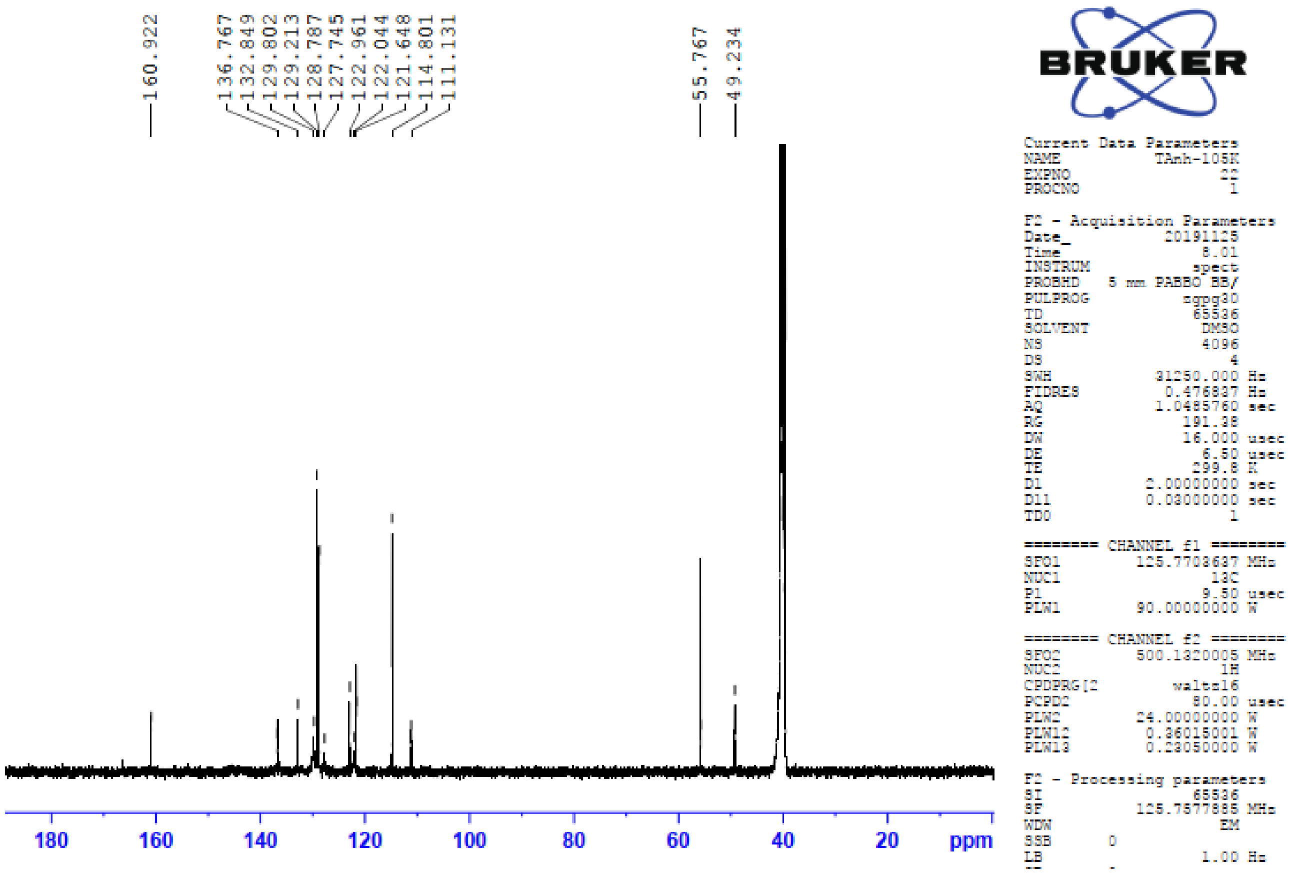

^{13}C-NMR of compound 4k

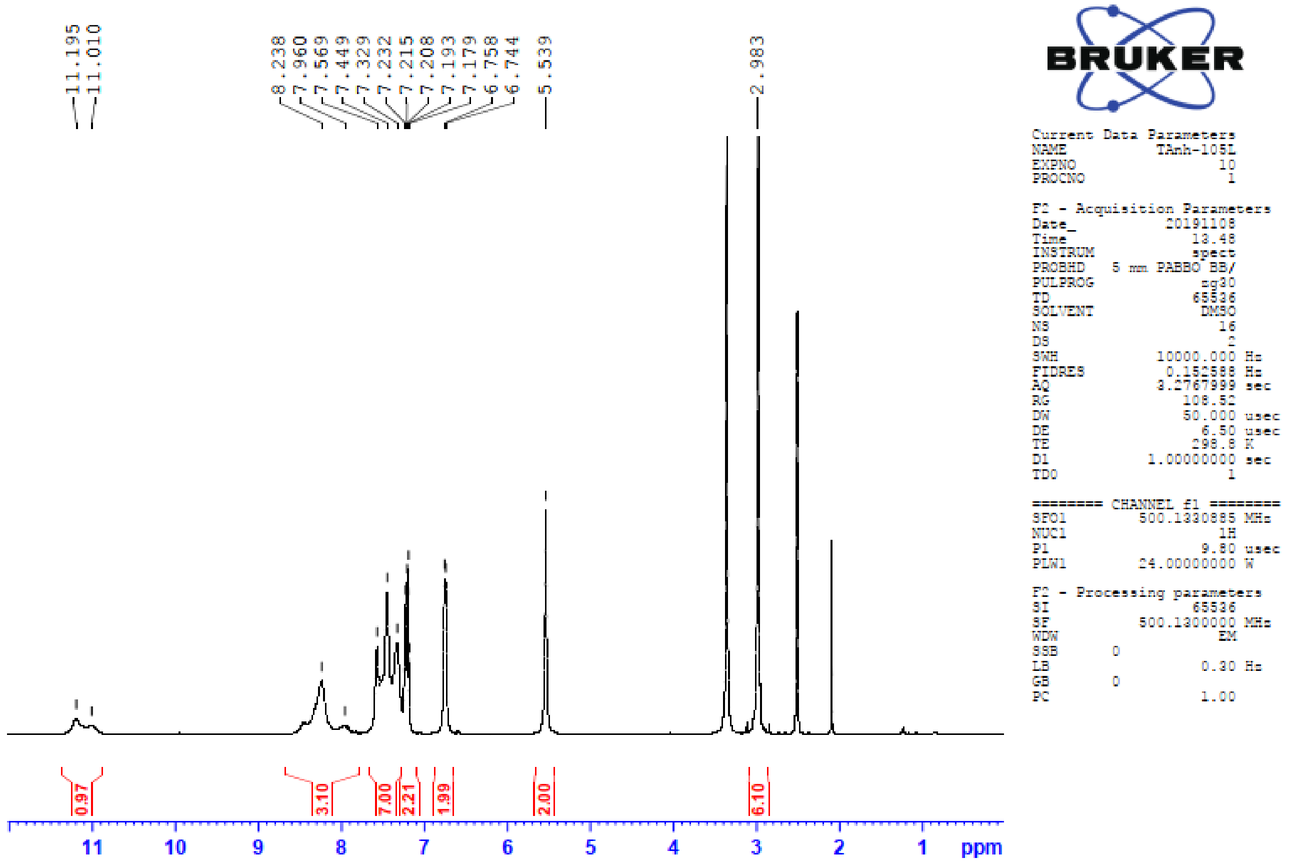

^{1}H-NMR of compound 4l

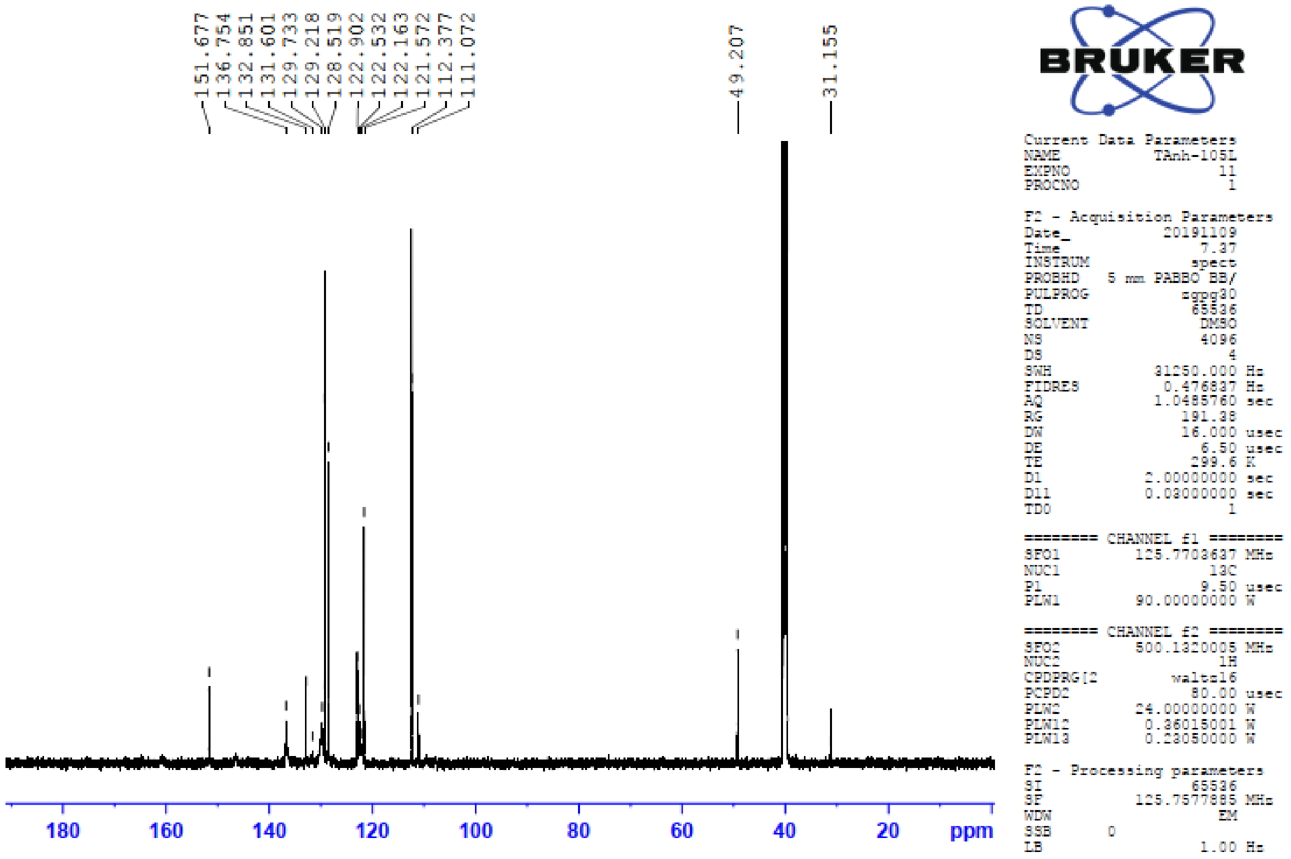

^{13}C-NMR of compound 4l

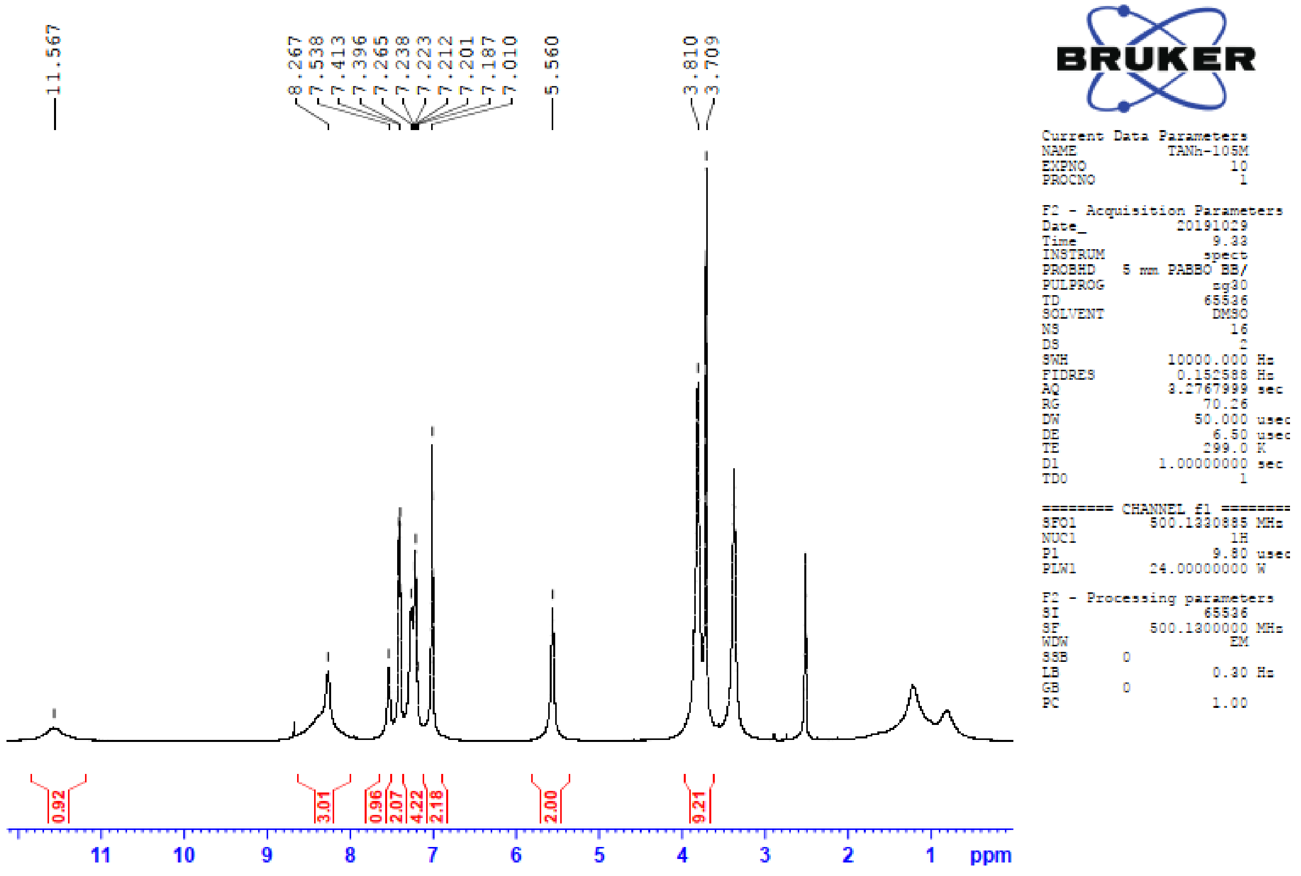

^{1}H-NMR of compound 4m

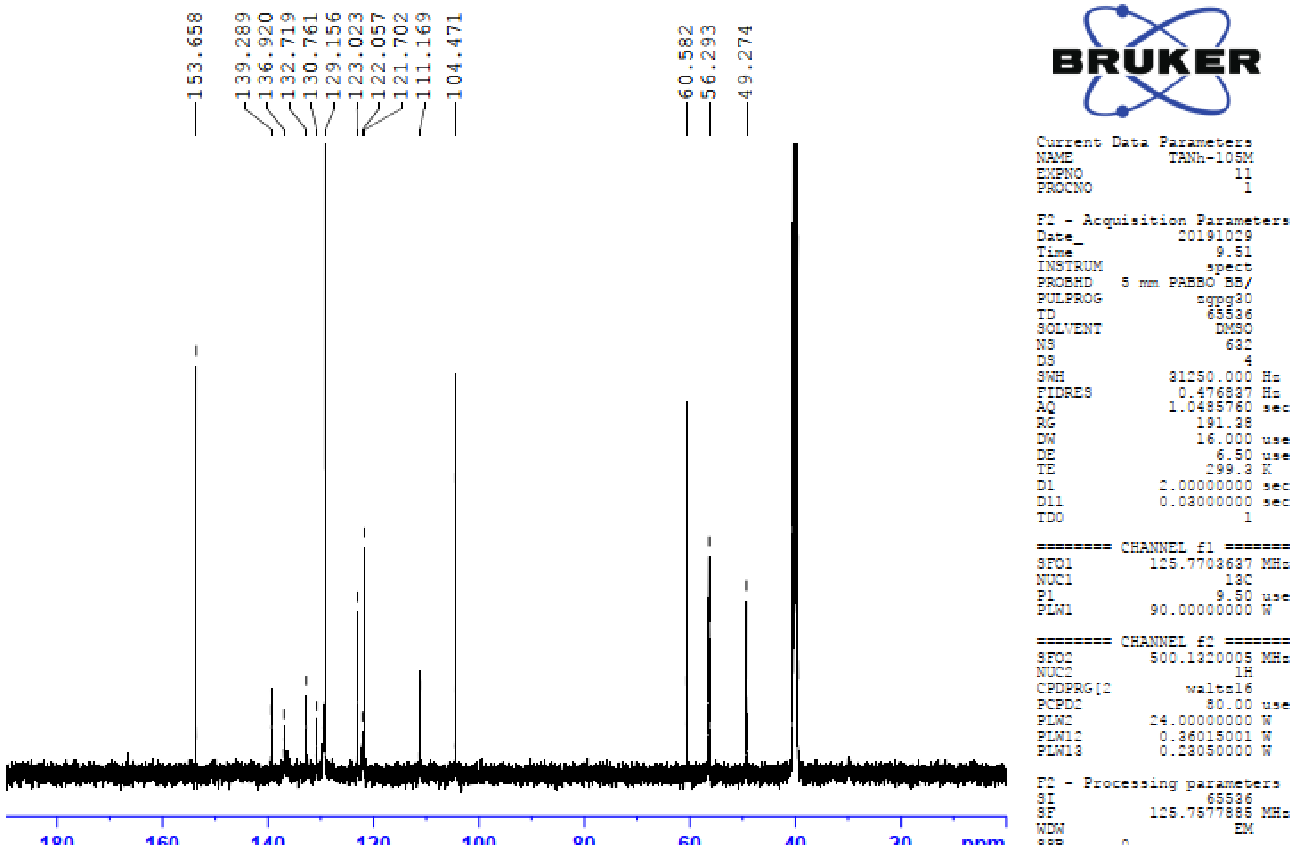

^{13}C-NMR of compound 4m

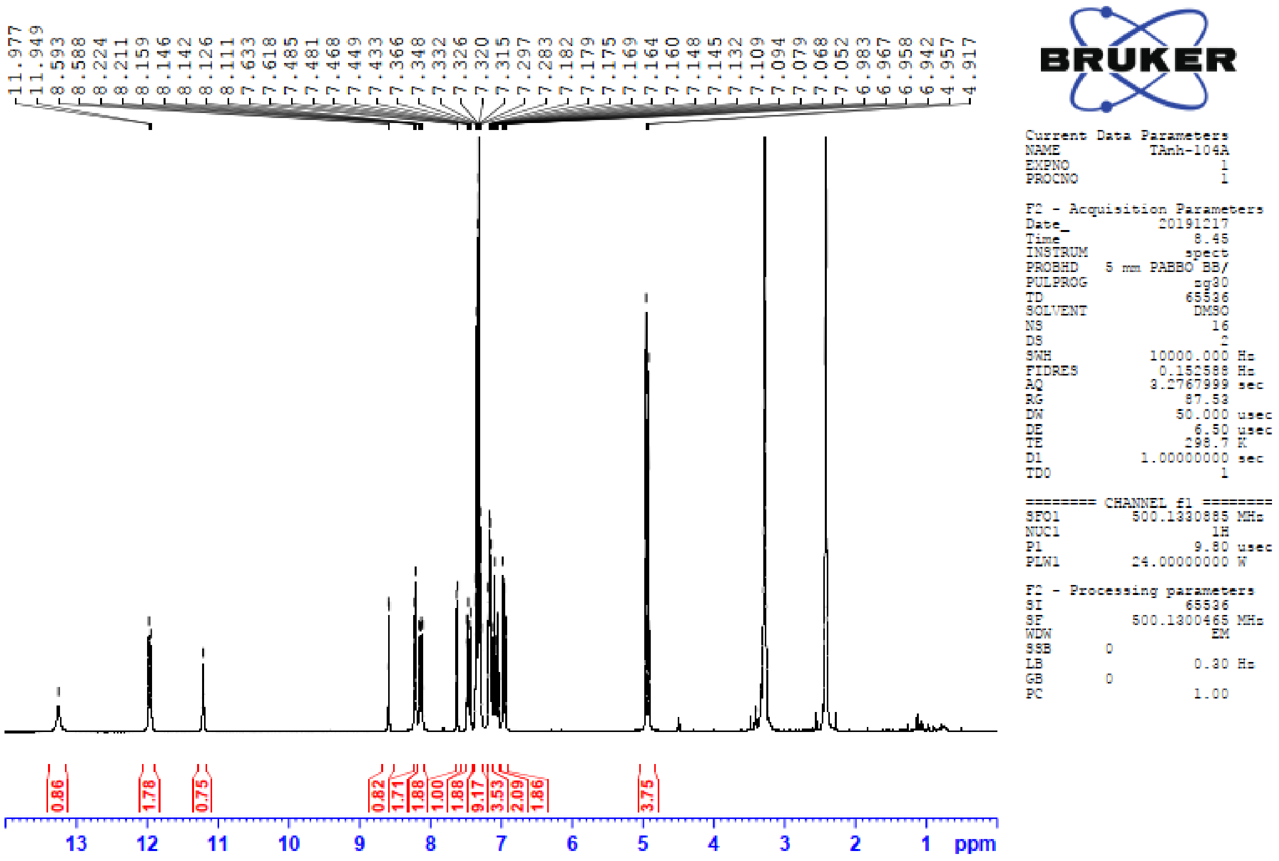

^{1}H-NMR of compound 5a

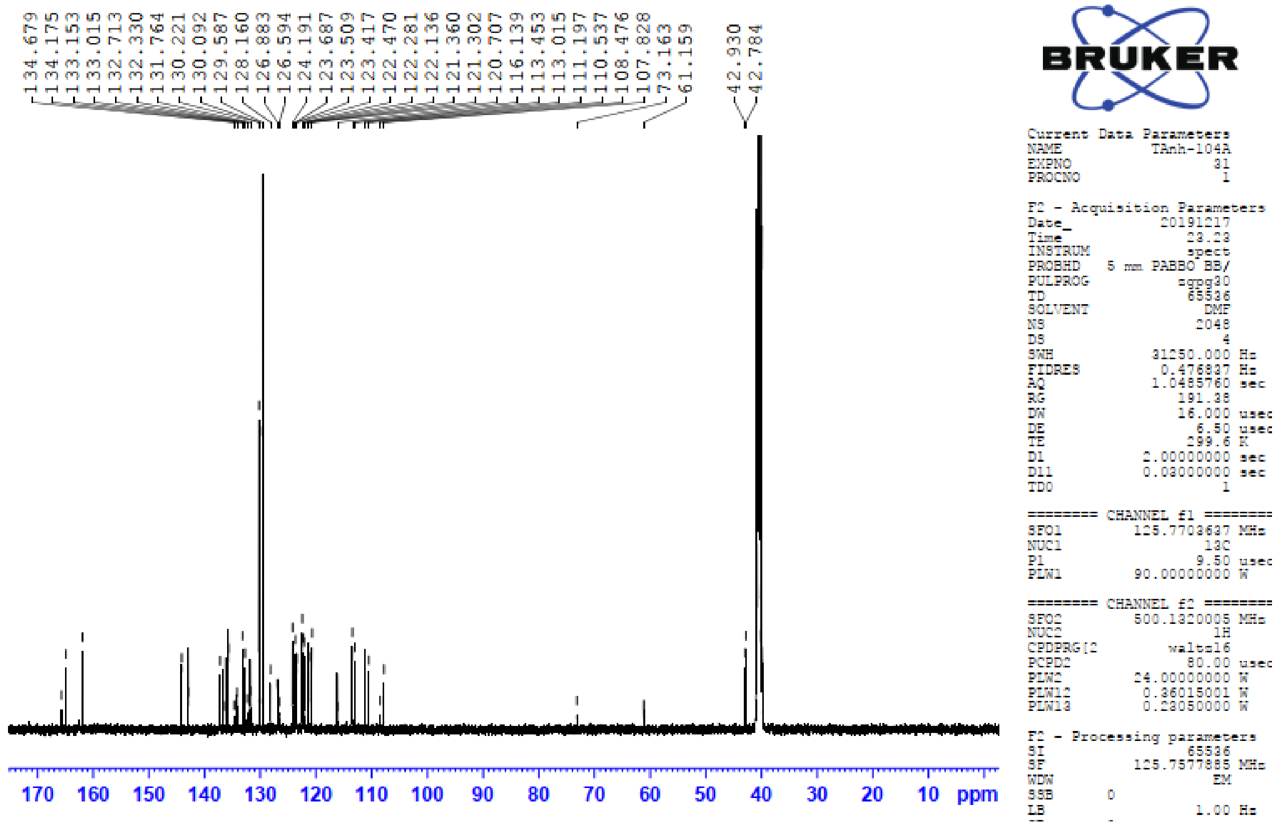

^{13}C-NMR of compound 5a

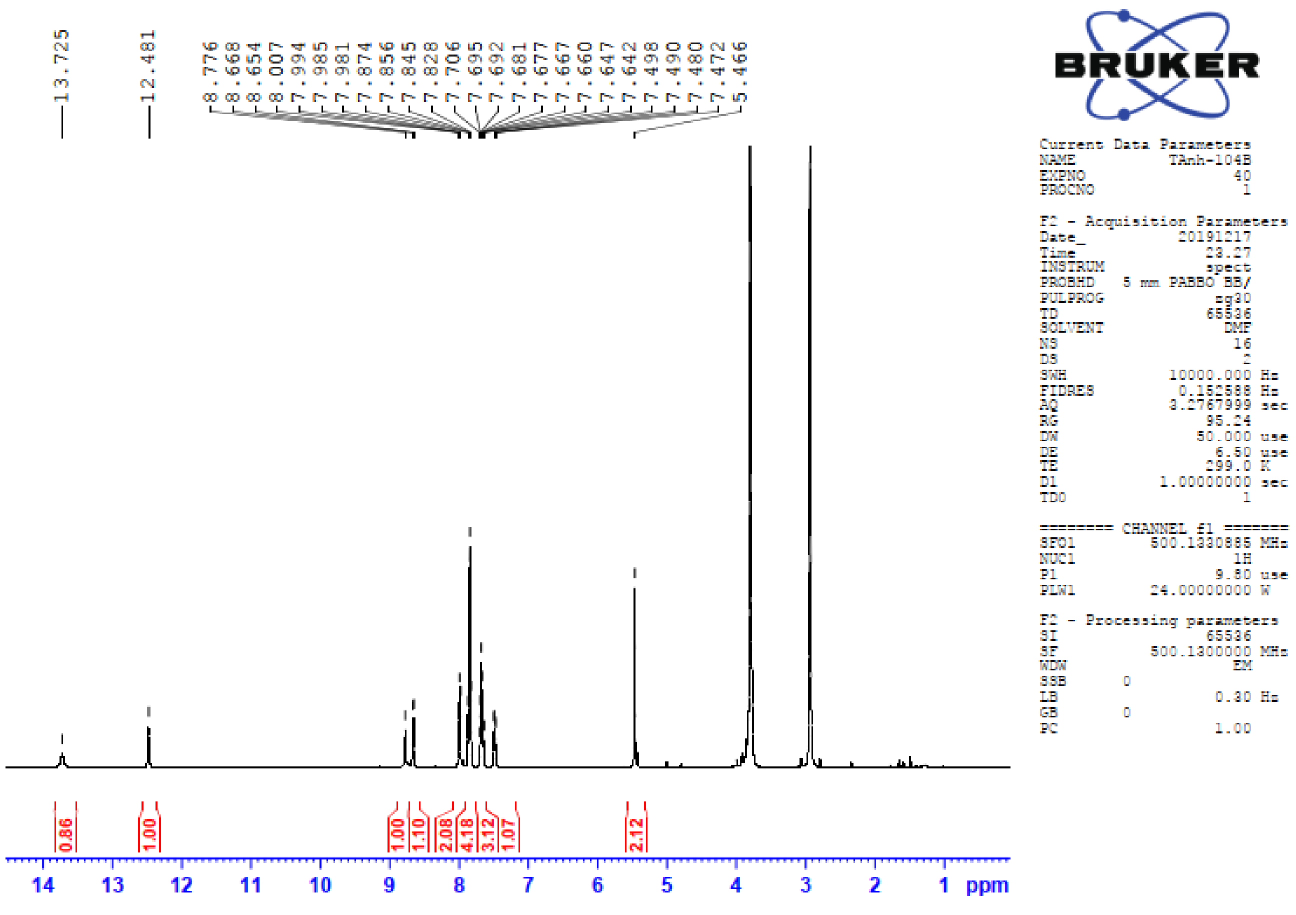

^{1}H-NMR of compound 5b

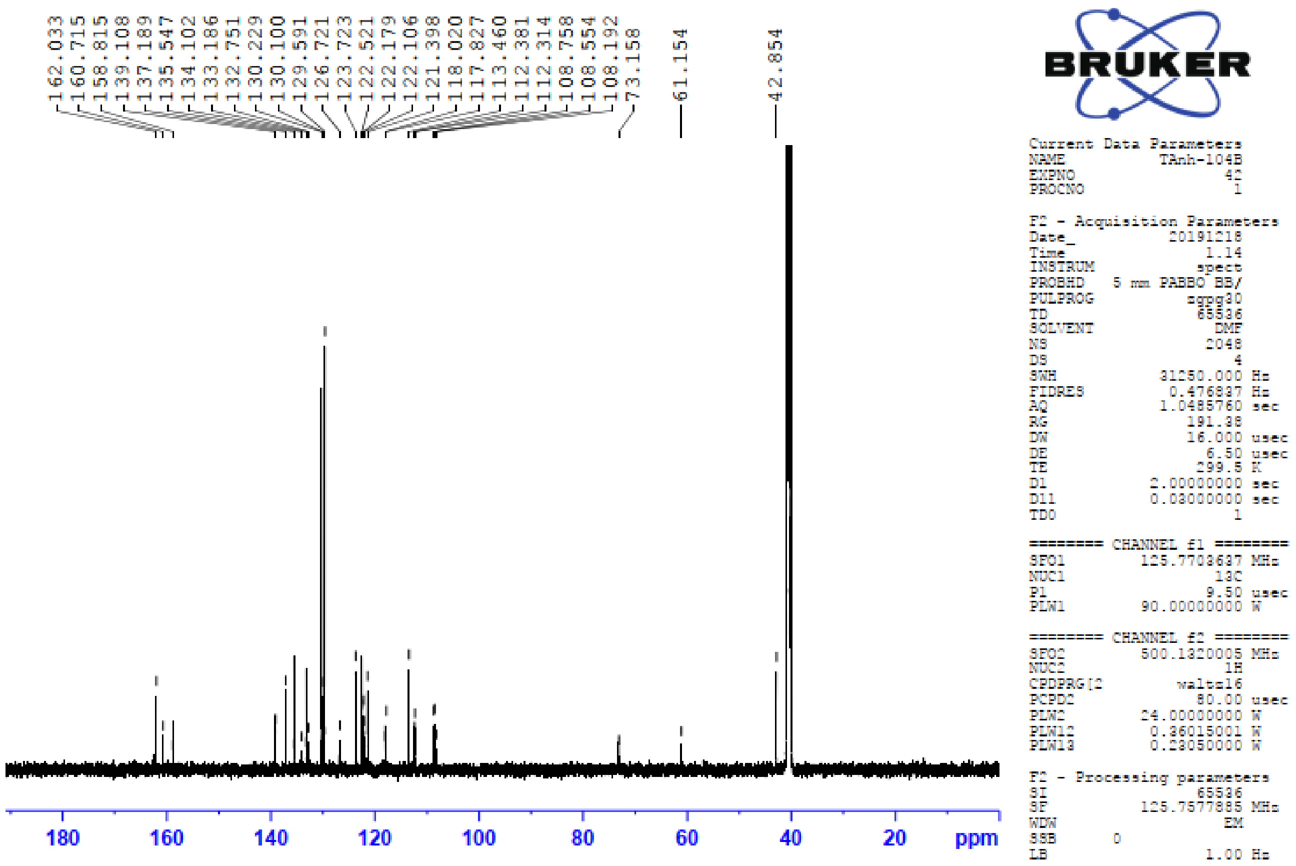

^{13}C-NMR of compound 5b

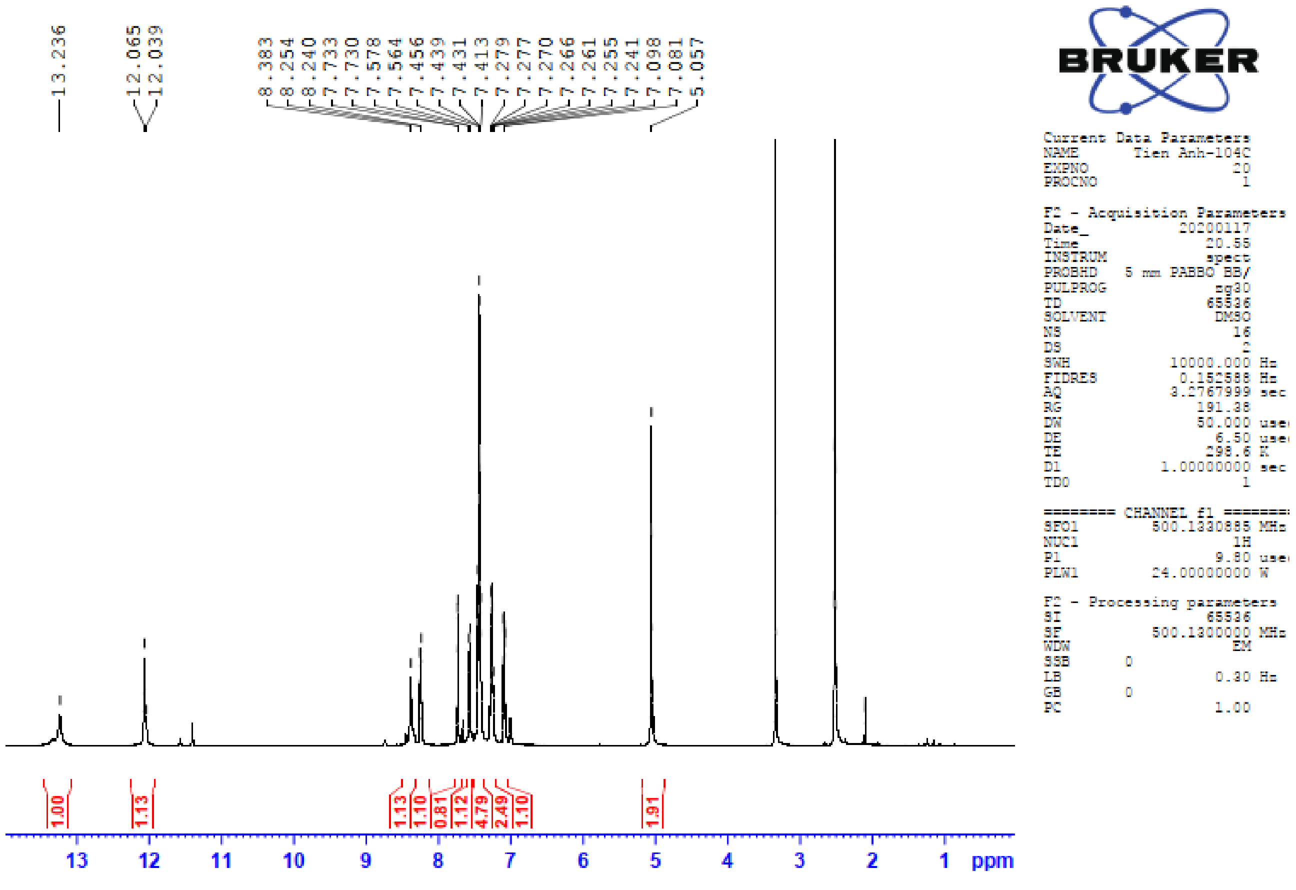

^{1}H-NMR of compound 5c

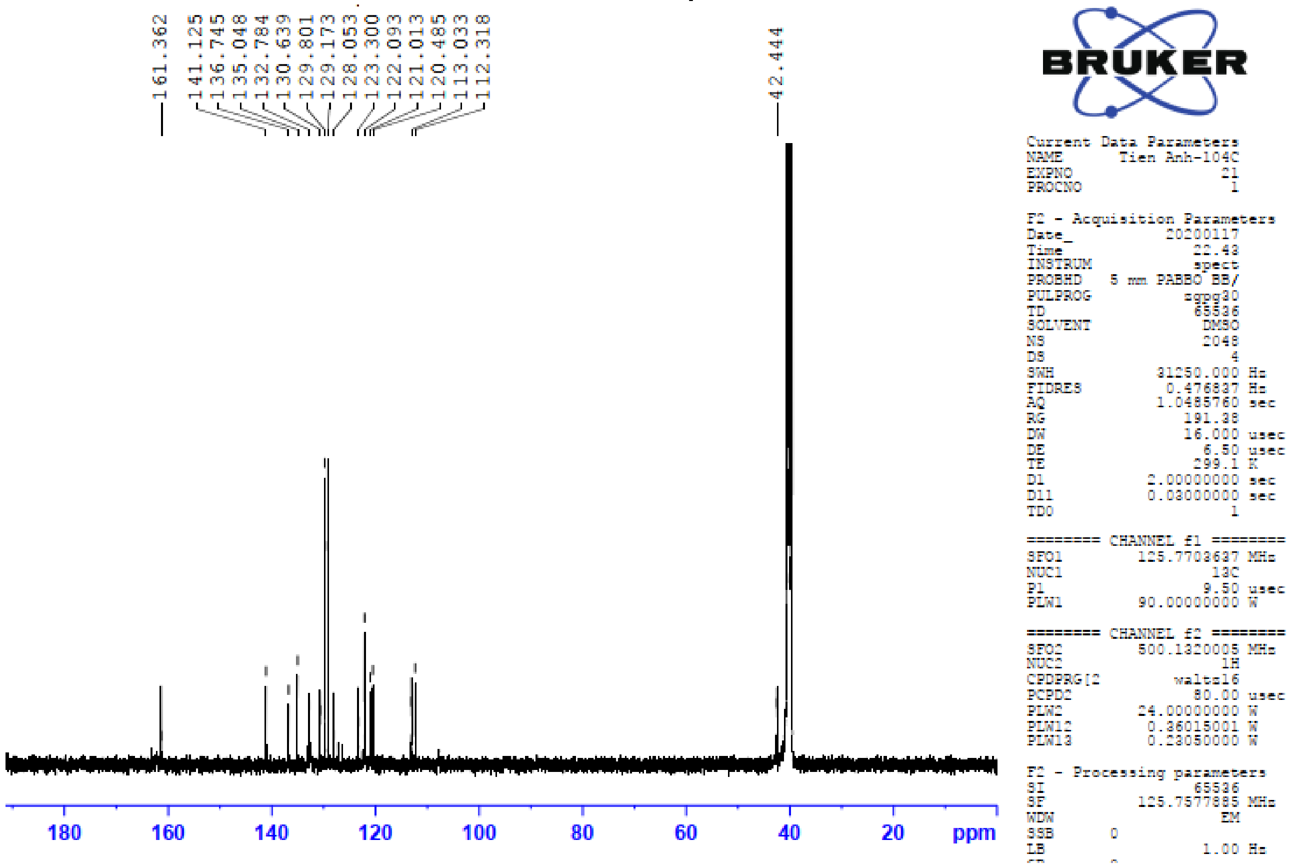

^{13}C-NMR of compound 5c

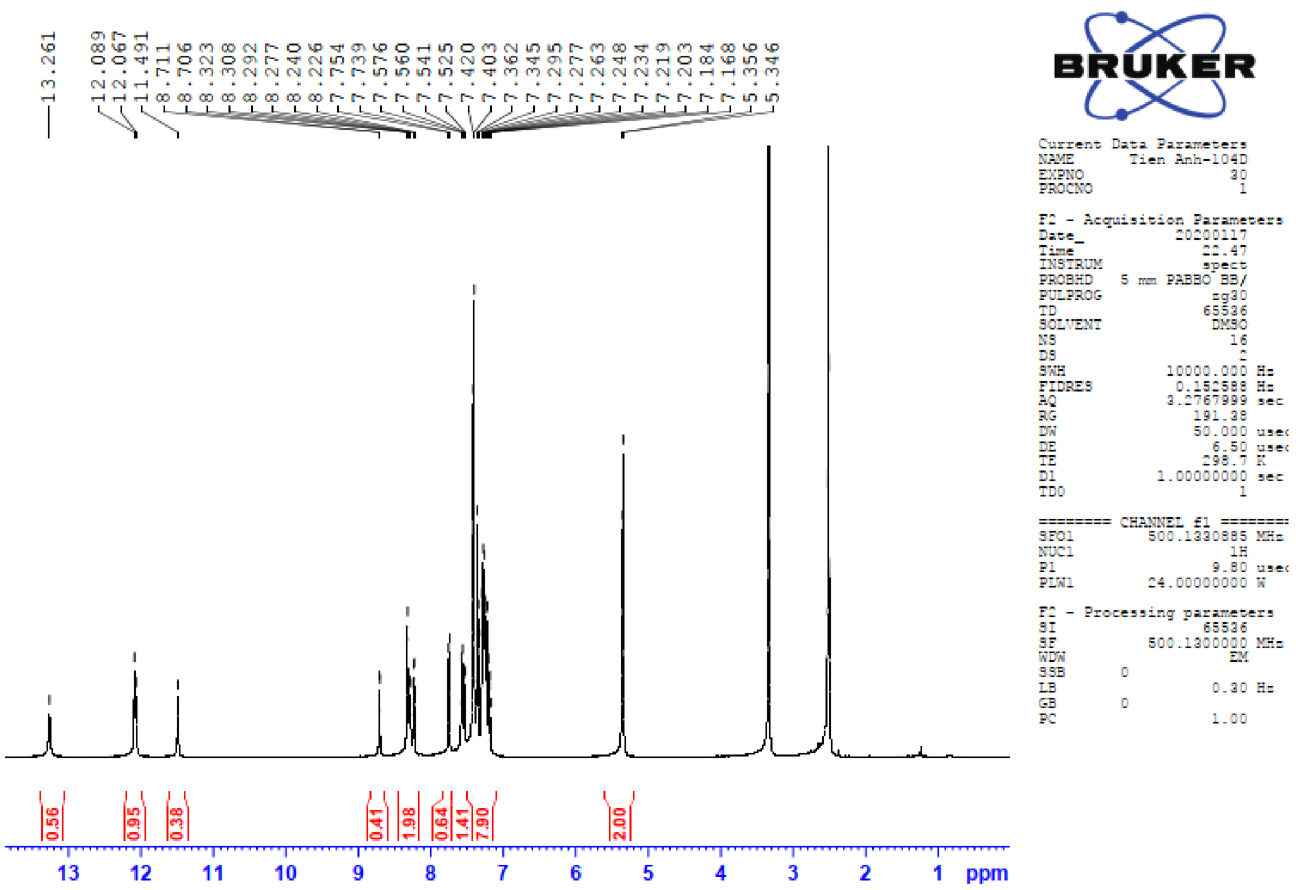

^{1}H-NMR of compound 5d

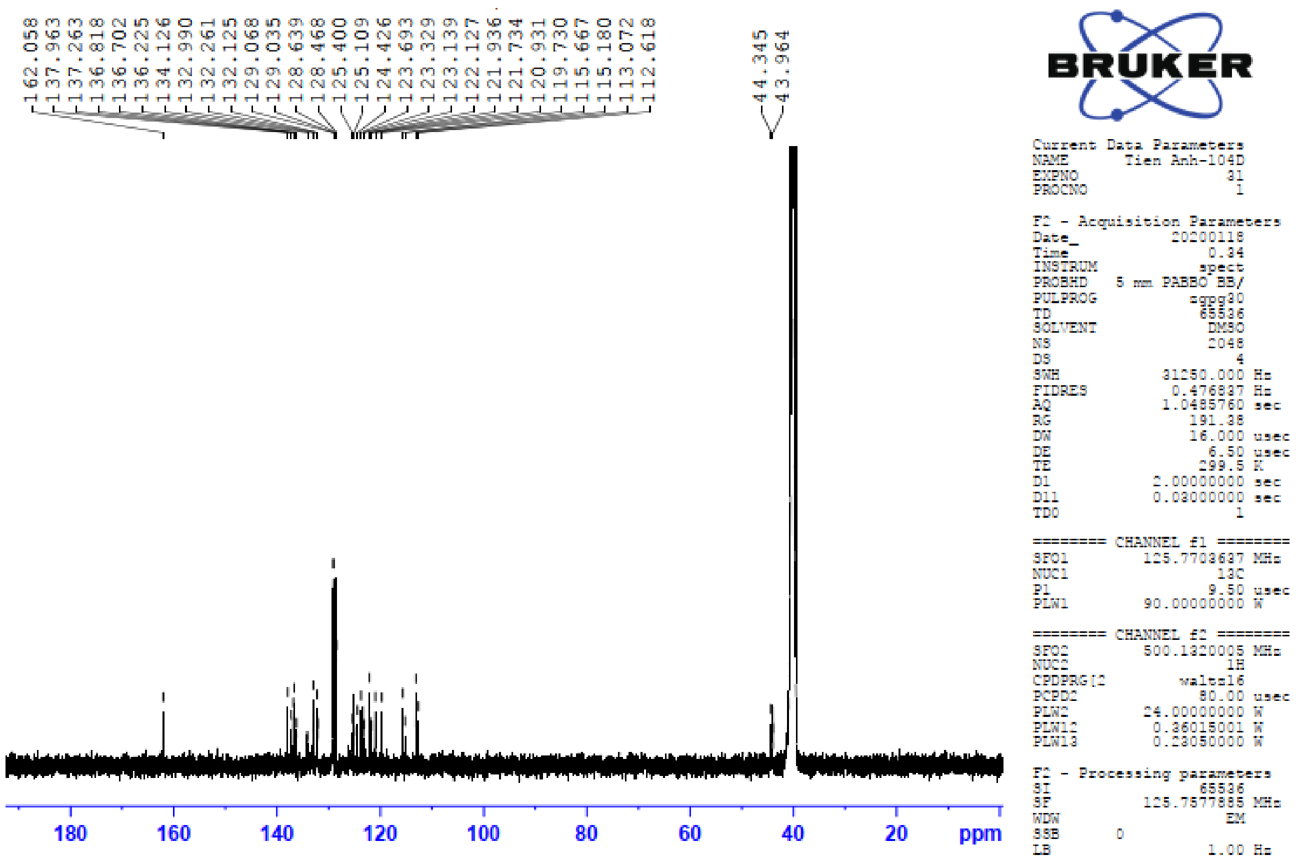

^{13}C-NMR of compound 5d

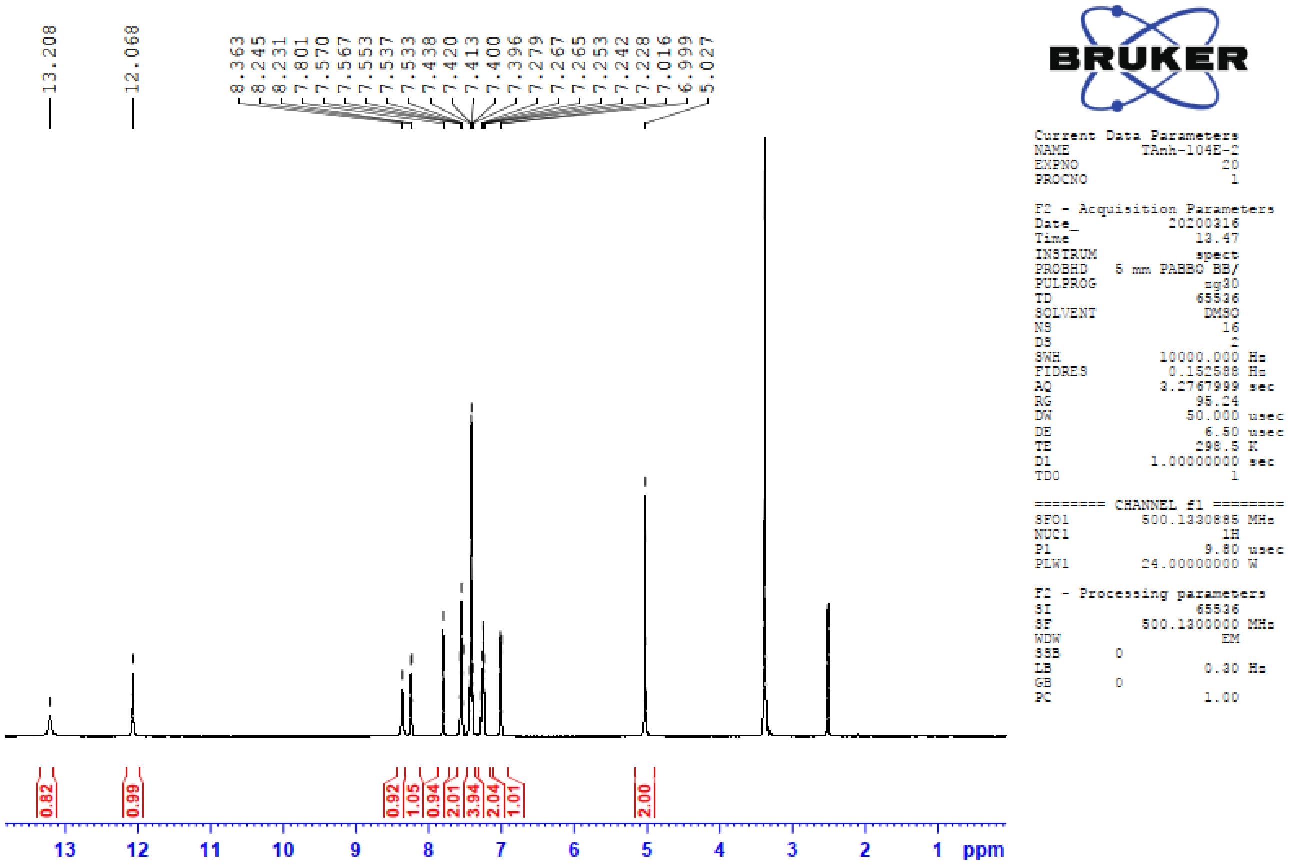

^{1}H-NMR of compound 5e

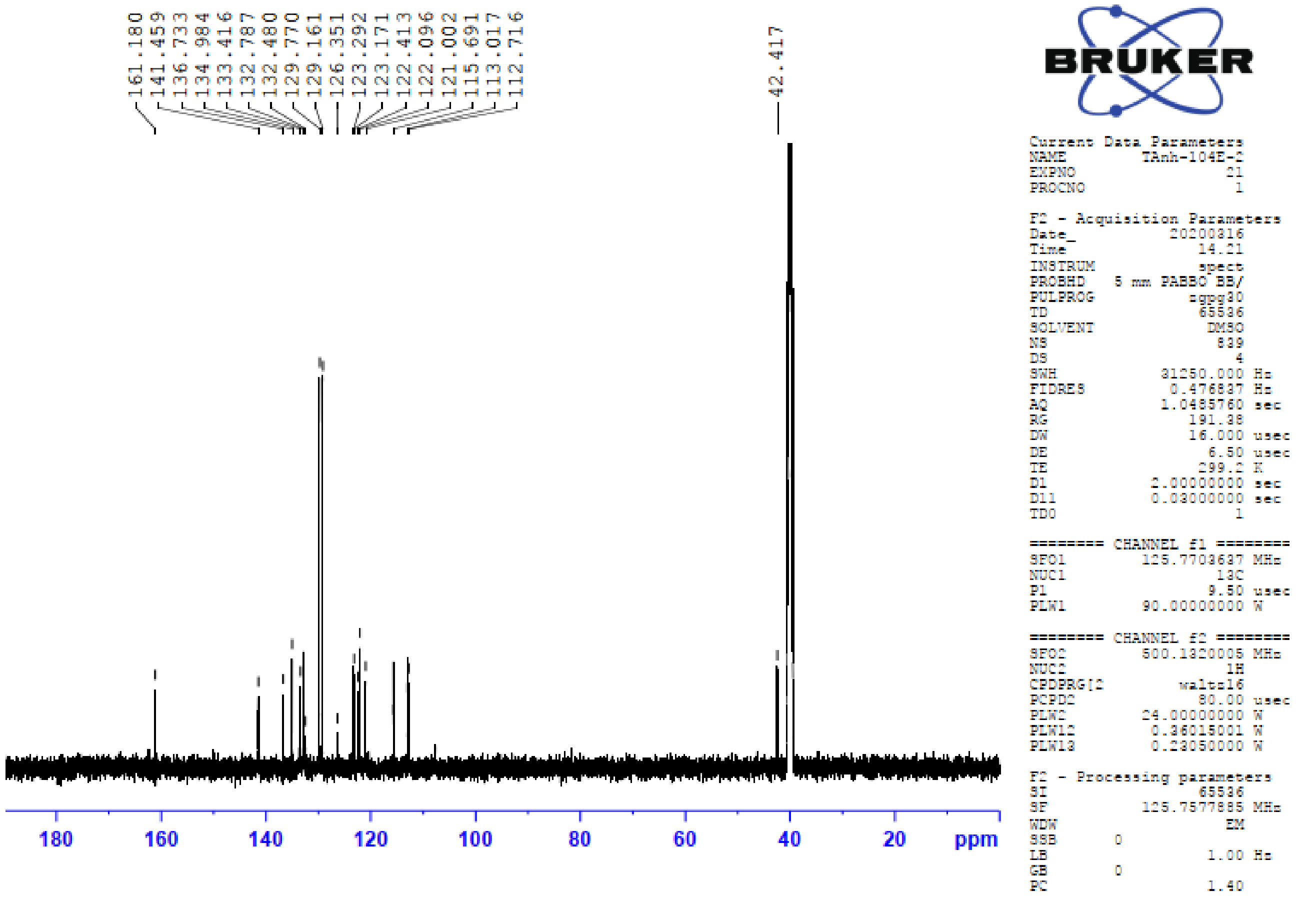

^{13}C-NMR of compound 5e

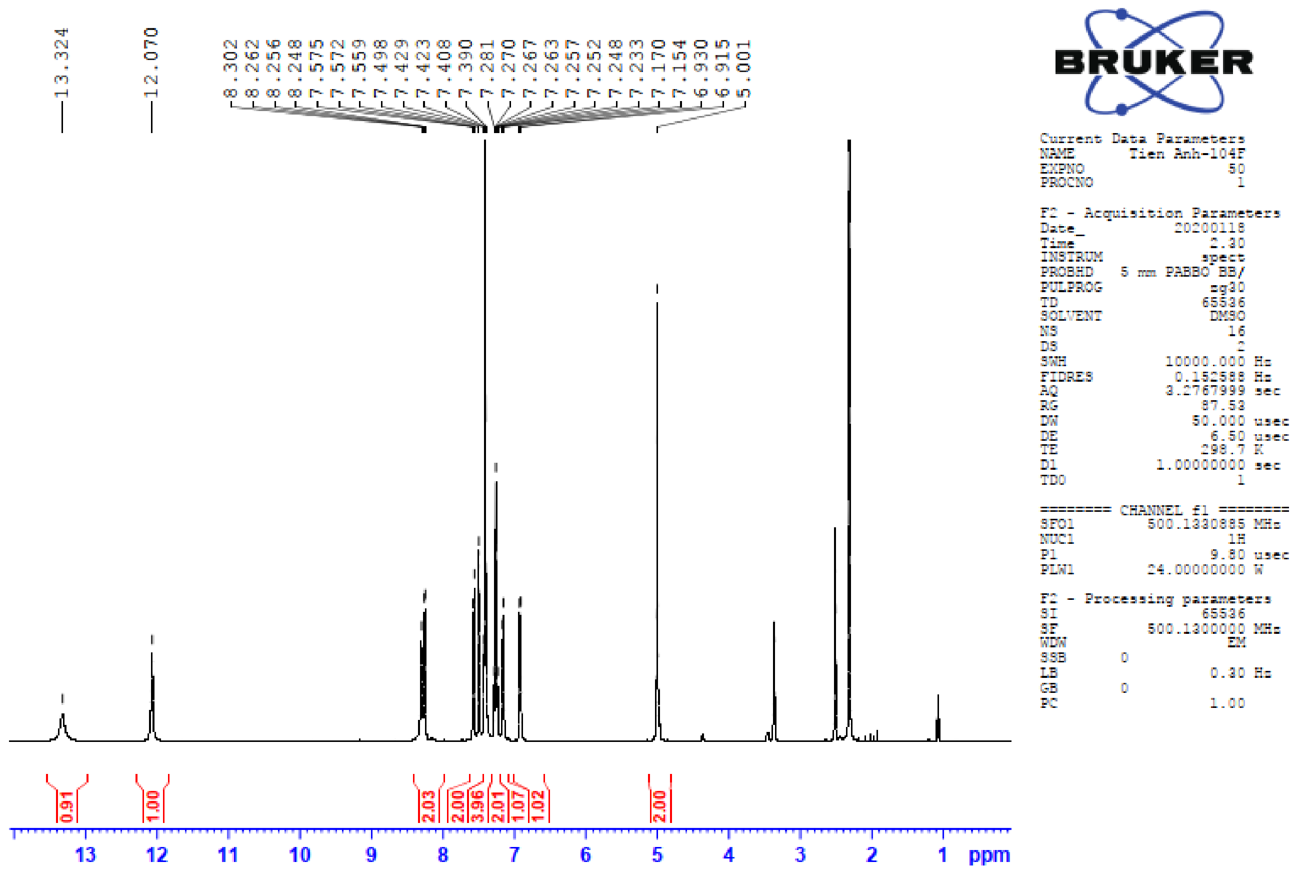

^{1}H-NMR of compound 5f

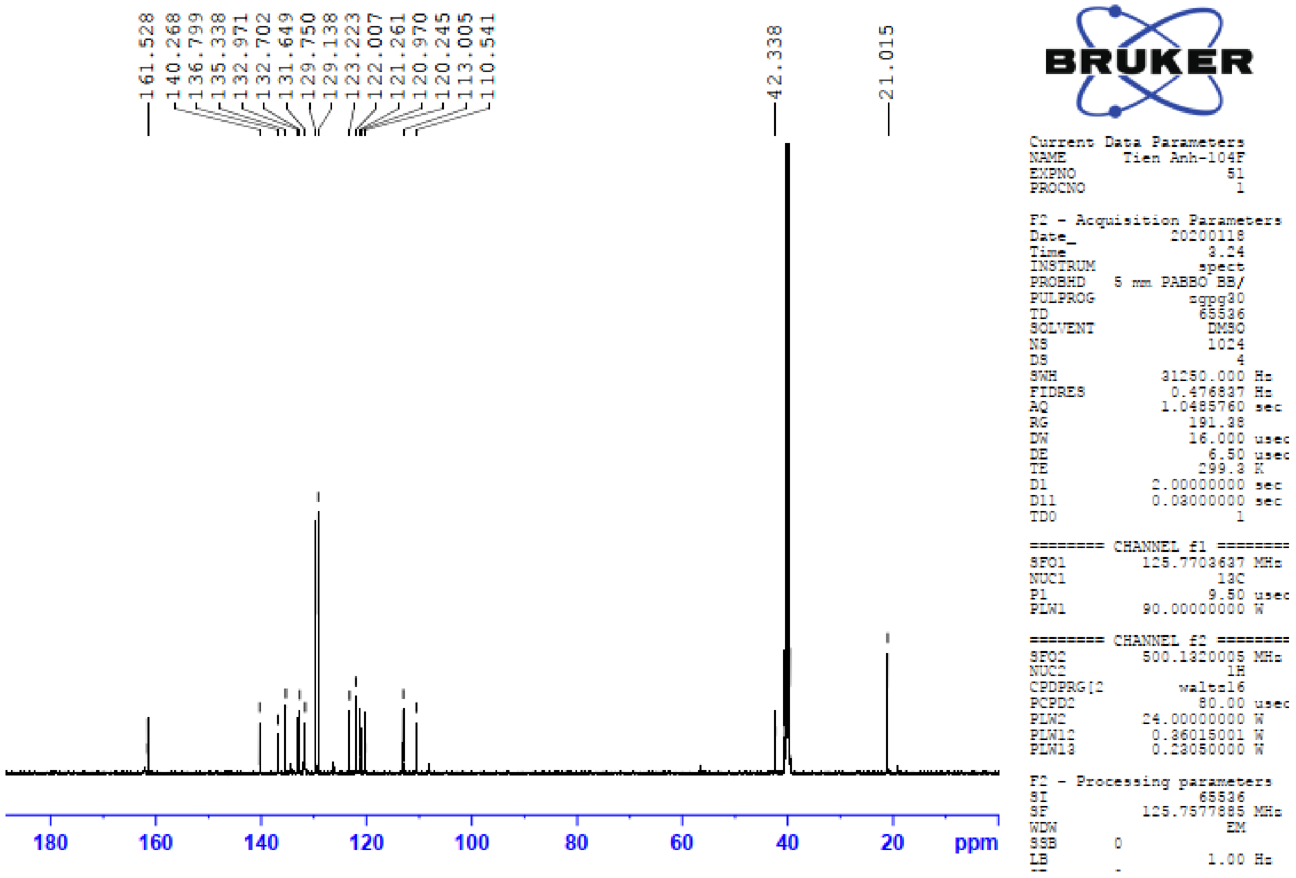

^{13}C-NMR of compound 5f

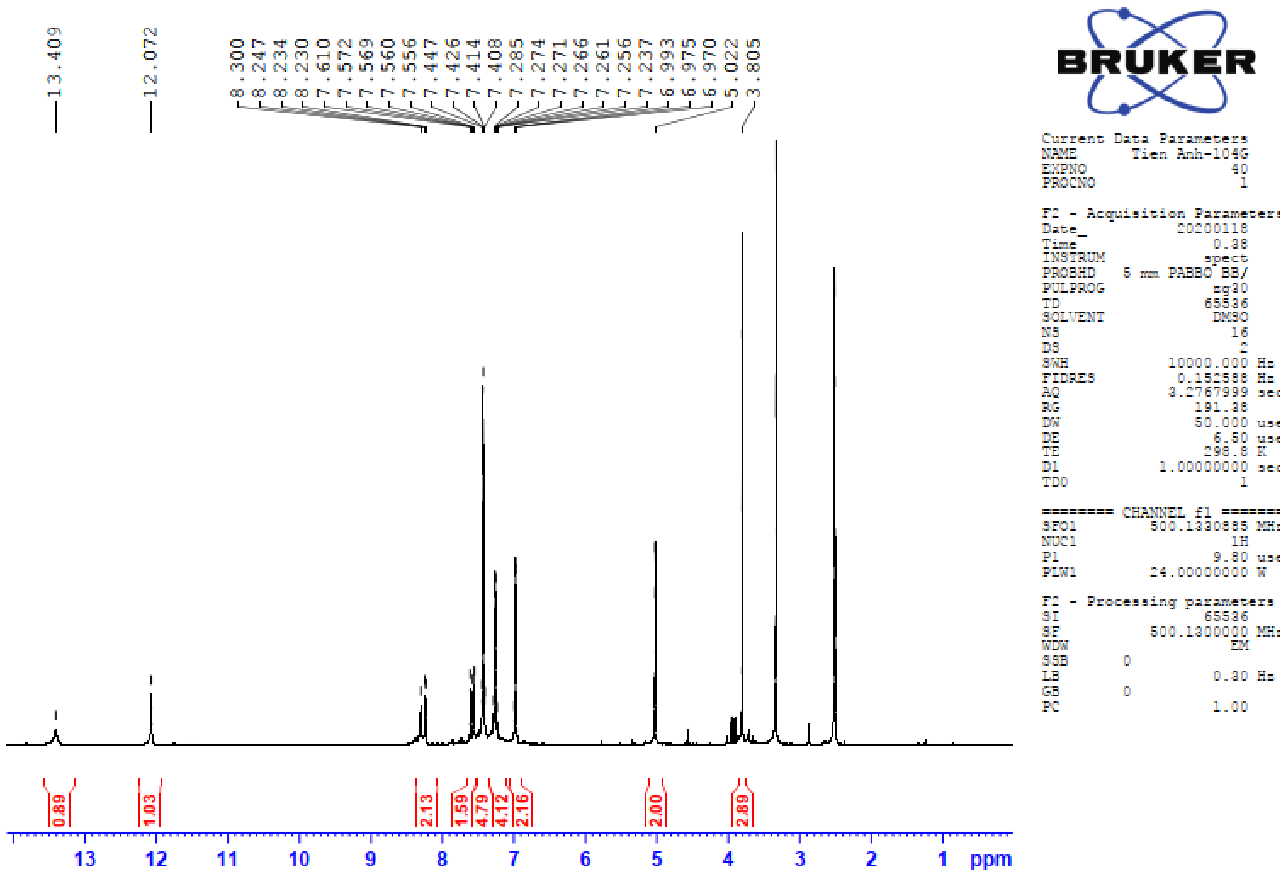

^{1}H-NMR of compound 5g

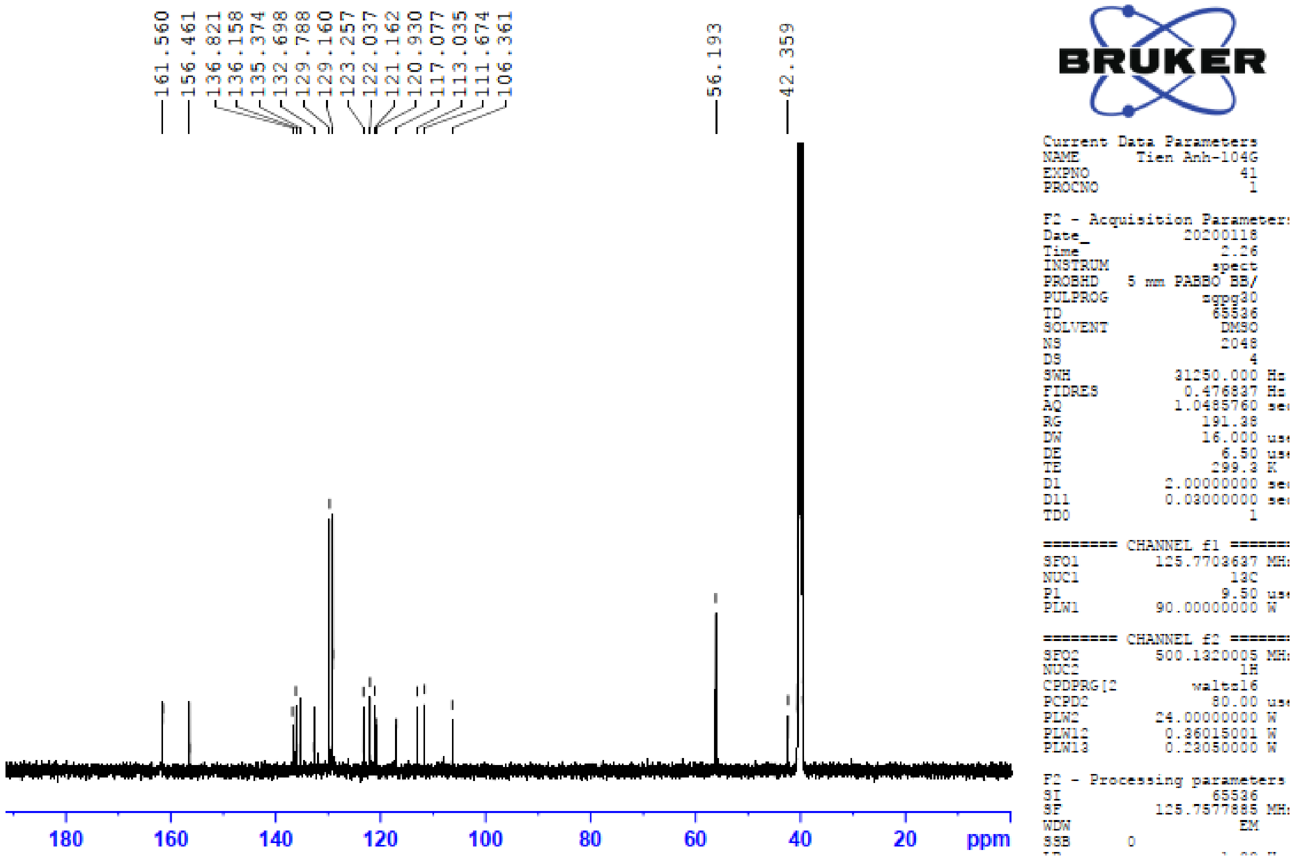

^{13}C-NMR of compound 5g
